# Discovery of the First Selective and Potent PROTAC Degrader for the Pseudokinase TRIB2

**DOI:** 10.1016/j.ejmech.2024.117016

**Published:** 2024-11-01

**Authors:** Chaowei Wen, Prathibha R. Gajjala, Yihan Liu, Bingzhong Chen, Mehtab S. Bal, Payal Sutaria, Qiao Yuanyuan, Yang Zheng, Yang Zhou, Jinwei Zhang, Weixue Huang, Xiaomei Ren, Zhen Wang, Ke Ding, Arul M. Chinnaiyan, Fengtao Zhou

**Affiliations:** a International Cooperative Laboratory of Traditional Chinese Medicine Modernization and Innovative Drug Development, Ministry of Education (MoE) of People’s Republic of China, College of Pharmacy, Jinan University, 601 Huangpu Avenue West, Guangzhou 510632, China; b Michigan Center for Translational Pathology, University of Michigan, Ann Arbor, Michigan 48109, United States; c Department of Pathology, University of Michigan, Ann Arbor, Michigan 48109, United States; d Cancer Biology Program, University of Michigan, Ann Arbor, Michigan 48109, United States; e State Key Laboratory of Chemical Biology, Shanghai Institute of Organic Chemistry, University of Chinese Academy of Sciences, Chinese Academy of Sciences, 345 Lingling Road, Shanghai 200032, China; f Rogel Cancer Center, University of Michigan, Ann Arbor, Michigan 48109, United States; g Department of Urology, University of Michigan, Ann Arbor, Michigan 48109, United States; h Howard Hughes Medical Institute, University of Michigan, Ann Arbor, Michigan 48109, United States

**Keywords:** TRIB2, Pseudokinase, PROTACs, Protein degradation, Apoptosis, Proliferation

## Abstract

Pseudokinase TRIB2, a member of the CAMK Ser/Thr protein kinase family, regulates various cellular processes through phosphorylation-independent mechanisms. Dysregulation of TRIB2 has been implicated in promoting tumor growth, metastasis, and therapy resistance, making it a promising target for cancer treatment. In this study, we designed and synthesized a series of TRIB2 PROTAC degraders by conjugating a TRIB2 binder **1** with VHL or CRBN ligands via linkers of varying lengths and compositions. Among these compounds, **5k** demonstrated potent TRIB2 degradation with a DC_50_ value of 16.84 nM (95% CI: 13.66 – 20.64 nM) in prostate cancer PC3 cells. Mechanistic studies revealed that **5k** directly interacted with TRIB2, selectively inducing its degradation through a CRBN-dependent ubiquitin-proteasomal pathway. Moreover, **5k** outperformed the TRIB2 binder alone in inhibiting cell proliferation and inducing apoptosis, confirming that TRIB2 protein degradation could be a promising therapeutic strategy for TRIB2-associated cancers. Additionally, compound **5k** also serves as an effective tool for probing TRIB2 biology.

## Graphical Abstract

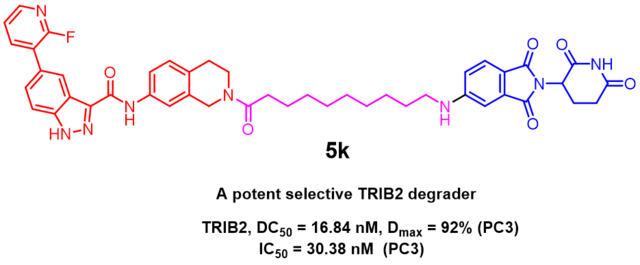

## 1. Introduction

Tribbles (TRIBs), first identified in Drosophila as key regulators of the cell cycle, belong to the CAMK subfamily in the human kinome^1^. The TRIB family includes TRIB1, TRIB2, TRIB3 and STK40^2^. TRIB2, like its family members, shares a similar structure comprising an N-terminal PEST domain, an α-C-terminal domain containing a binding motif for the COP1 ubiquitin ligase, and a central pseudokinase domain^3^. TRIB2 possesses an aberrant glycine-rich loop and deformed α-C-helix that significantly decrease its ability to catalyze phosphoryl transfer, thus categorizing it as a pseudokinase^3,4^. Consequently, unlike canonical kinases with catalytic phosphorylation activity, TRIB2 acts as a substrate adaptor, facilitating substrate ubiquitination and degradation via its COP1-binding motif^5,6^. Additionally, TRIB2 functions as a switchable scaffold for the assembly and regulation of signaling modules^7,8^. Evidence indicates that TRIB2 is integral to cellular processes such as senescence, cell cycle, and survival, interacting with various signaling molecules including CDC25C^9^, MAPK/ERK^10^, AKT^11^, OCT3/4^12^, AP4^13^, and C/EBP alpha^14^.

**Fig. 1.**
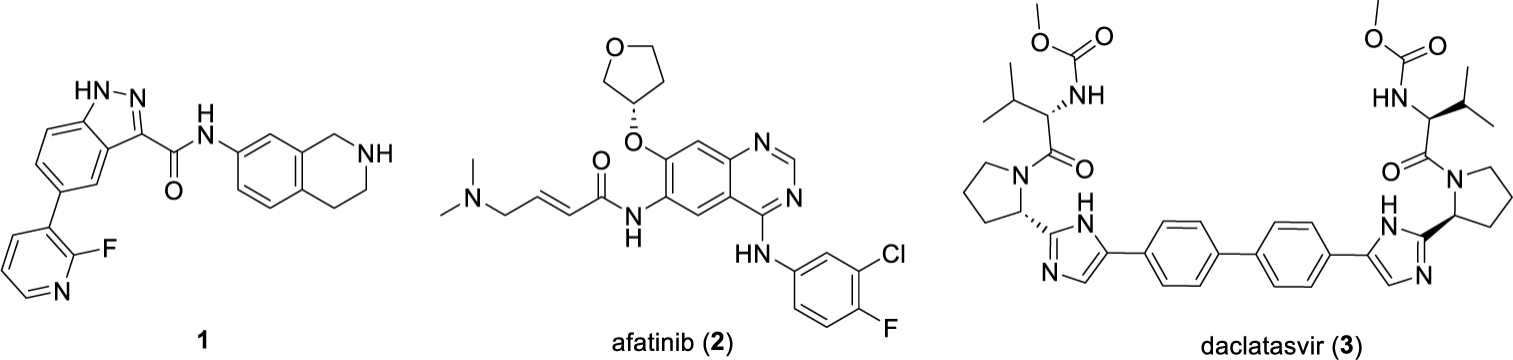
Chemical structure of TRIB2 modulators

TRIB2 is overexpressed in several types of cancers^15^, including prostate cancer^16^, liver cancer^17,18^, leukemia^2^, melanoma^19^, colorectal cancer^20^ and pancreatic cancer^21^. Overexpression of TRIB2 has been linked to poor patient survival and acquired therapeutic resistance to anticancer drugs^15^. Recent data indicate that TRIB2 is particularly overexpressed in enzalutamide-resistant and neuroendocrine (NE) prostate cancer, a major contributor to prostate cancer morbidity and mortality^16^. Consequently, TRIB2 has emerged as a promising drug target for therapy in TRIB2-associated prostate cancer^16^. However, the development of agents specifically targeting TRIB2 is hindered by the uncertain effectiveness of active-site binders as TRIB2 inhibitors. Currently, a few small-molecule binders, such as **1**^22^, have been shown to downregulate TRIB2 levels at micromolar concentration. Additionally, recent studies have demonstrated that the approved drugs afatinib (**2**)^23^, a covalent binder with TRIB2 and daclatasvir (**3**)^24^ can destabilize TRIB2 and induce its protein degradation at high micromolar concentrations, although these drugs were not specifically developed for targeting TRIB2 (Fig. 1). To date, no specific TRIB2-targeted agents have entered clinical trials for the treatment of TRIB2-associated cancers.

Recently, PROTAC-induced targeted protein degradation has emerged as a powerful tool for drug development in both industry and academia^25,26^. PROTACs are hetero-bifunctional molecules composed of three components: a ligand for the protein of interest (POI), a ligand for an E3 ligase, and a chemical linker connecting these two ligands^27^. PROTACs facilitate the degradation of target proteins by recruiting an E3 ligase in proximity to the POI, thereby triggering its ubiquitination and subsequent proteasomal degradation. This approach offers several advantages over traditional pharmacology driven by simple inhibitors^28^, particularly in its ability to deplete both enzymatic and non-enzymatic functions of target proteins, thereby broadening the scope of drug targets^29^.

Pseudokinases, which have been challenging to target using traditional inhibitors due to their lack of catalytic activity, may be particularly suitable targets for the PROTAC approach. Recent proof-of-concept studies have demonstrated the regulation of non-catalytic functions of kinases^30^ and pseudokinases^31–33^ through targeted protein degradation. A part of our ongoing efforts^34,35^ to investigate the non-enzymatic functions of kinases and pseudokinases, we utilized the protein target degradation approach to explore the non-kinase functions of pseudokinases. In this study, we report our efforts to develop the first examples of TRIB2 degrader, which exhibits potent degradation activity and significant cell growth inhibitory effects against prostate cancer cells.

## 2. Results and discussion

### 2.1. Molecular Design of TRIB2 PROTACs

**Fig. 2.**
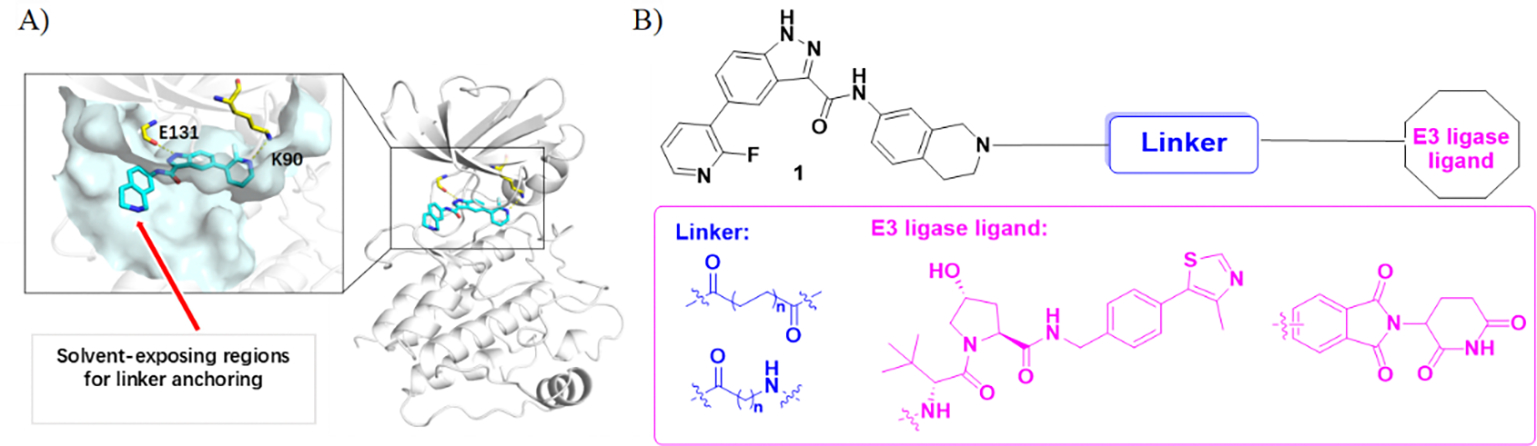
Design of TRIB2 PROTACs. (A) Docking model of **1** (green) with TRIB2 (cyan, PDB:7UPM), and the graph was polished using Pymol. (B) Design of TRIB2 PROTACs.

We selected compound **1**, discovered by KIAST^22^, as the warhead for our TRIB2 PROTACs, as it inhibits the ATP binding affinity of TRIB2 (IC_50_ = 0.74 nM) and exhibits good antiproliferative activity against A549 and HepG2 cancer cells with cell viability of 12% and 10%, respectively^22^. To understand the interaction model of compound **1** binding with TRIB2, we docked compound **1** into the ATP-binding site of TRIB2 (PDB: 7UPM) using Glide (Fig. 2A). The model indicates that the N-H of the indazole ring forms a hydrogen bond with the carbonyl group of Glu131 and the nitrogen atom of pyridine ring forms a hydrogen bond with Lys90. And the tetrahydroisoquinoline ring of compound **1** points toward the solvent, suggesting it as a suitable tether site for linkers without adversely affecting TRIB2 affinity. We then chose two types of E3 ligase ligands, namely, VHL ligand and thalidomide, respectively, which are among the most successful and widely used for inducing ubiquitination and subsequent proteasomal degradation of target proteins^36^.

Additionally, previous evidence indicates that linker composition and length are crucial for facilitating the formation of the ternary complex^37^. Therefore, we selected flexible alkyl linkers of various lengths (ranging from 2 to 12 atoms) as starting points for the design of TRIB2 PROTACs (Fig. 2B).

### 2.2. Synthesis of PROTACs targeting TRIB2

**Scheme 1.**
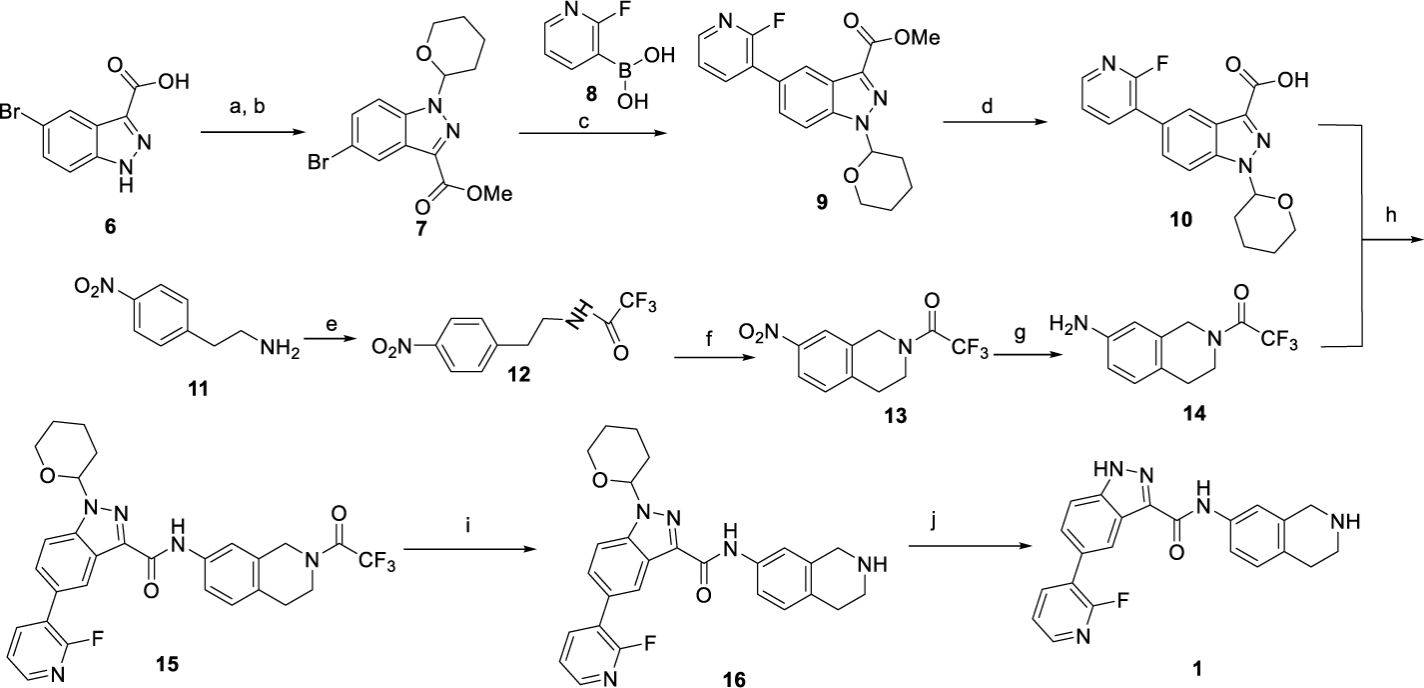
Synthesis of warhead **1**^a^ ^a^Reagents and conditions: (a) SOCl_2_, MeOH, 50°C, overnight, 90%; (b) 3,4-dihydro-2*H*-pyran (DHP), 4-toluenesulfonic acid (PTSA), 1,4-dioxane, 110 °C, reflux, 5 h, 88%; (c) Pd(PPh_3_)_2_Cl_2_, Na_2_CO_3_, 1,4-dioxane: H_2_O = 4: 1, 90°C, 2 h, 75%; (d) LiOH, THF: MeOH: H_2_O = 1: 1 :1, rt, 3 h, 90%. (e) trifluoroacetic anhydride (TFAA), triethylamine (TEA), dichloromethane (DCM), rt, 3 h, 89%. (f) (HCHO)_n_, CH_3_COOH: H_2_SO_4_ = 1: 2, 50 °C, 4 h, 90%. (g) H_2_, Pd/C (10%), rt, overnight, 95%. (h) 2-(7-azabenzotriazol-1-yl)-*N,N,N’,N’*-tetramethyluronium hexafluorophosphate (HATU), *N,N*-diisopropylethylamine (DIPEA), *N,N*-dimethylformamide (DMF), rt, 1 h, 85%; (i) LiOH, THF : MeOH : H_2_O = 4 : 2 : 1, rt, 3 h, 92%; (j) Et_3_SiH, TFA : DCM = 1 : 1, rt, overnight, 85%.

The synthetic routes for warhead **1** are illustrated in Scheme 1. Briefly, bromoindazole-3-carboxylic acid (**6**) was converted into 5-bromoindazole-3-carboxylic acid methyl ester with thionyl chloride and methanol, followed by the protection of the nitrogen atom of the indazole ring with 3,4-dihydro-2*H*-pyran (DHP) to produce compound **7**. Subsequently, a Suzuki coupling reaction of compound **7** with 2-fluoro-3-pyridineboronic acid (**8**) yielded compound **9**, which then underwent hydrolysis of the methyl ester group to produce carboxylic acid **10**. In parallel, intermediate **13** was prepared starting from 4-nitrophenylethylamine, which first underwent protection of the amine group with trifluoroacetic anhydride, followed by cyclization with paraformaldehyde. The nitro group was then reduced via palladium-catalyzed hydrogenation to deliver intermediate **14**. Next, compound **10** was coupled with **14** to give compound **15**, followed by the removal of the amide-protecting group to obtain free amine **16**. Finally, the TRIB2 binder **1** was obtained by the removal of the protecting THP group.

**Scheme 2.**
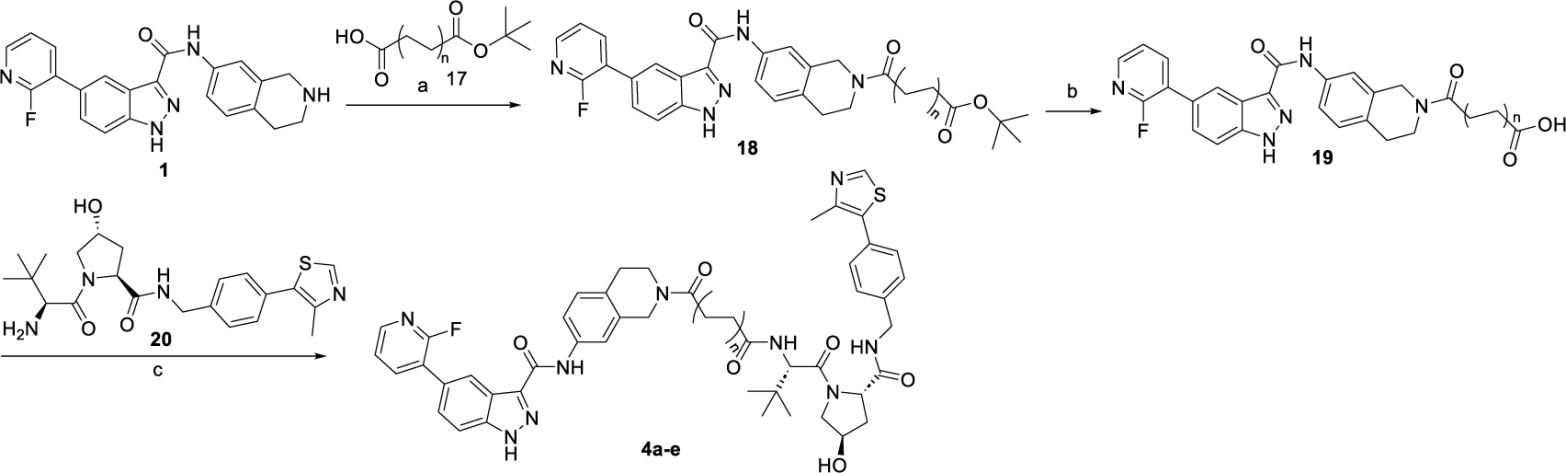
Synthesis of VHL-based PROTACs **4a-e**^a^ ^*a*^Reagents and conditions: (a) HATU, DIPEA, DMF, rt, 1 h, 40–60%; (b) TFA: DCM = 1: 1, rt, 1 h. (c) HATU, DIPEA, DMF, rt, 1 h, 65–80%.

The synthesis route for the VHL-based PROTACs **4a-e** is outlined in Scheme 2. Briefly, warhead **1** underwent amidation with dicarboxylic acid mono ^t^Bu ester **17**, followed by the deprotection of the ^t^Bu ester group to yield carboxylic acid **19**. The VHL-based PROTACs **4a-e** were then obtained by coupling the carboxylic acid **19** with VHL ligand **20**.

**Scheme 3.**
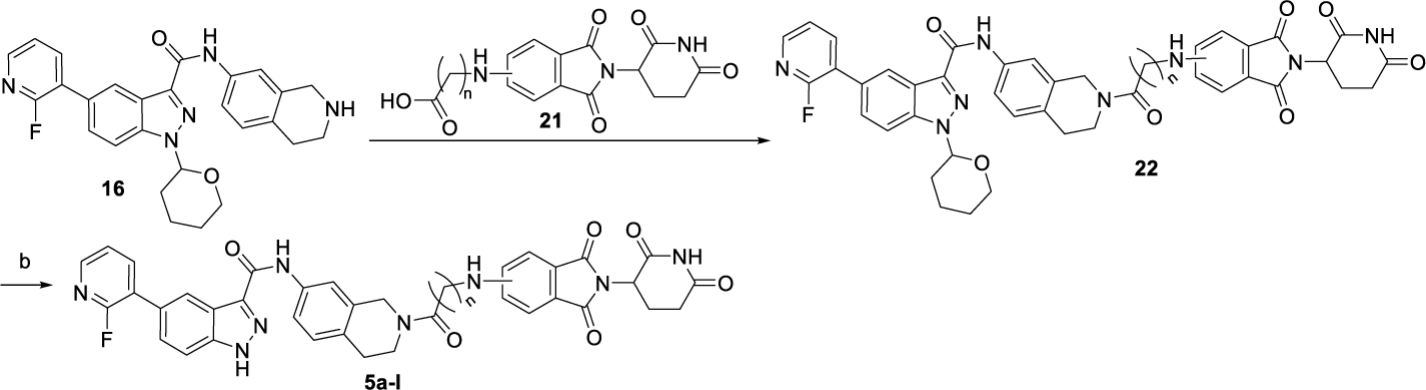
Synthesis of CRBN-based PROTACs **5a-l**^a^ ^*a*^Reagents and conditions: (a) HATU, DIPEA, DMF, rt, 1 h, 58–85%; (c) Et_3_SiH, TFA: DCM = 1: 1, rt, overnight, 80–90%.

The synthesis route for the CRBN-based PROTACs **5a-l** is shown in Scheme 3. First, warhead **16** underwent amidation with pomalidomide derivatives **21** to afford intermediate **22**. Finally, the CRBN-based PROTACs **5a-l** were obtained by removing the THP group from intermediate **22**.

**Scheme 4.**
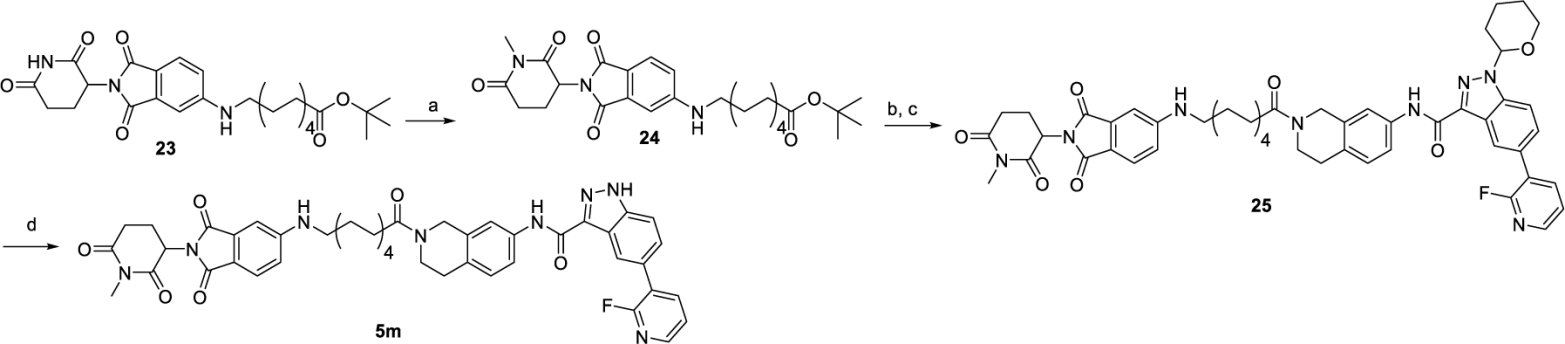
Synthesis of negative control **5m**^a^ ^*a*^Reagents and conditions: (a) CH_3_I, K_2_CO_3_, DMF, 50 °C, overnight, 75%; (b) TFA: DCM = 1 : 1, rt, 1 h; (c) **16**, HATU, DIPEA, DMF, rt, 1 h, 76%; (d) Et_3_SiH, TFA : DCM = 1 : 1, rt, overnight, 82%.

The synthesis route for the negative control compound **5m** is outlined in Scheme 4. Initially, the *N*-methylation of the glutamic imide moiety of compound **23** was performed using methyl iodide, yielding intermediate **24**. Subsequently, intermediate **24** underwent deprotection of the ^t^Bu ester group with trifluoroacetic acid, followed by direct amidation with compound **16** to produce intermediate **25**. Finally, the negative control compound **5m** was obtained by removing the THP group from intermediate **25**.

### 2.3. SAR study of the synthesized TRIB2 degraders

We chose compound **1** as warhead and conjugated it with a VHL-type ligand via a flexible alkyl linker of varying lengths (4 to 12 atoms), producing VHL-based PROTACs **4a-e**. The degradation activity of compounds **4a-e** was evaluated in the PC3 cell line, and the results are summarized in Table 1. Unfortunately, none of VHL-based PROTACs **4a-e** exhibited any degradation activity against TRIB2 in PC3 cells after 24-hour treatment at a concentration of 2 μM. Given these results, we turned our attention to another popular E3 ligase ligand pomalidomide. Pomalidomide-based PROTACs **5a-f** were synthesized by connecting warhead **1** to the amine group at the 4-position of pomalidomide using amide linkers of varying lengths. The degradation activity of these compounds was evaluated in prostate cancer PC3 cell lines, with results summarized in Table 2. Compounds **5a-f** did not exhibit significant degradation potency against TRIB2 in PC3 cells. Finally, we modified the linking site from 4-position to the 5-position of pomalidomide. The resulting compounds **5g-l** demonstrated good degradation activity against TRIB2. Among these compounds **5k** showed significant degradation activity at a concentration of 100 nM with a D_max_ value of 92%.

### 2.4. Compound 5k Induce the Degradation of TRIB2 Protein in a Dose and Time-dependent Manner

**Fig. 3.**
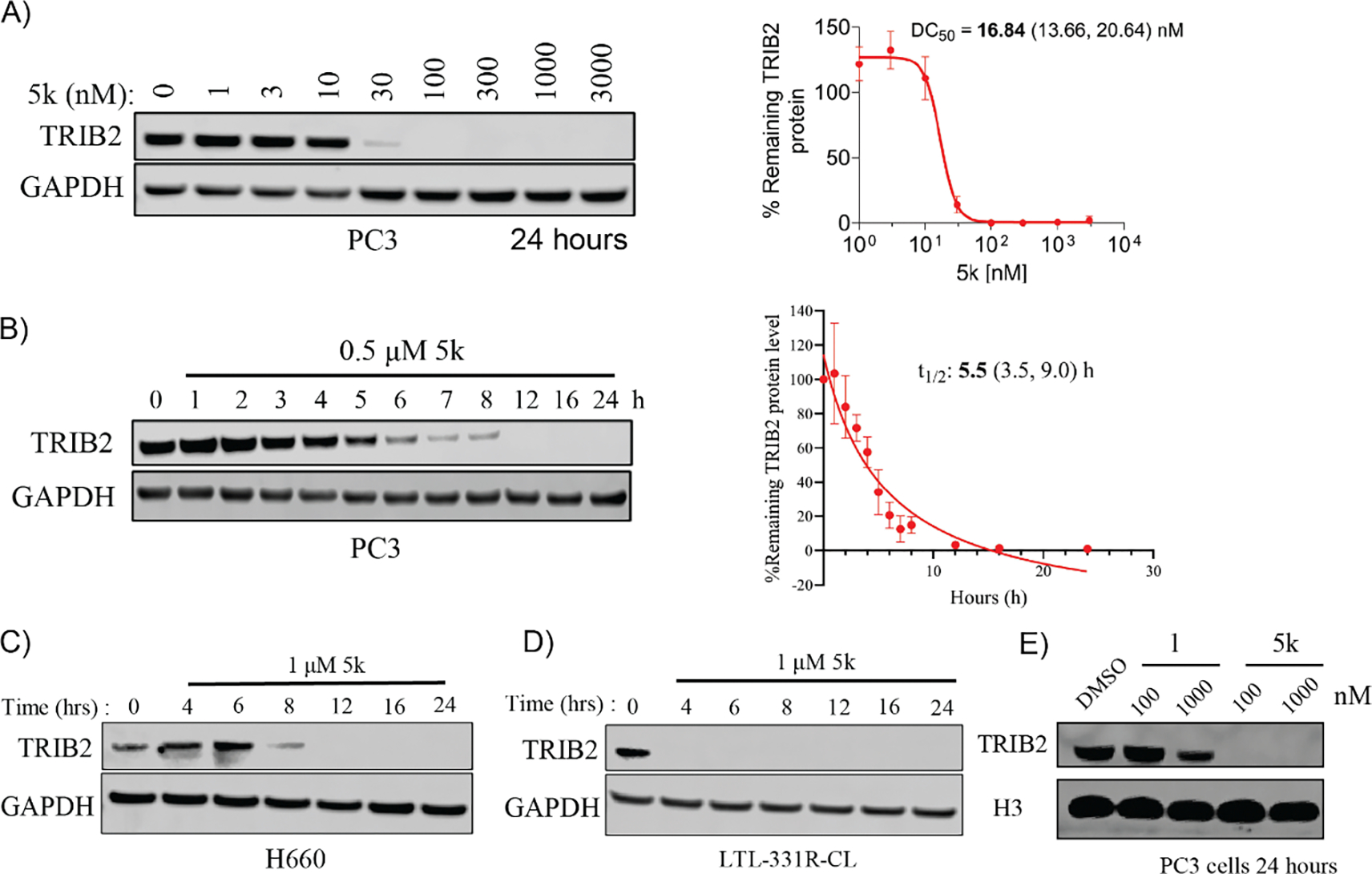
Concentration- and time-dependent degradation of TRIB2 by compound **5k** in prostate cancer cell lines. (A) Immunoblots of TRIB2 and GAPDH in PC3 cells treated with increasing concentrations of **5k** for 24 hours (left panel), followed by plotting the percentage of remaining TRIB2 protein against the concentrations of compound **5k** for DC_50_ (right panel). Error bars show standard error of the mean (SEM) from triplicate experiments. (B) Immunoblots of TRIB2 and GAPDH in PC3 cells treated with 500 nM compound **5k** for various time points (left panel), followed by quantification of the percentage of remaining TRIB2 protein to determine the t_1/2_ of TRIB2 (right panel). Error bars represent standard deviation (SD) from three independent experiments. (C, D) Immunoblots of TRIB2 and GAPDH in H660 and LTL-331R-CL cell lines treated with compound **5k** at a concentration of 1 μM for various time points; (E) Immunoblots of TRIB2 and H3 in PC3 cells treated with compound **1** and **5k** at a concentration of 100 nM and 1000 nM, respectively. Images are representative of at least two independent experiments.

As mentioned above, compound **5k** displayed potent degradation of TRIB2 protein at a concentration of 100 nM, resulting in a significant 92% degradation of TRIB2 protein. To assess the degradation activity of **5k** on TRIB2, we treated the PC3 cells with various concentrations of **5k** for 24 hours. The experimental results are shown in Fig. 3 and indicate that compound **5k** induces the degradation of TRIB2 in a dose-dependent manner with a DC_50_ value of 16.84 nM with a 95% confidence interval of 13.66 to 20.64 nM (Fig. 3A). Additionally, compound **5k** triggered rapid degradation of TRIB2 protein at a concentration of 500 nM, reaching its half-maximal degradation after 5.5 hours with a 95% confidence interval of 3.5 to 9.0 hours (Fig. 3B). Furthermore, compound **5k** also demonstrated potent degradation activity against TRIB2 protein levels in other prostate cancer cell lines including H660 and LTL-331R-CL neuroendocrine prostate cancer cells (Fig. 3C, D). We further compared the degradation activity of **1** and **5k** on TRIB2. Compound **1** induced mild TRIB2 downregulation at a concentration of 1000 nM, while **5k** led to complete TRIB2 degradation at a concentration of 100 nM (Fig. 3E).

### 2.5. Compound 5k Facilitates TRIB2 Degradation via Cereblon-dependent Ubiquitination and Proteasomal Pathways

**Fig. 4.**
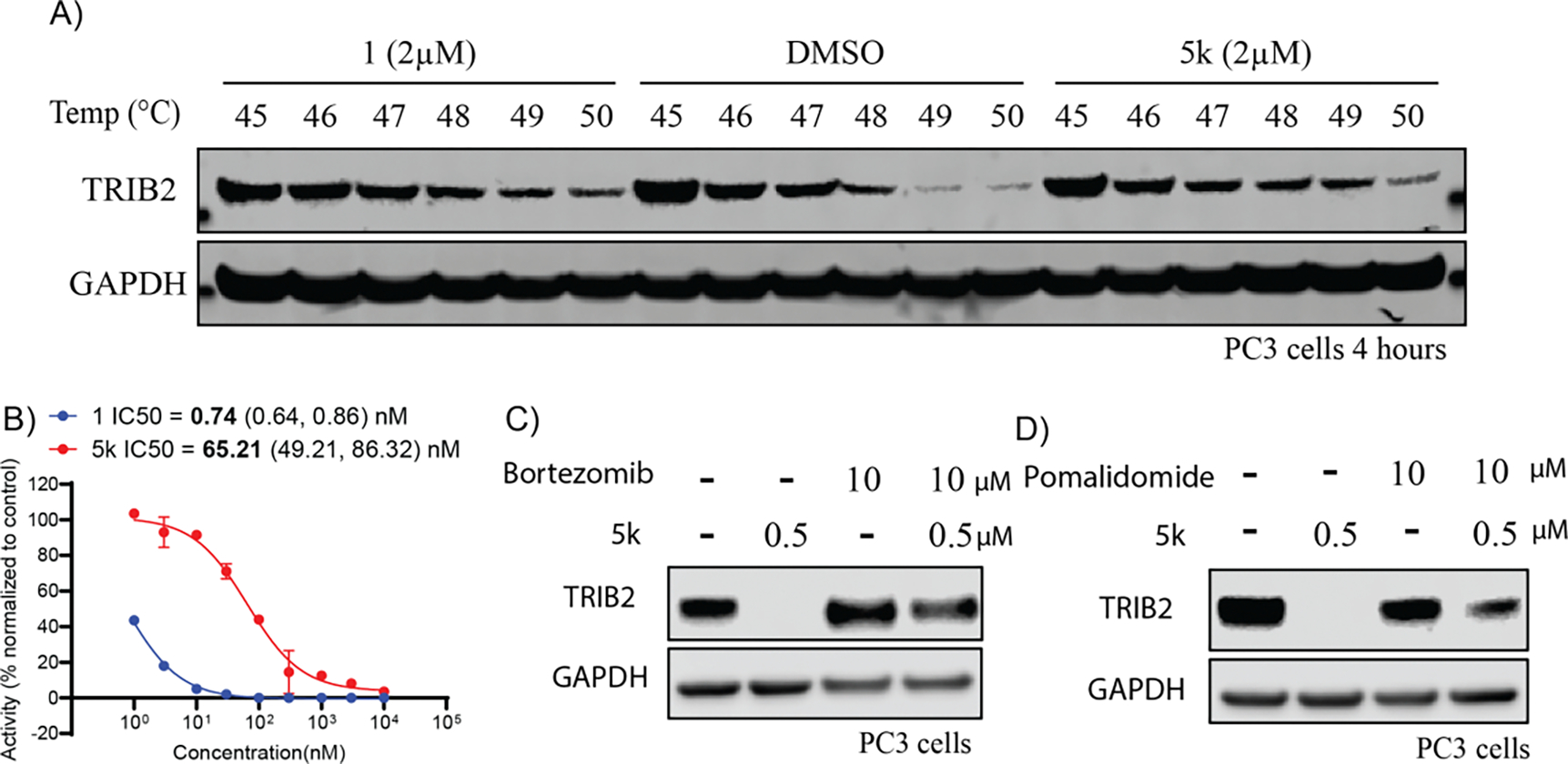
CETSA validates the binding of compound **5k** to the TRIB2 protein, and the subsequent degradation of TRIB2 mediated by compound **5k** is proteasomal and CRBN dependent. (A) Thermal shift assay (CETSA) results show the stabilization of the TRIB2 protein upon treatment with compound **5k** at a concentration of 2 μM for 3 hours. (B) Recombinant TRIB2 protein was incubated with compound **1** or **5k** and quantified for kinase activity by radiometric assay. Data are reported as the mean of two independent experiments ± SD. (C) Immunoblots of TRIB2 and GAPDH in PC3 cells were pre-treated with bortezomib at a concentration of 1 μM followed by treatment with compound **5k** at a concentration of 0.5 μM for 16 hours. (D) Immunoblots of TRIB2 and GAPDH in PC3 cells were pre-treated with pomalidomide at a concentration of 10 μM, followed by treatment with compound **5k** at a concentration of 0.5 μM for 16 hours. Images are representative of at least two independent experiments.

We further employed cellular thermal shift assay (CETSA) to evaluate the interaction of compounds **1** and **5k** and TRIB2 in PC3 cells. Immunoblotting analysis shows both compounds **1** and **5k** could stabilize the TRIB2 protein at elevated temperatures compared to vehicle control treated with DMSO (Fig. 4A), indicating that compounds **1** and **5k** could directly bind with TRIB2. Furthermore, we conducted *in vitro* radiometric kinase assay to evaluate the effect of compounds **1** and **5k** on the activity of TRIB2 (Fig. 4B). Both compound **1** (IC_50_ = 0.74 (0.64, 0.86) nM) and **5k** (IC_50_ = 65.21 (49.21, 86.32) nM) dose-dependently inhibit the ATP binding affinity of TRIB2. This result is consistent with docking model of the interaction between TRIB2 and **1**, further confirming the on-target effect of compound **1** and **5k**. Next, we explored the mechanism of action of **5k** on the degradation of TRIB2. The PC3 cells were pre-treated with bortezomib which is a proteasome inhibitor, followed by the treatment with **5k** at a concentration of 500 nM. Western blot analysis revealed that bortezomib completely rescued the levels of TRIB2 protein (Fig. 4C). Similarly, the PC3 cells were co-incubated with pomalidomide and **5k**, and pomalidomide competitively blocked the **5k**-induced degradation of TRIB2 (Fig. 4D). Moreover, the negative control compound **5m** derived from **5k** by a *N*-methylation of the -NH group in the imide moiety of **5k**, was found to have no degradation effect on TRIB2 in PC3 cells at a concentration of 100 nM (Table 2). Collectively, these results suggest that **5k** degrades the TRIB2 through the CRBN-dependent ubiquitin-proteasome pathway.

### 2.6. Global Proteomic Profiling of compound 5k

**Fig. 5.**
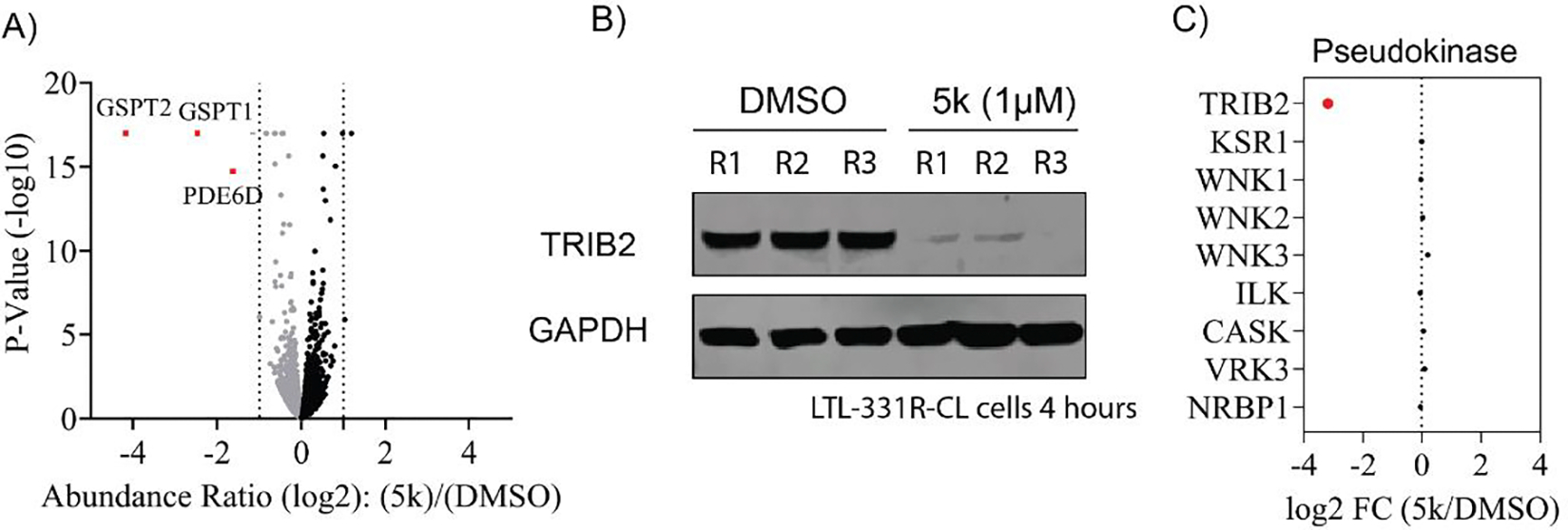
Global proteomic profiling of **5k**. (A) Unbiased global proteomics analysis of compound **5k** in LTL-331R-CL cells after the treatment with **5k** at a concentration of 1 μM for 4 hours. (B) Immunoblots of TRIB2 and GAPDH in LTL-331R-CL cells after **5k** treatment at a concentration of 1 μM for 4 hours. (C) Plot of log 2-fold change of TRIB2 and other pseudokinases based on immunoblotting and mass-spec quantification.

To further elucidate the mechanism of compound **5k**, we conducted a comprehensive proteomic analysis using a tandem mass tag (TMT) labeled mass spectrometry to quantitively assess protein alterations following 4 hours of **5k** treatment in LTL-331R-CL cells. The findings revealed that **5k** acts as a specific TRIB2 degrader, with only two significantly down-regulated proteins identified as off-targets among 6286 detectable proteins (Fig. 5A). While immunoblots of TRIB2 from protein lysates of the same samples validated that TRIB2 was significantly degraded by **5k** under this condition (Fig. 5B), TRIB2 was not detected in the proteomic analysis (potentially due to protein abundance). We further plotted the log 2-fold change of TRIB2 and other pseudokinases based on mass-spectrometry and immunoblots quantification (Fig. 5C). Notably, **5k** exhibited selectivity towards TRIB2 over other pseudokinases. Collectively, the data implied that **5k** is a selective TRIB2 degrader.

### 2.7. 5k Showed Potent Anti-proliferative and Apoptotic Effects in AR^−^ Prostate Cancer Cell Lines.

**Fig. 6.**
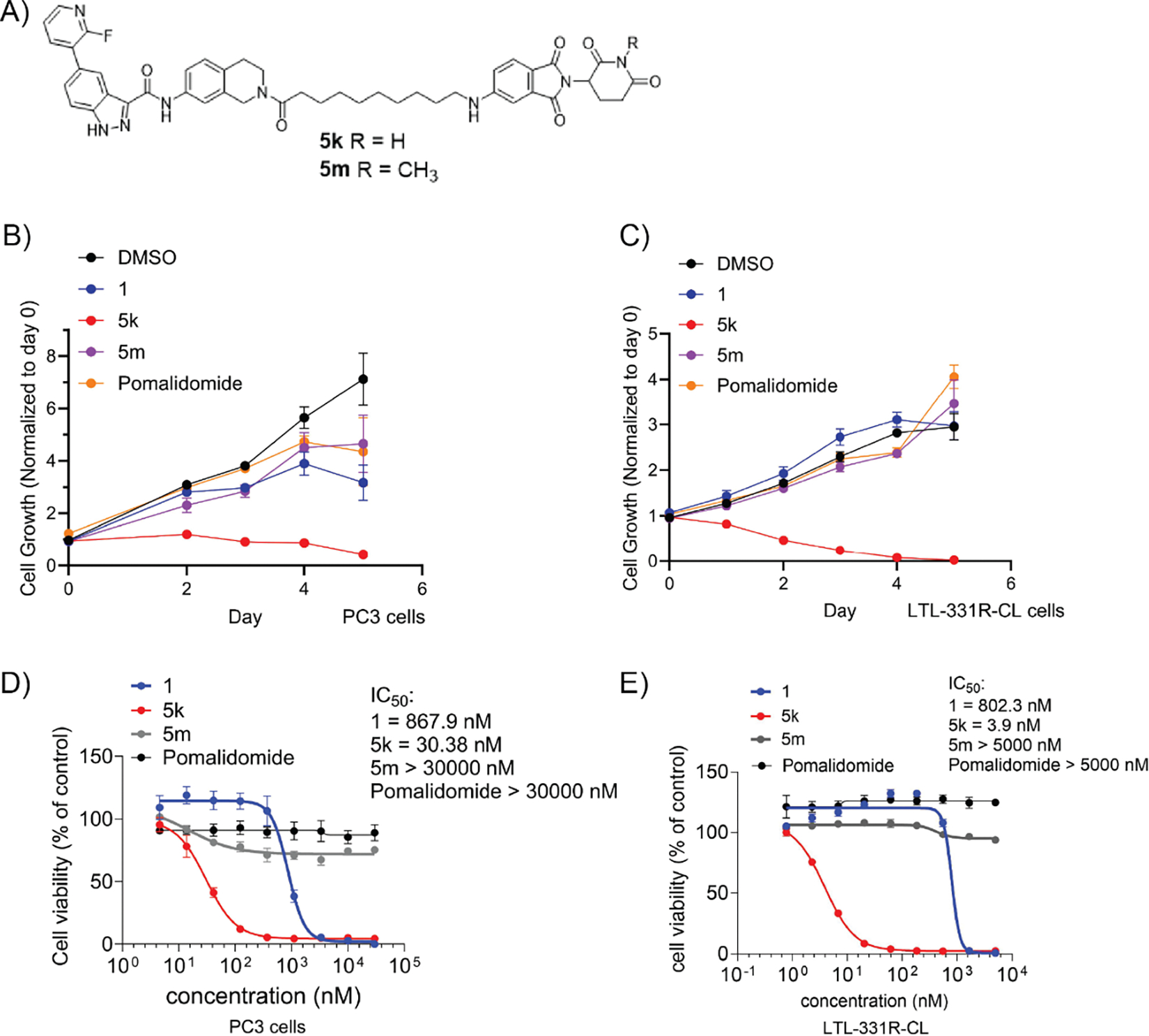
Compound **5k** shows antiproliferative effects and induces apoptosis in prostate cancer cell lines. (A) Chemical structure of **5k** and **5m**. (B, C) PC3 or LTL-331R-CL cells were treated with **1**, **5k**, **5m**, pomalidomide at a concentration of 1 μM or DMSO. Cell viability was quantified by CellTiter-Glo assay on different days. Data are reported as the means of four independent experiments ± SD. (D, E). PC3 and LTL-331R-CL cells were treated with **1**, **5k**, **5m**, or pomalidomide at varying concentrations for 5 days and quantified for cell viability by CellTiter-Glo assay. Data are reported as the means of four independent experiments ± SD.

Prostate cancer, particularly those with neuroendocrine characteristics, represents a significant challenge in clinical management, accounting for a substantial portion of prostate cancer-related mortality^16^. Recent studies have identified TRIB2 as a promising therapeutic target for TRIB2-associated prostate cancer treatment^16,24^. Compound **5k** demonstrated excellent degradation activity in prostate cancer PC3 cells, promoting further evaluation of its anti-proliferative effects across a panel of AR^−^ prostate cancer cells using CTG assay. The results showed that **5k** exhibited potent antiproliferative effects compared to **1**, **5m** (Fig. 6A), pomalidomide, and DMSO in both PC3 and LTL-331R-CL cell lines (Fig. 6B, C). Moreover, the determination of IC_50_ values for compounds **1**, **5k**, **5m**, and pomalidomide in PC3 and LTL-331R-CL cell lines revealed that neuroendocrine-like LTL-331R-CL cells were more sensitive to **5k**, with IC_50_ values in the nanomolar range (Fig. 6D, E). In PC3 cells, the dose-dependent cell viability is highly correlated with the TRIB2 degradation profile, suggesting that the cytotoxic effect is strongly associated with the loss of TRIB2. In contrast, **5m** and pomalidomide showed no impact on cell viability.

**Fig. 7.**
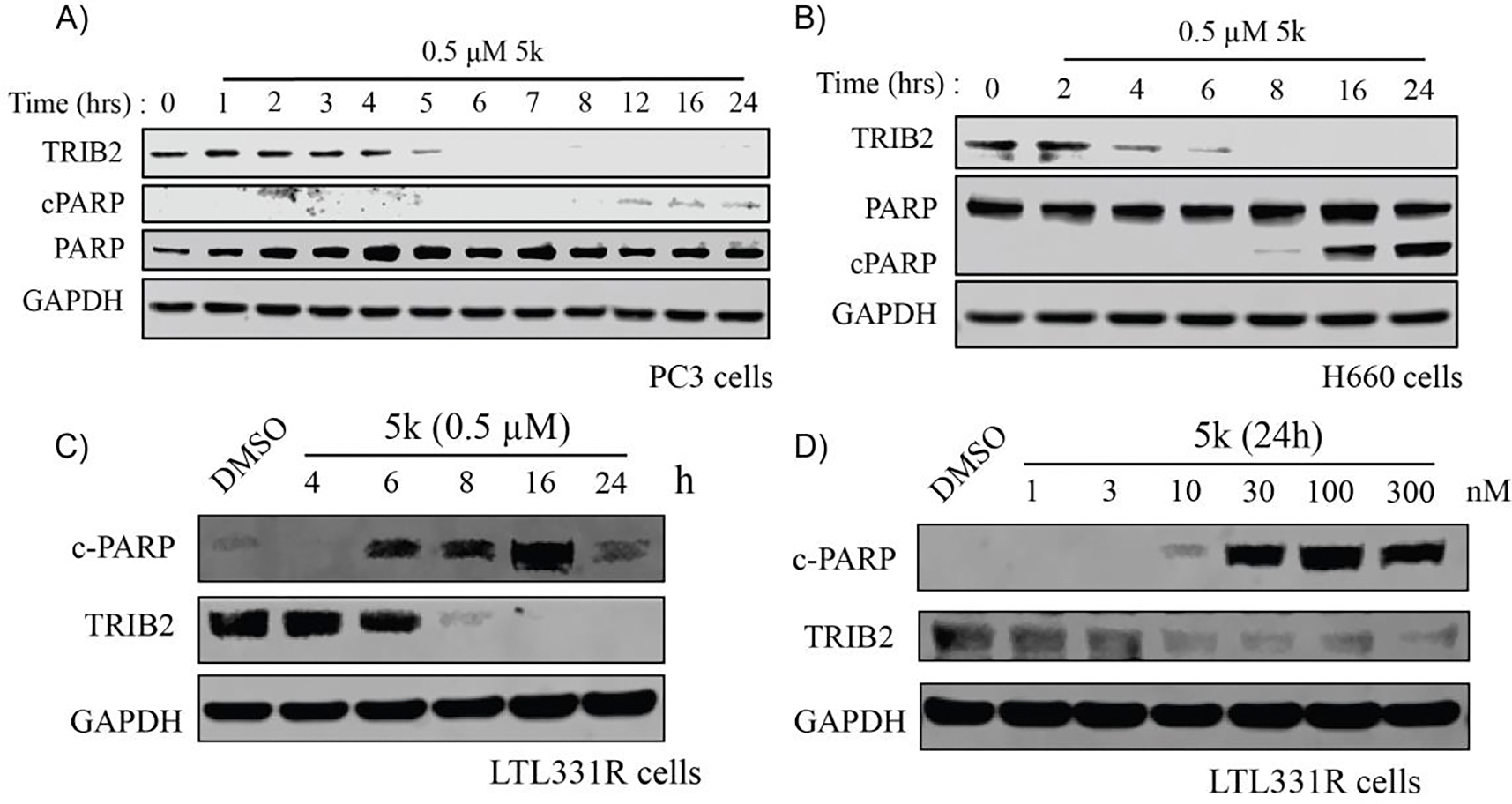
Compound **5k** triggers apoptosis in a panel of prostate cancer cell lines. (A) Immunoblots of TRIB2, cPARP, PARP and GAPDH in PC3 cells treated with compound **5k** at a concentration of 0.5 μM for the annotated time durations. (B) Immunoblots of TRIB2, cPARP, PARP and GAPDH in H660 cells treated with 0.5 μM of compound **5k** for increasing time durations. (C) Immunoblots of TRIB2, cPARP, and GAPDH in LTL-331R-CL cells treated with 0.5 μM of **5k** for the annotated time durations. (D) Immunoblots of TRIB2, cPARP, and GAPDH in LTL-331R-CL cells treated with increasing concentrations of **5k** for 24 hours. Images are representative of at least two independent experiments.

To elucidate the mechanism of action of **5k**, further experiments demonstrated that **5k** induced increased cleavage of PARP in a dose-and time-dependent manner in various AR^−^ cell lines, including PC3, H660 and LTL-331R-CL (Fig. 7A–D). This suggests that **5k** induces apoptosis, highlighting its potential as a therapeutic agent for TRIB2-associated prostate cancer, particularly in neuroendocrine-like contexts where currently treatment options are limited. Interestingly, TRIB2 is not an essential gene in the cell lines analyzed in the Cancer Dependency Map (DepMap)^38^. In comparison to the knockout models in DepMap, **5k** induces an acute and complete loss of TRIB2. Previous studies have shown that TRIB2 functions as a scaffold protein involved in various cancer-related signaling pathways^13^. Our observations suggest that the acute loss of TRIB2 could more severely disrupt these pathways, resulting in stronger cytotoxic effects. In contrast, to the TRIB2 knockout dependency in Depmap, Additionally, the cytotoxicity of **5k** may also partially be attributed to inducing the degradation of GSPT1, a requisite release factor for the translation termination that functions as an oncogenic driver in several types of cancer. GSPT1 degraders such as CC-90009 display potency in several types of cancer cells^39^. These findings underscore the promising role of TRIB2-targeted degradation in addressing unmet clinical needs in prostate cancer therapy.

## 3. Conclusions

In conclusion, TRIB2 plays a critical role in regulating key cellular processes, such as proliferation and survival. Its dysregulation is implicated in various diseases, particularly cancer, where it promotes tumor growth, metastasis, and resistance to therapy. The multifaceted role of TRIB2 in oncogenesis highlights its potential as a promising therapeutic target for cancer treatment. While drugs like afatinib and daclatasvir have shown the ability to reduce TRIB2 protein levels, their effectiveness is limited to high micromolar concentrations. Consequently, the development of TRIB2-targeting PROTACs represents a highly promising avenue for novel cancer therapies. Here, we report the discovery of a TRIB2 degrader **5k** through a PROTACs approach. Compound **5k** demonstrated potent degradation of TRIB2 in PC3 cells with a DC_50_ value of 16.84 nM (95% CI: 13.66 – 20.64 nM). Mechanistic studies revealed that **5k** directly interacts with TRIB2 and potently induces TRIB2 degradation through CRBN-dependent ubiquitin-proteasomal pathways. Furthermore, **5k** exhibited significant anti-proliferative effects and induced apoptosis, consistent with effects observed upon genetic depletion of TRIB2. Compound **5k** can also serve as a tool for studying the biology of TRIB2. Overall, TRIB2-targeted degradation with compounds like **5k** represents a promising avenue for advancing cancer treatment strategies.

## 4. Experimental Section

### 4.1. General Methods for Chemistry

All commercially available reagents and solvents were used without further purification. Chemical reactions were monitored by thin-layer chromatography (TLC) with visualization under UV light at 254 or 365 nm. ^1^H NMR spectra were obtained using a Bruker AV-400/600 spectrometer, while ^13^C NMR spectra were recorded on a Bruker AV-600 spectrometer at 150 MHz, with tetramethylsilane (TMS) or a deuterated solvent as the internal reference. Low-resolution mass spectra (MS) were recorded on an Agilent 1200 HPLC-MSD mass spectrometer, and high-resolution mass spectra were acquired using an Applied Biosystems Q-STAR Elite ESI-LC-MS/MS mass spectrometer. The purity of all final compounds was confirmed to be greater than 95% by HPLC analysis, conducted with an Agilent 1260 system. The analytical columns used were YMC-Triart C18 reversed-phase columns (5 μm, 4.6 mm × 250 mm) with a flow rate of 1.0 mL/min.

#### 4.1.1. Methyl 5-bromo-1-(tetrahydro-2*H*-pyran-2-yl)-1*H*-indazole-3 carboxylate (7)

Dichlorosulfoxide (12.1 mL, 166.70 mmol) was added to a solution of 5-bromo-1*H*-indazole-3-carboxylic acid **6** (20.000 g, 82.90 mmol) in methanol (300.0 mL). The reaction mixture was heated overnight at 50 °C. The solvent was evaporated under reduced pressure, and the residue was purified by silica gel column chromatography, yielding methyl 5-bromo-1*H*-indazole-3-carboxylate (19.000 g, 90% yield). To a solution of methyl methyl 5-bromo-1*H*-indazole-3-carboxylate (6.500 g, 25.59 mmol) and *p*-toluenesulfonic acid (348 mg, 2.0 mmol) in 1,4-dioxane (300.0 mL) was added 3,4-dihydro-2*H*-pyran (4.7 mL, 51.18 mmol). The reaction mixture was then heated overnight at 110 °C. The solvent was evaporated under reduced pressure, and the residue was purified by silica gel column chromatography to afford the title compound **7** (7.610 g, 88% yield). ^1^H NMR (400 MHz, CDCl_3_) *δ* 8.40 (d, *J* = 1.8 Hz, 1H), 7.62 (d, *J* = 8.9 Hz, 1H), 7.53 (dd, *J* = 8.9, 1.8 Hz, 1H), 5.82 (dd, *J* = 9.3, 2.6 Hz, 1H), 4.05 (s, 3H), 4.03 (s, 1H), 3.83–3.48 (m, 1H), 2.62–2.38 (m, 1H), 1.85–1.68 (m, 2H).

#### 4.1.2. Methyl 5-(2-fluoropyridin-3-yl)-1-(tetrahydro-2*H*-pyran-2-yl)-1*H*-indazole-3-carbo-xylate (9)

Compound **7** (10.000 g, 29.59 mmol), anhydrous sodium carbonate (6.270 g, 59.17 mmol), Pd(PPh_3_)_2_Cl_2_ (2.100 g, 3.0 mmol) and (2-fluoropyridin-3-yl)boronic acid **8** (4.590 g, 32.54 mmol) were added to a solution of 1,4-dioxane (200.0 mL) and H_2_O (50.0 mL). The resulting suspension was evacuated and backfilled with argon three times. The reaction mixture was evaporated under reduced pressure and the residue was purified by silica gel column chromatography to afford compound **9** as a white solid (7.880 g, 75% yield). ^1^H NMR (400 MHz, DMSO-*d*_6_) *δ* 8.30 (s, 1H), 8.27 (d, *J* = 4.9 Hz, 1H), 8.22–8.15 (m, 1H), 7.99 (d, *J* = 8.8 Hz, 1H), 7.74 (d, *J* = 8.8 Hz, 1H), 7.54–7.43 (m, 1H), 6.04 (dd, *J* = 9.5, 2.5 Hz, 1H), 3.99–3.88 (m, 1H), 3.85–3.70 (m, 1H), 2.47–2.32 (m, 1H), 2.19–1.96 (m, 2H), 1.90–1.68 (m, 1H), 1.72–1.53 (m, 2H).

#### 4.1.3. 5-(2-fluoropyridin-3-yl)-1-(tetrahydro-2*H*-pyran-2-yl)-1*H*-indazole-3-carboxylic acid (10)

To a solution of intermediate **9** (16.000 g, 45.20 mmol) in THF (150 mL), H_2_O (150.0 mL) and MeOH (150.0 mL), LiOH-H_2_O (7.600 g, 180.80 mmol) was added. The mixture was stirred at room temperature overnight. The solvent was evaporated under reduced pressure. Water (150.0 mL) was added to the residue, and the pH was adjusted to 2 using 1 M HCl. The resulting precipitate was collected by filtration and dried to yield compound **10** as a white solid (13.8 g, 90% yield). ^1^H NMR (400 MHz, DMSO-*d*_6_) *δ* 13.28 (s, 1H), 8.30 (s, 1H), 8.27 (d, *J* = 4.9 Hz, 1H), 8.22–8.15 (m, 1H), 7.99 (d, *J* = 8.8 Hz, 1H), 7.74 (d, *J* = 8.8 Hz, 1H), 7.54–7.43 (m, 1H), 6.04 (dd, *J* = 9.5, 2.5 Hz, 1H), 3.99–3.88 (m, 1H), 3.85–3.70 (m, 1H), 2.47–2.32 (m, 1H), 2.19–1.96 (m, 2H), 1.90–1.68 (m, 1H), 1.72–1.53 (m, 2H).

#### 4.1.4. 2,2,2-trifluoro-*N*-(4-nitrophenethyl) acetamide (12)

Trifluoroacetic anhydride (8.3 mL, 58.76 mmol) was added to a solution of triethylamine (18.8 mL, 135.60 mmol) and 4-nitrophenethylamine **11** (15.000 g, 90.40 mmol) in dichloromethane (DCM, 250.0 mL). The mixture was stirred at room temperature for 3 hours. The solvent was then evaporated under reduced pressure, and the residue was purified by silica gel column chromatography to yield the title compound **12** (21.000 g, 89% yield). ^1^H NMR (400 MHz, CDCl_3_) *δ* 8.16 (d, *J* = 8.4 Hz, 2H), 7.38 (d, *J* = 8.3 Hz, 2H), 6.80 (s, 1H), 3.68 (q, *J* = 6.8 Hz, 2H), 3.04 (t, *J* = 7.1 Hz, 2H).

#### 4.1.5. 2,2,2-trifluoro-1-(7-nitro-3,4-dihydroisoquinolin-2(1*H*)-yl)ethan-1-one (13)

To a solution of acetic acid (125.0 mL) and sulfuric acid (235.0 mL, 98%), intermediate **12** (23.650 g, 90.26 mmol) and paraformaldehyde (4.330 g, 149.40 mmol) were added in an ice bath. The mixture was then heated at 50 °C for 3 hours. The reaction mixture was poured into a beaker containing 300 mL of ice water. The mixture was extracted three times with ethyl acetate. The combined organic phases were washed with water and saturated sodium carbonate, then dried over anhydrous sodium sulfate. The solvent was evaporated under reduced pressure, and the residue was purified by silica gel column chromatography to yield the title compound **13** (22.300 g, 90% yield). ^1^H NMR (400 MHz, DMSO-*d*_6_) δ 8.49–8.17 (m, 1H), 8.07 (t, *J* = 8.5 Hz, 1H), 7.50 (d, *J* = 8.5 Hz, 1H), 5.10–4.66 (m, 2H), 3.85 (t, *J* = 4.7 Hz, 2H), 3.05 (t, *J* = 6.7 Hz, 2H).

#### 4.1.6. 1-(7-amino-3,4-dihydroisoquinolin-2(1*H*)-yl)-2,2,2-trifluoroethan-1-one (14)

Intermediate **13** (22.300 g, 81.2 mmol) was added to a solution of Pd/C (2.200 g, 10%) in methanol (250.0 mL). The resulting suspension was evacuated and backfilled with hydrogen for three times. The reaction mixture was stirred at room temperature under a hydrogen atmosphere overnight. After the completion of hydrogenation, the reaction mixture was filtered, and the filtrate was evaporated under reduced pressure. The residue was purified by silica gel column chromatography to afford the title compound **14** (18.820 g, 95% yield). ^1^H NMR (400 MHz, DMSO-*d*_6_) *δ* 8.49–8.17 (m, 1H), 8.07 (t, *J* = 8.5 Hz, 1H), 7.50 (d, *J* = 8.5 Hz, 1H), 5.10–4.66 (m, 2H), 3.85 (t, *J* = 4.7 Hz, 2H), 3.05 (t, *J* = 6.7 Hz, 2H).

#### 4.1.7. 5-(2-fluoropyridin-3-yl)-1-(tetrahydro-2*H*-pyran-2-yl)-*N*-(2-(2,2,2-trifluoroacetyl)-1,2,3,4-tetrahydroisoquinolin-7-yl)-1*H*-indazole-3-carboxamide (15)

To a solution of intermediate **10** (15.330 g, 43.06 mmol), intermediate **14** (10.000 g, 41.00 mmol), and HATU (17.130 g, 45.08 mmol) in DMF (80.0 mL), DIPEA (23.6 mL, 135.26 mol) was added. The mixture was stirred at room temperature for 1 hour. The solvent was then evaporated under reduced pressure, and the residue was purified by silica gel column chromatography to afford the title compound **15** (20.400 g, 84% yield). ^1^H NMR (400 MHz, DMSO-*d*_6_) *δ* 10.28 (s, 1H), 8.45 (s, 1H), 8.27 (d, *J* = 4.4 Hz, 1H), 8.23–8.15 (m, 1H), 8.01 (d, *J* = 8.8 Hz, 1H), 7.88–7.65 (m, 3H), 7.51 (t, *J* = 7.0, 4.8, 1.7 Hz, 1H), 7.21 (t, *J* = 8.3, 6.0 Hz, 1H), 6.04 (dd, *J* = 10.0, 2.4 Hz, 1H), 4.81–4.58 (m, 2H), 4.08–3.93 (m, 1H), 3.92–3.76 (m, 3H), 2.97–2.78 (m, 2H), 2.66–2.55 (m, 1H), 2.17–2.01 (m, 2H), 1.93–1.73 (m, 1H), 1.75–1.51 (m, 2H).

#### 4.1.8. 5-(2-fluoropyridin-3-yl)-1-(tetrahydro-2*H*-pyran-2-yl)-*N*-(1,2,3,4-tetrahydroisoquinolin-7-yl)-1*H*-indazole-3-carboxamide (16)

To a solution of intermediate **15** (5.500 g, 9.70 mmol) in H_2_O (40.0 mL), methanol (80.0 mL) and THF (160.0 mL), LiOH-H_2_O (1.630 g, 38.80 mmol) was added. The reaction mixture was stirred at room temperature for 3 hours. The solvent was then evaporated under reduced pressure, and the residue was purified by silica gel column chromatography to afford the title compound **16** as a white solid (4.200 g, 92% yield). ^1^H NMR (400 MHz, DMSO-*d*_6_) *δ* 10.14 (s, 1H), 8.45 (s, 1H), 8.27 (d, *J* = 4.8 Hz, 1H), 8.19 (t, *J* = 9.9, 7.4, 1.9 Hz, 1H), 7.99 (d, *J* = 8.8 Hz, 1H), 7.75 (d, 1H), 7.62–7.55 (m, 2H), 7.53–7.43 (m, 1H), 7.05 (d, *J* = 8.2 Hz, 1H), 6.03 (dd, *J* = 9.8, 2.3 Hz, 1H), 4.01–3.92 (m, 1H), 3.93–3.77 (m, 3H), 2.96 (t, *J* = 5.9 Hz, 2H), 2.70–2.62 (m, 3H), 2.18–1.93 (m, 2H), 1.90–1.75 (m, 1H), 1.72–1.57 (m, 2H).

#### 4.1.9. 5-(2-fluoropyridin-3-yl)-*N*-(1,2,3,4-tetrahydroisoquinolin-7-yl)-1*H*-indazole-3-carboxamide (1)

Trifluoroacetic acid (20.0 mL) and triethyl silane (2.9 mL, 17.84 mol) were added to a solution of intermediate **16** (4.200 g, 8.92 mmol) in DCM (20.0 mL). The reaction mixture was stirred at room temperature overnight. The solvent was evaporated under reduced pressure and the residue was purified by silica gel column chromatography to afford the title compound **1** (2.930 g, 85% yield). ^1^H NMR (400 MHz, DMSO-*d*_6_) *δ* 10.23 (s, 1H), 8.45 (s, 1H), 8.27 (dd, *J* = 3.9, 2.3 Hz, 1H), 8.20 (m, 1H), 7.80 (d, *J* = 8.7 Hz, 1H), 7.70 (dt, *J* = 8.6, 1.8 Hz, 1H), 7.64 (d, *J* = 2.2 Hz, 1H), 7.56 (dd, *J* = 8.3, 2.2 Hz, 1H), 7.51 (ddd, *J* = 7.0, 4.8, 1.8 Hz, 1H), 7.03 (d, *J* = 8.3 Hz, 1H), 3.84 (s, 2H), 2.95 (t, *J* = 5.9 Hz, 2H), 2.65 (t, *J* = 5.9 Hz, 2H). ^13^C NMR (151 MHz, DMSO-*d*_6_) *δ* 161.29, 160.17 (d, *J*_*C-F*_ = 236.5 Hz), 146.71 (d, *J*_*C-F*_ = 15.6 Hz), 142.11 (d, *J*_*C-F*_ = 5.0 Hz), 141.37, 138.97, 137.62, 129.70, 129.45, 128.17, 128.14, 127.62, 123.66 (d, *J*_*C-F*_ = 28.2 Hz), 123.26 (d, *J*_*C-F*_ = 4.4 Hz), 122.50, 122.33 (d, *J*_*C-F*_ = 3.6 Hz), 120.15, 118.51, 111.99, 44.27, 41.25, 24.80. HRMS (ESI) for C_22_H_19_ON_5_F [M+H]^+^, calcd: 388.1568; found: 388.1559. HPLC purity: 98.7%, 6.95 min.

#### 4.1.10. *tert*-butyl 4-(7-(5-(2-fluoropyridin-3-yl)-1*H*-indazole-3-carboxamido)-3,4-dihydroiso-quinolin-2(1*H*)-yl)-4-oxobutanoate (18a)

A solution of compound **1** (1.000 g, 2.58 mmol), *tert*-butyl 4-amino-4-oxobutanoate **17a** (536 mg, 3.10 mmol), and HATU (1.180 g, 3.10 mmol) in DMF (20.0 mL) was prepared, to which DIPEA (1.4 mL, 7.74 mol) was added. The reaction mixture was stirred at room temperature for 1 hour, then evaporated under reduced pressure. The residue was purified by silica gel column chromatography to afford the title compound **18a** (702 mg, 50% yield). ^1^H NMR (400 MHz, DMSO-*d*_6_) *δ* 13.95 (s, 1H), 10.65–9.95 (m, 1H), 8.45 (s, 1H), 8.27 (d, *J* = 5.0 Hz, 1H), 8.24–8.12 (m, 1H), 7.90–7.58 (m, 4H), 7.52 (t, *J* = 5.0, 2.4 Hz, 1H), 7.20–7.10 (m, 1H), 4.63 (d, *J* = 30.3 Hz, 2H), 3.68 (q, *J* = 6.1 Hz, 2H), 2.84 (t, *J* = 6.0 Hz, 1H), 2.73 (t, *J* = 5.9 Hz, 1H), 2.66–2.57 (m, 2H), 2.47–2.41 (m, 2H), 1.37 (s, 9H).

#### 4.1.11. *tert*-butyl 6-(7-(5-(2-fluoropyridin-3-yl)-1*H*-indazole-3-carboxamido)-3,4-dihydroiso-quinolin-2(1*H*)-yl)-6-oxohexanoate (18b)

Compound **18b** was synthesized using the same procedure as for **18a**. ^1^H NMR (400 MHz, DMSO-*d*_6_) *δ* 13.95 (s, 1H), 10.65–9.95 (m, 1H), 8.45 (s, 1H), 8.27 (d, *J* = 5.0 Hz, 1H), 8.24–8.12 (m, 1H), 7.90–7.58 (m, 4H), 7.52 (t, *J* = 5.0, 2.4 Hz, 1H), 7.20–7.10 (m, 1H), 4.63 (d, *J* = 30.3 Hz, 2H), 3.68 (q, *J* = 6.1 Hz, 2H), 2.84 (t, *J* = 6.0 Hz, 1H), 2.73 (t, *J* = 5.9 Hz, 1H), 2.66–2.57 (m, 2H), 2.47–2.41 (m, 2H), 1.37 (s, 9H).

#### 4.1.12. *tert*-butyl 8-(7-(5-(2-fluoropyridin-3-yl)-1*H*-indazole-3-carboxamido)-3,4-dihydroiso-quinolin-2(1*H*)-yl)-8-oxooctanoate (18c)

Compound **18c** was synthesized using the same procedure as for **18a**. ^1^H NMR (400 MHz, DMSO-*d*_6_) *δ* 13.95 (s, 1H), 10.65–9.86 (m, 1H), 8.45 (s, 1H), 8.27 (d, *J* = 4.8 Hz, 1H), 8.20 (dd, *J* = 10.2, 7.6 Hz, 1H), 7.91–7.57 (m, 4H), 7.55–7.41 (m, 1H), 7.19–7.10 (m, 1H), 4.63 (d, *J* = 23.5 Hz, 2H), 3.68 (t, *J* = 6.1 Hz, 2H), 2.82 (t, *J* = 6.0 Hz, 1H), 2.76–2.62 (m, 1H), 2.45–2.36 (m, 2H), 2.27–2.11 (m, 2H), 1.61–1.43 (m, 5H), 1.46–1.33 (m, 12H).

#### 4.1.13. *tert*-butyl 10-(7-(5-(2-fluoropyridin-3-yl)-1*H*-indazole-3-carboxamido)-3,4-dihydrois-oquinolin-2(1*H*)-yl)-10-oxodecanoate (18d)

Compound **18d** was synthesized using the same procedure as for **18a**. ^1^H NMR (400 MHz, DMSO-*d*_6_) *δ* 13.95 (s, 1H), 10.50–10.02 (m, 1H), 8.45 (s, 1H), 8.27 (d, *J* = 4.7 Hz, 1H), 8.19 (t, *J* = 9.0 Hz, 1H), 7.94–7.57 (m, 4H), 7.51 (t, *J* = 6.1 Hz, 1H), 7.24–7.10 (m, 1H), 4.62 (d, *J* = 23.1 Hz, 2H), 3.67 (s, 2H), 2.81 (t, *J* = 6.1 Hz, 1H), 2.76–2.62 (m, 1H), 2.44–2.32 (m, 2H), 2.15 (q, *J* = 7.2 Hz, 2H), 1.58–1.42 (m, 5H), 1.41–1.33 (m, 10H), 1.31–1.19 (m, 6H).

#### 4.1.14. *tert*-butyl 12-(7-(5-(2-fluoropyridin-3-yl)-1*H*-indazole-3-carboxamido)-3,4-dihydro-isoquinolin-2(1*H*)-yl)-12-oxododecanoate (18e)

Compound **18e** was synthesized using the same procedure as for **18a**. ^1^H NMR (400 MHz, DMSO-*d*_6_) *δ* 13.95 (s, 1H), 10.35 (d, *J* = 7.2 Hz, 1H), 8.45 (s, 1H), 8.27 (d, *J* = 4.8 Hz, 1H), 8.22 (s, 1H), 7.94–7.54 (m, 4H), 7.51 (t, *J* = 6.2 Hz, 1H), 7.24–7.01 (m, 1H), 4.62 (d, *J* = 23.3 Hz, 1H) , 3.68 (s, 2H), 2.83 (t, *J* = 6.1 Hz, 1H), 2.74–2.61 (m, 1H), 2.44–2.32 (m, 2H), 2.16 (q, *J* = 7.2 Hz, 2H), 1.58–1.42 (m, 5H), 1.40–1.32 (m, 11H), 1.31–1.19 (m, 9H).

#### 4.1.15. 5-(2-fluoropyridin-3-yl)-*N*-(2-(4-(((*S*)-1-((2*S*,4*R*)-4-hydroxy-2-((4-(4-methylthiazol-5-yl)benzyl)carbamoyl)pyrrolidin-1-yl)-3,3-dimethyl-1-oxobutan-2-yl)amino)-4-oxobutanoyl)-1,2,3,4-tetrahydroisoquinolin-7-yl)-1*H*-indazole-3-carboxamide (4a)

To a solution of intermediate **18a** (150 mg, 0.28 mmol) in DCM (3.0 mL), trifluoroacetic acid (3.0 mL) was added. The reaction mixture was stirred at room temperature for one hour. The mixture was then evaporated under reduced pressure, and the residue was dissolved in DMF (5.0 mL). To this solution, compound **20** (119 mg, 0.28 mmol), HATU (126.0 mg, 0.28 mmol), and DIPEA (0.2 mL, 0.84 mmol) were added. The reaction mixture was stirred at room temperature for one hour and then water (15.0 mL) was added, resulting in the formation of precipitates, which were collected by filtration. The residue was purified by silica gel column chromatography to afford the title compound **4a** (171.2 mg, 69.0% yield). ^1^H NMR (400 MHz, DMSO-*d*_6_) *δ* 13.93 (s, 1H), 10.36 (d, *J* = 8.7 Hz, 1H), 8.98 (s, 1H), 8.57 (t, *J* = 6.2 Hz, 1H), 8.45 (s, 1H), 8.26 (d, *J* = 4.7 Hz, 1H), 8.19 (dd, *J* = 10.2, 7.7 Hz, 1H), 7.94 (d, *J* = 9.2 Hz, 1H), 7.80 (d, *J* = 8.8 Hz, 1H), 7.77–7.59 (m, 3H), 7.55–7.46 (m, 1H), 7.40 (q, *J* = 8.1 Hz, 4H), 7.15 (dd, *J* = 9.1, 3.8 Hz, 1H), 5.13 (s, 1H), 4.68 (s, 1H), 4.60 (s, 1H), 4.54 (d, *J* = 9.3 Hz, 1H), 4.43 (q, *J* = 7.2 Hz, 2H), 4.36 (s, 1H), 4.23 (dd, *J* = 15.9, 5.5 Hz, 1H), 3.76–3.56 (m, 4H), 2.83 (d, *J* = 6.1 Hz, 1H), 2.77–2.53 (m, 4H), 2.44 (s, 3H), 2.15–2.00 (m, 1H), 1.99–1.81 (m, 1H), 0.94 (s, 9H). ^13^C NMR (151 MHz, DMSO-*d*_6_) *δ* 172.44, 171.83, 170.87, 170.83, 161.16, 160.18 (d, *J*_*C-F*_ = 236.8 Hz), 151.92, 148.18, 146.73 (d, *J*_*C-F*_ = 14.8 Hz), 142.13 (d, *J*_*C-F*_ = 3.0 Hz), 142.10, 141.35, 139.97, 139.21, 137.36, 137.31, 134.28, 133.87, 131.64, 130.23, 130.11, 129.11, 128.19, 127.90, 123.68 (d, *J*_*C-F*_ = 28.1 Hz), 123.25 (d, *J*_*C-F*_ = 4.0 Hz), 122.52 (d, *J*_*C-F*_ = 2.6 Hz), 122.43, 119.31, 119.14, 118.39, 118.37, 111.86, 69.38, 59.20, 56.95, 56.77, 46.98, 44.27, 43.12, 42.13, 38.38, 35.82, 30.54, 28.68, 26.85, 16.41. HRMS (ESI) for C_48_H_50_O_6_N_9_FNaS [M+Na]^+^, calcd: 922.3481; found: 922.3455. HPLC purity: 98.2%, 5.25 min.

#### 4.1.16. 5-(2-fluoropyridin-3-yl)-*N*-(2-(6-(((*S*)-1-((2*S*,4*R*)-4-hydroxy-2-((4-(4-methylthiazol-5-yl)benzyl)carbamoyl)pyrrolidin-1-yl)-3,3-dimethyl-1-oxobutan-2-yl)amino)-6-oxohexanoyl)-1,2,3,4-tetrahydroisoquinolin-7-yl)-1*H*-indazole-3-carboxamide (4b)

Compound **4b** was synthesized following a similar procedure to that of compound **4a**. ^1^H NMR (400 MHz, DMSO-*d*_6_) *δ* 13.95 (s, 1H), 10.35 (d, *J* = 8.7 Hz, 1H), 8.97 (d, *J* = 8.5 Hz, 1H), 8.56 (d, *J* = 5.4 Hz, 1H), 8.45 (s, 1H), 8.27 (d, *J* = 4.8 Hz, 1H), 8.22–8.12 (m, 1H), 7.88 (d, *J* = 9.5 Hz, 1H), 7.80 (d, *J* = 8.7 Hz, 1H), 7.73–7.6 (m, 3H), 7.54–7.47 (m, 1H), 7.46–7.30 (m, 4H), 7.15 (d, *J* = 8.3 Hz, 1H), 5.14 (d, *J* = 3.9 Hz, 1H), 4.66 (s, 1H), 4.60 (s, 1H), 4.55 (d, *J* = 9.4 Hz, 1H), 4.49–4.39 (m, 2H), 4.35 (s, 1H), 4.22 (dd, *J* = 16.1, 5.4 Hz, 1H), 3.73–3.59 (m, 4H), 2.83 (t, *J* = 5.9 Hz, 1H), 2.72 (t, *J* = 5.9 Hz, 1H), 2.47–2.25 (m, 5H), 2.34–2.24 (m, 1H), 2.16 (d, *J* = 12.2 Hz, 1H), 2.09–1.98 (m, 1H), 1.96–1.78 (m, 1H), 1.66–1.36 (m, 4H), 0.94 (s, 9H). ^13^C NMR (151 MHz, DMSO-*d*_6_) *δ* 172.49, 172.44, 171.47, 171.44, 170.19, 160.18 (d, *J*_*C-F*_ = 236.9 Hz), 159.40, 151.91, 148.18, 146.73 (d, *J*_*C-F*_ = 14.5 Hz), 142.13 (d, *J*_*C-F*_ = 4.1 Hz), 142.10, 142.06, 141.35, 139.97, 139.22, 137.36, 137.31, 134.32, 133.96, 131.64, 130.15, 130.10, 129.10, 128.17, 127.89, 123.68 (d, *J*_*C-F*_ = 28.1 Hz), 123.25 (d, *J*_*C-F*_ = 4.1 Hz), 122.52 (d, *J*_*C-F*_ = 3.3 Hz), 122.43, 119.28, 119.13, 118.42, 118.28, 111.86, 69.35, 59.17, 56.85, 56.79, 47.15, 44.16, 43.20, 42.12, 38.41, 35.70, 35.19, 32.66, 28.84, 26.86, 25.74, 16.41. HRMS (ESI) for C_50_H_54_O_6_N_9_FNaS [M+Na]^+^, calcd: 950.3794; found: 950.3770. HPLC purity: 98.7%, 6.02 min.

#### 4.1.17. 5-(2-fluoropyridin-3-yl)-*N*-(2-(8-(((*S*)-1-((2*S*,4*R*)-4-hydroxy-2-((4-(4-methylthiazol-5-yl)benzyl)carbamoyl)pyrrolidin-1-yl)-3,3-dimethyl-1-oxobutan-2-yl)amino)-8-oxooctanoyl)-1,2,3,4-tetrahydroisoquinolin-7-yl)-1*H*-indazole-3-carboxamide (4c)

Compound **4c** was synthesized by following a procedure similar to that used for **4a**. ^1^H NMR (400 MHz, DMSO-*d*_6_) *δ* 13.95 (s, 1H), 10.35 (d, *J* = 6.5 Hz, 1H), 8.97 (s, 1H), 8.56 (q, *J* = 5.0, 3.9 Hz, 1H), 8.45 (s, 1H), 8.27 (d, *J* = 4.6 Hz, 1H), 8.24–8.11 (m, 1H), 7.91–7.77 (m, 2H), 7.77–7.54 (m, 3H), 7.51 (t, *J* = 6.3 Hz, 1H), 7.40 (q, *J* = 8.2 Hz, 4H), 7.14 (dd, *J* = 8.6, 3.9 Hz, 1H), 5.13 (t, *J* = 3.4 Hz, 1H), 4.65 (s, 1H), 4.60 (s, 1H), 4.55 (dd, *J* = 9.4, 3.8 Hz, 1H), 4.48–4.39 (m, 2H), 4.35 (s, 1H), 4.22 (dd, *J* = 15.9, 5.4 Hz, 1H), 3.67 (d, *J* = 5.5 Hz, 4H), 2.82 (t, *J* = 5.8 Hz, 1H), 2.72 (t, *J* = 5.9 Hz, 1H), 2.44 (s, 3H), 2.42–2.33 (m, 2H), 2.30–2.20 (m, 1H), 2.17–2.07 (m, 1H), 2.07–1.97 (m, 1H), 1.95–1.84 (m, 1H), 1.59–1.40 (m, 4H), 1.35–1.06 (m, 4H), 0.93 (s, 9H). ^13^C NMR (151 MHz, DMSO-*d*_6_) *δ* 172.60, 172.44, 171.55, 170.20, 161.16, 160.18 (d, *J*_*C-F*_ = 236.8 Hz), 151.91, 148.18, 146.73 (d, *J*_*C-F*_ = 15.2 Hz), 142.13 (d, *J*_*C-F*_ = 4.7 Hz), 142.10, 141.35, 139.97, 139.22, 137.36, 137.29, 134.33, 133.99, 131.64, 130.14, 130.10, 129.10, 128.18, 127.89, 123.68 (d, *J*_*C-F*_ = 28.3 Hz), 123.25 (d, *J*_*C-F*_ = 4.1 Hz), 122.53 (d, *J*_*C-F*_ = 3.5 Hz), 122.48, 119.29, 119.13, 118.42, 118.28, 111.86, 69.34, 59.16, 56.83, 56.76, 47.20, 44.16, 43.21, 42.12, 38.41, 35.68, 35.33, 32.87, 29.01, 28.83, 26.84 (d, *J* = 3.0 Hz), 25.84, 25.14, 16.41. HRMS (ESI) for C_52_H_58_O_6_N_9_FNaS [M+Na]^+^, calcd: 978.4107; found: 978.4084. HPLC purity: 99.0%, 7.00 min.

#### 4.1.18. 5-(2-fluoropyridin-3-yl)-*N*-(2-(10-(((*S*)-1-((2*S*,4*R*)-4-hydroxy-2-((4-(4-methylthiazol-5-yl)benzyl)carbamoyl)pyrrolidin-1-yl)-3,3-dimethyl-1-oxobutan-2-yl)amino)-10-oxodecanoyl)-1,2,3,4-tetrahydroisoquinolin-7-yl)-1*H*-indazole-3-carboxamide (4d)

Compound **4d** was synthesized by following a similar procedure as that of **4a**. ^1^H NMR (400 MHz, DMSO-*d*_6_) *δ* 13.95 (s, 1H), 10.35 (d, *J* = 7.7 Hz, 1H), 8.97 (d, *J* = 1.7 Hz, 1H), 8.56 (t, *J* = 6.2 Hz, 1H), 8.45 (s, 1H), 8.26 (d, *J* = 4.7 Hz, 1H), 8.19 (t, *J* = 9.0 Hz, 1H), 7.92–7.57 (m, 5H), 7.54–7.45 (m, 1H), 7.40 (q, *J* = 8.0 Hz, 4H), 7.14 (dd, *J* = 8.8, 3.7 Hz, 1H), 5.17–5.11 (m, 1H), 4.65 (s, 1H), 4.59 (s, 1H), 4.54 (d, *J* = 9.4 Hz, 1H), 4.43 (t, *J* = 8.3 Hz, 2H), 4.35 (s, 1H), 4.22 (dd, *J* = 16.0, 5.5 Hz, 1H), 3.73–3.58 (m, 4H), 2.81 (d, *J* = 6.2 Hz, 1H), 2.72 (d, *J* = 6.0 Hz, 1H), 2.44 (s, 3H), 2.41–2.32 (m, 2H), 2.31–2.20 (m, 1H), 2.17–1.97 (m, 2H), 1.92 (d, *J* = 11.1 Hz, 1H), 1.60–1.40 (m, 4H), 1.33–1.08 (m, 8H), 0.93 (d, *J* = 3.9 Hz, 9H). ^13^C NMR (151 MHz, DMSO-*d*_6_) *δ* 172.63, 172.45, 171.58, 170.20, 161.16, 160.96, 159.40, 151.91, 148.18, 146.73 (d, *J*_*C-F*_ = 14.8 Hz), 142.12 (d, *J*_*C-F*_ = 4.5 Hz), 142.07, 141.35, 139.96, 139.21, 137.35, 137.28, 134.32, 134.01, 131.64, 130.15, 130.10, 129.10, 128.18, 127.89, 123.68 (d, *J*_*C-F*_ = 28.1 Hz), 123.25 (d, *J*_*C-F*_ = 3.8 Hz), 122.52 (d, *J*_*C-F*_ = 3.2 Hz), 122.42, 119.29, 119.13, 118.42, 118.28, 111.87, 69.34, 59.17, 56.82, 56.76, 47.22, 44.16, 43.21, 42.12, 38.41, 35.67, 35.34, 32.91, 29.33, 29.29, 29.18, 29.11, 26.84, 25.91, 25.24, 16.40. HRMS (ESI) for C_54_H_63_O_6_N_9_FS [M+Na]^+^, calcd: 984.4601; found: 984.4582. HPLC purity: 99.1%, 4.91 min.

#### 4.1.19. 5-(2-fluoropyridin-3-yl)-*N*-(2-(12-(((*S*)-1-((2*S*,4*R*)-4-hydroxy-2-((4-(4-methylthiazol-5-yl)benzyl)carbamoyl)pyrrolidin-1-yl)-3,3-dimethyl-1-oxobutan-2-yl)amino)-12-oxododecanoyl)-1,2,3,4-tetrahydroisoquinolin-7-yl)-1*H*-indazole-3-carboxamide (4e)

Compound **4e** was synthesized by following a similar procedure as that of **4a**. ^1^H NMR (400 MHz, DMSO-*d*_6_) *δ* 13.95 (s, 1H), 10.35 (d, *J* = 7.7 Hz, 1H), 8.98 (s, 1H), 8.56 (t, *J* = 6.2 Hz, 1H), 8.45 (s, 1H), 8.27 (d, *J* = 4.7 Hz, 1H), 8.19 (t, *J* = 9.1 Hz, 1H), 7.90–7.58 (m, 5H), 7.51 (t, *J* = 6.3 Hz, 1H), 7.40 (q, *J* = 8.0 Hz, 4H), 7.14 (dd, *J* = 8.6, 4.2 Hz, 1H), 5.13 (s, 1H), 4.65 (s, 1H), 4.59 (s, 1H), 4.54 (d, *J* = 9.3 Hz, 1H), 4.47–4.39 (m, 2H), 4.35 (s, 1H), 4.21 (dd, *J* = 16.0, 5.5 Hz, 1H), 3.75–3.59 (m, 4H), 2.81 (d, *J* = 6.1 Hz, 1H), 2.71 (t, *J* = 6.0 Hz, 1H), 2.44 (s, 3H), 2.41–2.32 (m, 2H), 2.30–2.18 (m, 1H), 2.16–1.98 (m, 2H), 1.97–1.83 (m, 1H), 1.59–1.38 (m, 4H), 1.35–1.10 (m, 12H), 0.93 (d, *J* = 3.5 Hz, 9H). ^13^C NMR (151 MHz, DMSO-*d*_6_) *δ* 172.59, 172.43, 171.57, 170.19, 161.15, 160.18 (d, *J*_*C-F*_ = 236.8 Hz), 151.91, 148.18, 146.73 (d, *J*_*C-F*_ = 15.0 Hz), 142.13 (d, *J*_*C-F*_ = 4.5 Hz), 142.08, 141.35, 139.97, 139.22, 137.36, 137.29, 134.33, 134.03, 131.64, 130.15, 130.10, 129.10, 128.18, 127.89, 123.68 (d, *J*_*C-F*_ = 27.9 Hz), 123.26 (d, *J*_*C-F*_ = 4.1 Hz), 122.52 (d, *J*_*C-F*_ = 3.8 Hz), 122.43, 119.28, 119.12, 118.42, 118.26, 111.86, 69.34, 59.16, 56.81, 56.75, 47.24, 44.15, 43.21, 42.11, 38.41, 35.68, 35.33, 32.91, 29.43, 29.39, 29.32, 29.23, 29.13, 28.83, 26.84, 25.90, 25.24, 16.41. HRMS (ESI) for C_56_H_67_O_6_N_9_FS [M+Na]^+^, calcd: 1012.4914; found: 1012.4889. HPLC purity: 99.1%, 5.94 min.

#### 4.1.20. *N*-(2-((2-(2,6-dioxopiperidin-3-yl)-1,3-dioxoisoindolin-4-yl)glycyl)-1,2,3,4-tetrahydroisoquinolin-7-yl)-5-(2-fluoropyridin-3-yl)-1-(tetrahydro-2*H*-pyran-2-yl)-1*H*-indazole-3-carboxamide (22a)

Compound **16** (183 mg, 0.39 mmol) was added to a solution of compound **21a** (129 mg, 0.39 mmol), HATU (180 mg, 46.00 mmol) and DIPEA (0.2 mL, 1.16 mmol) in DMF (5.0 mL). The reaction mixture was stirred at room temperature for 1 hour, resulting in the formation of precipitates upon the addition of water (15 mL). The precipitated solid was isolated by filtration and further purified by silica gel column chromatography to afford the title compound **22a** (190 mg, 62.5% yield). ^1^H NMR (400 MHz, DMSO-*d*_6_) δ 11.11 (s, 1H), 10.28 (d, *J* = 21.6 Hz, 1H), 8.46 (d, *J* = 7.5 Hz, 1H), 8.28 (s, 1H), 8.21 (t, *J* = 9.1 Hz, 1H), 8.05–7.93 (m, 1H), 7.81–7.67 (m, 2H), 7.66–7.57 (m, 1H), 7.55–7.47 (m, 1H), 7.26–6.97 (m, 5H), 6.06 (d, *J* = 9.9 Hz, 1H), 5.08 (dd, *J* = 12.9, 5.4 Hz, 1H), 4.73 (s, 2H), 4.32 (s, 2H), 4.04–3.94 (m, 1H), 3.88–3.73 (m, 3H), 3.05–2.75 (m, 5H), 2.69–2.60 (m, 2H), 2.16–1.95 (m, 4H), 1.82 (m, 1H).

#### 4.1.21. *N*-(2-((2-(2,6-dioxopiperidin-3-yl)-1,3-dioxoisoindolin-4-yl)glycyl)-1,2,3,4-tetrahydroisoquinolin-7-yl)-5-(2-fluoropyridin-3-yl)-1*H*-indazole-3-carboxamide (5a)

Trifluoroacetic acid (3.0 mL) was added to a solution of intermediate **22a** (100 mg, 128 μmol) and Et_3_SiH (40.6 μL, 256 μmol) in DCM (3.0 mL). The mixture was stirred at room temperature overnight. The solvent was evaporated under reduced pressure and purified by silica gel column chromatography to afford the title compound **5a** (73.3 mg, 82.2% yield). ^1^H NMR (400 MHz, DMSO-*d*_6_) *δ* 13.93 (s, 1H), 11.10 (s, 1H), 10.39 (d, *J* = 25.0 Hz, 1H), 8.56–8.37 (m, 1H), 8.27 (s, 1H), 8.20 (t, *J* = 9.1 Hz, 1H), 7.84–7.56 (m, 4H), 7.51 (s, 1H), 7.23–7.10 (m, 3H), 7.08 (d, *J* = 7.1 Hz, 1H), 5.08 (dd, *J* = 13.1, 5.6 Hz, 1H), 4.72 (d, *J* = 24.7 Hz, 2H), 4.31 (s, 2H), 3.90–3.56 (m, 2H), 2.96–2.83 (m, 2H), 2.82–2.73 (m, 1H), 2.58 (t, 2H), 2.15–1.93 (m, 1H). ^13^C NMR (151 MHz, DMSO-*d*_6_) *δ* 173.30, 170.57, 169.29, 167.84, 167.41, 167.34, 161.23, 161.18, 160.18 (d, *J*_*C-F*_ = 236.8 Hz), 146.74 (d, *J*_*C-F*_ = 15.1 Hz), 146.00, 145.9, 142.13 (d, *J*_*C-F*_ = 4.1 Hz), 142.10, 142.07, 141.36, 139.25, 139.20, 137.46, 136.62, 133.71, 133.33, 132.49, 130.08, 129.07, 128.17, 123.68 (d, *J*_*C-F*_ = 28.2 Hz), 123.28 (d, *J*_*C-F*_ = 4.4 Hz), 122.55 (d, *J*_*C-F*_ = 3.1 Hz), 122.43 (d, *J*_*C-F*_ = 3.3 Hz), 119.34, 119.30, 118.76, 118.44, 111.86, 111.29, 110.03, 49.06, 45.77, 44.51, 42.08, 31.48, 28.43, 22.63. HRMS (ESI) for C_37_H_28_O_6_N_8_F [M+H]^+^, calcd: 699.2110; found: 699.2126. HPLC purity: 98.6%, 10.28 min.

#### 4.1.22. *N*-(2-(4-((2-(2,6-dioxopiperidin-3-yl)-1,3-dioxoisoindolin-4-yl)amino)-butanoyl)-1,2,3,4-tetrahydroisoquinolin-7-yl)-5-(2-fluoropyridin-3-yl)-1*H*-indazole-3-carboxamide (5b)

Compound **5b** was synthesized following a procedure similar to that used for **6a**.^1^H NMR (400 MHz, DMSO-*d*_6_) *δ* 13.94 (s, 1H), 11.45–10.75 (m, 1H), 10.61–9.97 (m, 1H), 8.46 (s, 1H), 8.27 (q, *J* = 1.8 Hz, 1H), 8.19 (ddd, *J* = 9.9, 7.4, 1.9 Hz, 1H), 7.90–7.74 (m, 2H), 7.74–7.59 (m, 2H), 7.56 (t, *J* = 7.8 Hz, 1H), 7.53–7.47 (m, 1H), 7.16 (dd, *J* = 15.2, 8.1 Hz, 2H), 7.00 (t, *J* = 7.1 Hz, 1H), 6.68 (t, *J* = 6.1 Hz, 1H), 5.05 (dd, *J* = 13.0, 5.5, 2.8 Hz, 1H), 3.79–3.52 (m, 2H), 2.95–2.84 (m, 1H), 2.80 (t, *J* = 5.9 Hz, 1H), 2.73 (t, *J* = 6.0 Hz,1H), 2.64–2.52 (m, 3H), 2.50–2.43 (m, 1H), 2.10–1.94 (m, 1H), 1.90–1.77 (m, 2H), 1.26–1.17 (m, 2H). ^13^C NMR (151 MHz, DMSO-*d*_6_) *δ* 173.30, 171.11, 170.59, 169.29, 167.79, 161.16, 160.18 (d, *J*_*C-F*_ = 236.8 Hz), 146.85, 146.83, 146.73 (d, *J*_*C-F*_ = 14.9 Hz), 142.12 (d, *J*_*C-F*_ = 4.4 Hz), 142.07, 141.36, 139.23, 137.35 (d, *J*_*C-F*_ = 8.6 Hz), 136.65, 134.27, 133.87, 132.70, 130.15, 129.02, 128.17, 123.68 (d, *J*_*C-F*_ = 28.2 Hz), 123.26 (d, *J*_C-F_ = 3.2 Hz), 123.22, 122.54 (d, *J*_*C-F*_ = 3.0 Hz), 122.43 (d, *J*_*C-F*_ = 3.2 Hz), 119.29, 119.13, 118.41, 117.73, 111.89, 111.86 110.83,110.81, 109.56, 49.00, 44.25, 43.13, 42.09, 31.46, 30.03, 28.69, 24.63, 22.64. HRMS (ESI) for C_39_H_32_O_6_N_8_F [M+H]^+^, calcd: 727.2441; found: 727.2423. HPLC purity: 97.9%, 10.63 min.

#### 4.1.23. *N*-(2-(6-((2-(2,6-dioxopiperidin-3-yl)-1,3-dioxoisoindolin-4-yl)amino)- hexanoyl)-1,2,3,4-tetrahydroisoquinolin-7-yl)-5-(2-fluoropyridin-3-yl)-1*H*-indazole-3-carboxamide (5c)

Compound **5c** was synthesized following a procedure similar to that used for **5a**. ^1^H NMR (400 MHz, DMSO-*d*_6_) *δ* 13.94 (s, 1H), 11.45–10.75 (m, 1H), 10.61–9.97 (m, 1H), 8.46 (s, 1H), 8.27 (q, *J* = 1.8 Hz, 1H), 8.19 (ddd, *J* = 9.9, 7.4, 1.9 Hz, 1H), 7.90–7.74 (m, 2H), 7.74–7.59 (m, 2H), 7.56 (t, *J* = 7.8 Hz, 1H), 7.53–7.47 (m, 1H), 7.16 (dd, *J* = 15.2, 8.1 Hz, 2H), 7.00 (t, *J* = 7.1 Hz, 1H), 6.68 (t, *J* = 6.1 Hz, 1H), 5.05 (dd, *J* = 13.0, 5.5, 2.8 Hz, 1H), 3.79–3.52 (m, 2H), 2.95–2.84 (m, 1H), 2.80 (t, *J* = 5.9 Hz, 1H), 2.73 (t, *J* = 6.0 Hz, 1H), 2.64–2.52 (m, 3H), 2.50–2.43 (m, 1H), 2.10–1.94 (m, 1H), 1.90–1.77 (m, 2H), 1.26–1.17 (m, 2H). ^13^C NMR (151 MHz, DMSO-*d*_6_) *δ* 173.29, 171.45, 170.58, 169.41, 167.78, 161.16, 160.18 (d, *J*_*C-F*_ = 236.8 Hz), 146.89, 146.73 (d, *J*_*C-F*_ = 14.9 Hz), 142.13 (d, *J*_*C-F*_ = 4.7 Hz), 142.07, 141.36, 139.22, 137.36, 137.29, 136.74, 136.72, 134.32, 134.01, 132.67, 130.16, 129.03, 128.17, 123.68 (d, *J* = 28.1 Hz), 123.25 (d, *J*_*C-F*_ = 3.9 Hz), 122.53 (d, *J*_*C-F*_ = 3.1 Hz), 122.43, 119.12, 118.41, 117.65, 111.87, 110.83, 109.46, 49.01, 44.17, 43.19, 42.25, 32.80, 31.45, 29.06, 28.83, 26.61, 24.96, 22.63. HRMS (ESI) for C_41_H_37_O_6_N_8_FNa [M+Na]^+^, calcd: 779.2712; found: 779.2698. HPLC purity: 98.7%, 6.15 min.

#### 4.1.24. *N*-(2-(8-((2-(2,6-dioxopiperidin-3-yl)-1,3-dioxoisoindolin-4-yl)amino)-octanoyl)-1,2,3,4-tetrahydroisoquinolin-7-yl)-5-(2-fluoropyridin-3-yl)-1*H*-indazole-3-carboxamide (5d)

Compound **5d** was synthesized following a similar procedure as that of **5a**. ^1^H NMR (400 MHz, DMSO-*d*_6_) *δ* 13.93 (s, 1H), 11.08 (s, 1H), 10.32 (d, *J* = 5.9 Hz, 1H), 8.45 (d, *J* = 4.8 Hz, 1H), 8.26 (s, 1H), 8.23–8.08 (m, 1H), 7.92–7.53 (m, 5H), 7.53–7.47 (m, 1H), 7.14 (t, *J* = 5.9 Hz, 1H), 7.10–7.04 (m, 1H), 7.00 (dd, *J* = 10.6, 6.4 Hz, 1H), 6.51 (d, *J* = 7.2 Hz, 1H), 5.04 (dd, *J* = 11.8, 5.6 Hz, 1H), 4.62 (dd, *J* = 21.4, 4.3 Hz, 2H), 3.67 (t, *J* = 5.5 Hz, 2H), 3.26 (t, *J* = 6.5 Hz, 2H), 2.93–2.77 (m, 2H), 2.71 (d, *J* = 5.9 Hz, 1H), 2.63–2.52 (m, 2H), 2.44–2.33 (m, 2H), 2.08–1.93 (m, 1H), 1.63–1.45 (m, 4H), 1.39–1.26 (m, 6H). ^13^C NMR (151 MHz, DMSO-*d*_6_) *δ* 173.29, 171.54, 170.58, 169.42 (d, *J* = 3.0 Hz), 167.78, 161.16, 160.18 (d, *J*_*C-F*_ = 236.8 Hz), 146.90, 146.87, 146.73 (d, *J*_*C-F*_ = 14.8 Hz), 142.13 (d, *J*_*C-F*_ = 3.9 Hz), 142.10, 142.07, 141.35, 139.22, 137.37, 137.30, 136.74, 136.70, 134.34, 134.04, 132.66, 130.14, 129.03, 128.16, 123.69 (d, *J*_*C-F*_ = 28.3 Hz), 123.25 (d, *J*_*C-F*_ = 3.9 Hz), 122.53 (d, *J*_*C-F*_ = 3.5 Hz), 122.43, 119.12, 118.42, 117.65, 111.87, 110.83, 109.47, 49.01, 44.15, 43.20, 42.29, 32.86, 31.45, 29.24, 29.12, 29.09, 28.83, 26.72, 25.16, 22.62. HRMS (ESI) for C_43_H_41_O_6_N_8_FNa [M+Na]^+^, calcd: 807.3025; found: 807.3004. HPLC purity: 98.4%, 8.02 min.

#### 4.1.25. *N*-(2-(10-((2-(2,6-dioxopiperidin-3-yl)-1,3-dioxoisoindolin-4-yl)amino)-decanoyl)-1,2,3,4-tetrahydroisoquinolin-7-yl)-5-(2-fluoropyridin-3-yl)-1*H*-indazole-3-carboxamide (5e)

Compound **5e** was synthesized by following a similar procedure as that of **5a**. ^1^H NMR (400 MHz, DMSO-*d*_6_) *δ* 13.92 (s, 1H), 11.49–10.75 (m, 1H), 10.34 (d, *J* = 8.0 Hz, 1H), 8.46 (s, 1H), 8.26 (d, *J* = 4.8 Hz, 1H), 8.18 (dd, *J* = 11.0, 6.5 Hz, 1H), 7.94–7.53 (m, 5H), 7.50 (t, *J* = 6.5 Hz, 1H), 7.14 (d, 1H), 7.08–7.03 (m, 1H), 7.02–6.95 (m, 1H), 6.48 (dt, *J* = 11.8, 6.0 Hz, 1H), 5.05 (d, 1H), 4.62 (d, *J* = 20.5 Hz, 2H), 3.73–3.56 (m, 2H), 3.31–3.15 (m, 2H), 2.96–2.75 (m, 2H), 2.71 (t, *J* = 6.0 Hz, 1H), 2.63–2.53 (m, 2H), 2.38 (dt, *J* = 11.4, 7.4 Hz, 2H), 2.12–1.94 (m, 1H), 1.62–1.45 (m, 4H), 1.40–1.08 (m, 10H). ^13^C NMR (151 MHz, DMSO-*d*_6_) *δ* 173.28, 171.54, 170.57, 169.42, 167.78, 161.15, 160.18 (d, *J*_*C-F*_ = 236.8 Hz), 146.88, 146.71 (d, *J*_*C-F*_ = 14.9 Hz), 142.10 (d, *J*_*C-F*_ = 4.5 Hz), 142.06, 142.03, 141.36, 139.23, 137.38, 137.32, 136.71, 136.67, 134.33, 134.03, 132.64, 130.11, 129.02, 128.15, 123.68 (d, *J*_*C-F*_ = 28.3 Hz), 123.23 (d, *J*_*C-F*_ = 4.1 Hz), 122.53 (d, *J*_*C-F*_ = 3.1 Hz), 122.44, 119.26, 119.10, 118.40, 117.60, 111.85, 110.82, 109.47, 49.01, 44.16, 43.20, 42.29, 32.90, 31.46, 29.39, 29.33, 29.27, 29.19, 29.13, 28.83, 26.78, 25.22, 22.64. HRMS (ESI) for C_45_H_45_O_6_N_8_FNa [M+Na]^+^, calcd: 835.3338; found:835.3317. HPLC purity: 96.1%, 13.35 min.

#### 4.1.26. *N*-(2-(12-((2-(2,6-dioxopiperidin-3-yl)-1,3-dioxoisoindolin-4-yl)amino)-dodecanoyl)-1,2,3,4-tetrahydroisoquinolin-7-yl)-5-(2-fluoropyridin-3-yl)-1*H*-indazole-3-carboxamide (5f)

Compound **5f** was synthesized following a procedure similar to that used for **5a**. ^1^H NMR (400 MHz, DMSO-*d*_6_) *δ* 13.92 (s, 1H), 11.09 (s, 1H), 10.32 (d, *J* = 7.2 Hz, 1H), 8.46 (s, 1H), 8.33–8.05 (m, 2H), 7.92–7.58 (m, 4H), 7.58–7.43 (m, 2H), 7.13 (d, *J* = 8.4 Hz, 1H), 7.09–6.94 (m, 2H), 6.55–6.39 (m, 1H), 5.05 (dd, *J* = 12.8, 5.5 Hz, 1H), 4.61 (d, *J* = 19.3 Hz, 2H), 3.66 (s, 2H), 3.23 (dd, *J* = 13.5, 6.8 Hz, 2H), 2.97–2.75 (m, 2H), 2.71 (d, *J* = 6.4 Hz, 1H), 2.63–2.45 (m, 2H), 2.43–2.27 (m, 2H), 2.12–1.94 (m, 1H), 1.58–1.43 (m, 4H), 1.34–1.14 (m, 12H).^13^C NMR (151 MHz, DMSO-*d*_6_) *δ* 173.28, 171.53, 170.57, 169.42, 167.77, 161.15, 160.18 (d, *J*_*C-F*_ = 236.8 Hz), 146.87, 146.70 (d, *J* = 14.8 Hz), 142.07 (d, *J*_*C-F*_ = 4.5 Hz), 142.04, 142.02, 141.36, 139.23, 137.39, 137.33, 136.69, 136.67, 134.32, 134.02, 132.63, 130.09, 129.01, 128.14, 123.68 (d, *J*_*C-F*_ = 28.3 Hz), 123.22 (d, *J*_*C-F*_ = 4.4 Hz), 122.55 (d, *J*_*C-F*_ = 3.1 Hz), 122.44, 119.09, 118.38, 117.57, 111.84, 110.81, 109.46, 49.01, 44.16, 43.20, 42.30, 33.26, 32.90, 31.46, 29.47, 29.43, 29.30, 29.23, 29.13, 28.84, 27.98, 26.78, 25.23, 22.64. HRMS (ESI) for C_47_H_49_O_6_N_8_FNa [M+Na]^+^, calcd: 863.3651; found: 863.3636. HPLC purity: 96.1%, 6.07 min.

#### 4.1.27. *N*-(2-((2-(2,6-dioxopiperidin-3-yl)-1,3-dioxoisoindolin-5-yl)glycyl)-1,2,3,4-tetrahydroisoquinolin-7-yl)-5-(2-fluoropyridin-3-yl)-1*H*-indazole-3-carboxamide (5g).

Compound **5g** was synthesized following a procedure similar to that used for **5a**. ^1^H NMR (400 MHz, DMSO-*d*_6_) *δ* 13.92 (s, 1H), 11.06 (s, 1H), 10.37 (d, *J* = 21.8 Hz, 1H), 8.47 (d, *J* = 9.5 Hz, 1H), 8.32–8.12 (m, 2H), 8.03–7.55 (m, 5H), 7.51 (s, 1H), 7.24–7.10 (m, 3H), 7.09–6.98 (m, 1H), 5.05 (dd, *J* = 13.1, 5.5 Hz, 1H), 4.73 (d, *J* = 43.4 Hz, 2H), 4.26 (s, 2H), 3.98–3.57 (m, 2H), 2.96–2.82 (m, 2H), 2.79 (d, *J* = 6.0 Hz, 1H), 2.63–2.52 (m, 2H), 2.10–1.68 (m, 1H). ^13^C NMR (151 MHz, DMSO-*d*_6_) *δ* 173.31, 170.66, 168.24, 167.70, 167.65, 161.21, 161.18, 160.18 (d, *J*_*C-F*_ = 236.8 Hz), 154.52, 146.73 (d, *J*_*C-F*_ = 15.4 Hz), 142.12 (d, *J*_*C-F*_ = 4.5 Hz), 142.10, 142.07, 141.36, 139.20, 137.42, 134.49, 133.82, 130.14, 129.14, 129.06, 128.20, 125.25, 123.66 (d, *J*_*C-F*_ = 28.7 Hz), 123.26 (d, *J*_*C-F*_ = 4.5 Hz), 122.54 (d, *J*_*C-F*_ = 3.0 Hz), 122.44, 119.28, 118.44, 118.35, 117.12 (d, *J*_*C-F*_ = 3.6 Hz), 111.87, 55.39, 49.12, 44.94, 44.48, 42.30, 31.47, 28.56. HRMS (ESI) for C_37_H_28_O_6_N_8_F [M+H]^+^, calcd: 699.2110; found: 699.2127. HPLC purity: 98.6%, 10.28 min.

#### 4.1.28. *N*-(2-(4-((2-(2,6-dioxopiperidin-3-yl)-1,3-dioxoisoindolin-5-yl)amino)-butanoyl)-1,2,3,4-tetrahydroisoquinolin-7-yl)-5-(2-fluoropyridin-3-yl)-1*H*-indazole-3-carboxamide (5h)

Compound **5h** was synthesized following a procedure similar to that used for **5a**. ^1^H NMR (400 MHz, DMSO-*d*_6_) *δ* 13.94 (s, 1H), 11.05 (s, 1H), 10.35 (d, *J* = 7.9 Hz, 1H), 8.45 (s, 1H), 8.27 (d, *J* = 4.8 Hz, 1H), 8.20 (t, *J* = 8.9 Hz, 1H), 7.90–7.60 (m, 4H), 7.60–7.42 (m, 2H), 7.26–7.07 (m, 2H), 7.01–6.93 (m, 1H), 6.86 (t, *J* = 7.4 Hz, 1H), 5.03 (dd, *J* = 13.1, 4.7 Hz, 1H), 4.65 (d, *J* = 17.7 Hz, 2H), 3.79–3.59 (m, 2H), 3.27–3.11 (m, 2H), 2.97–2.79 (m, 2H), 2.75 (d, *J* = 6.1 Hz, 1H), 2.61–2.52 (m, 3H), 2.05–1.93 (m, 2H), 1.90–1.77 (m, 2H). ^13^C NMR (151 MHz, DMSO-*d*_6_) δ 173.30, 171.12, 170.66, 168.16, 167.62, 161.16, 160.18 (d, *J*_*C-F*_ = 236.8 Hz), 154.90, 154.87, 146.73 (d, *J*_*C-F*_ = 15.4 Hz), 142.14 (d, *J*_*C-F*_ = 4.5 Hz), 142.09, 141.36, 139.22, 137.38, 137.33, 134.69, 134.29, 133.86, 132.17, 132.00, 130.37, 130.17, 130.12, 129.18, 129.14, 129.03, 128.19, 125.59, 123.68 (d, *J*_*C-F*_ = 28.7 Hz), 123.26 (d, *J*_*C-F*_ = 3.0 Hz), 122.53 (d, *J*_*C-F*_ = 3.1 Hz), 122.43, 119.29, 119.16, 118.43, 118.25, 116.36, 111.88, 49.09, 43.13, 42.53, 40.52, 31.46, 30.47, 28.7, 24.95, 22.71. HRMS (ESI) for C_39_H_33_O_6_N_8_FNa [M+Na]^+^, calcd:751.2399; found: 751.2393. HPLC purity: 100%, 8.20 min.

#### 4.1.29. *N*-(2-(6-((2-(2,6-dioxopiperidin-3-yl)-1,3-dioxoisoindolin-5-yl)amino)-hexanoyl)-1,2,3,4-tetrahydroisoquinolin-7-yl)-5-(2-fluoropyridin-3-yl)-1*H*-indazole-3-carboxamide (5i)

Compound **5i** was synthesized following a procedure similar to that used for **5a**. ^1^H NMR (400 MHz, DMSO-*d*_6_) *δ* 13.94 (s, 1H), 11.06 (s, 1H), 10.35 (d, *J* = 8.8 Hz, 1H), 8.45 (s, 1H), 8.30–8.24 (m, 1H), 8.24–8.15 (m, 1H), 7.92–7.58 (m, 4H), 7.58–7.47 (m, 2H), 7.18–7.05 (m, 2H), 6.94 (t, *J* = 2.8 Hz, 1H), 6.87–6.80 (m, 1H), 5.02 (dd, 1H), 4.63 (d, *J* = 24.1 Hz, 2H), 3.76–3.56 (m, 2H), 3.20–3.07 (m, 2H), 2.93–2.79 (m, 2H), 2.72 (t, *J* = 6.0 Hz, 1H), 2.62–2.52 (m, 2H), 2.48–2.35 (m, 2H), 2.00 (d, *J* = 12.6 Hz, 1H), 1.70–1.53 (m, 4H), 1.49–1.35 (m, 2H). ^13^C NMR (151 MHz, DMSO-*d*_6_) *δ* 173.30, 171.46, 170.67, 168.18, 167.63, 161.16, 160.18 (d, *J*_*C-F*_ = 236.8 Hz), 154.93, 146.73 (d, *J*_*C-F*_ = 15.5 Hz), 142.13 (d, *J*_*C-F*_ = 5.6 Hz), 142.11, 142.07, 141.35, 139.22, 137.36, 137.30, 134.32, 134.00, 130.15, 129.16, 129.04, 128.19, 128.17, 125.57, 123.68 (d, *J*_*C-F*_ = 28.7 Hz), 123.25 (d, *J*_*C-F*_ = 5.0 Hz), 122.53 (d, *J*_*C-F*_ = 3.1 Hz), 122.43, 119.13, 118.42, 118.27, 116.23, 111.86, 55.39, 49.09, 44.18, 43.22, 42.91, 32.85, 31.46, 28.82, 28.60, 26.81, 24.95, 22.72. HRMS (ESI) for C_41_H_37_O_6_N_8_FNa [M+Na]^+^, calcd: 779..2712; found: 779.2694. HPLC purity: 98.8%, 4.72 min.

#### 4.1.30. *N*-(2-(8-((2-(2,6-dioxopiperidin-3-yl)-1,3-dioxoisoindolin-5-yl)amino)-octanoyl)-1,2,3,4-tetrahydroisoquinolin-7-yl)-5-(2-fluoropyridin-3-yl)-1*H*-indazole-3-carboxamide (5j)

Compound **5j** was synthesized following a procedure similar to that used for **5a**. ^1^H NMR (400 MHz, DMSO-*d*_6_) *δ* 13.76 (s, 1H), 11.05 (s, 1H), 10.34 (d, *J* = 8.5 Hz, 1H), 8.46 (s, 1H), 8.26 (d, *J* = 4.8 Hz, 1H), 8.23–8.13 (m, 1H), 7.91–7.58 (m, 4H), 7.58–7.41 (m, 2H), 7.14 (dd, *J* = 8.5, 2.5 Hz, 1H), 7.11–7.03 (m, 1H), 6.93 (dd, *J* = 7.9, 2.1 Hz, 1H), 6.82 (ddd, *J* = 10.6, 8.3, 2.1 Hz, 1H), 5.03 (dd, *J* = 13.1, 5.5, 2.7 Hz, 1H), 4.63 (d, *J* = 22.4 Hz, 2H), 3.67 (d, *J* = 6.0 Hz, 2H), 3.23–3.03 (m, 2H), 2.95–2.77 (m, 2H), 2.72 (t, *J* = 5.8 Hz, 1H), 2.63–2.52 (m, 2H), 2.45–2.34 (m, 2H), 2.06–1.91 (m, 1H), 1.63–1.48 (m, 4H), 1.41–1.27 (m, 6H). ^13^C NMR (151 MHz, DMSO-*d*_6_) *δ* 173.30, 171.54, 170.67, 168.19, 167.63, 161.16, 160.18 (d, *J*_*C-F*_ = 236.8 Hz), 154.93, 146.72 (d, *J*_*C-F*_ = 15.5 Hz), 142.12 (d, *J*_*C-F*_ = 4.5 Hz), 142.08, 142.05, 141.36, 139.21, 137.36, 137.30, 134.33, 134.02, 130.14, 129.29, 129.03, 128.16, 128.13, 125.55, 123.68 (d, *J*_*C-F*_ = 28.7 Hz), 123.24 (d, *J*_*C-F*_ = 5.0 Hz), 122.53 (d, *J*_*C-F*_ = 3.0 Hz), 122.43, 119.13, 118.42, 118.24, 116.23, 116.20, 111.87, 55.39, 49.09, 44.17, 43.21, 42.96, 32.87, 31.46, 29.27, 29.14, 28.83, 28.68, 26.93, 25.18, 22.72. HRMS (ESI) for C_43_H_41_O_6_N_8_FNa [M+Na]^+^, calcd: 807.3025; found: 807.3010. HPLC purity: 98.7%, 6.15 min.

#### 4.1.31. *N*-(2-(10-((2-(2,6-dioxopiperidin-3-yl)-1,3-dioxoisoindolin-5-yl)amino)-decanoyl)-1,2,3,4-tetrahydroisoquinolin-7-yl)-5-(2-fluoropyridin-3-yl)-1*H*-indazole-3-carboxamide (5k)

Compound **5k** was synthesized following a procedure similar to that used for **5a**. ^1^H NMR (400 MHz, DMSO-*d*_6_) *δ* 13.97 (s, 1H), 11.06 (s, 1H), 10.36 (d, *J* = 8.7 Hz, 1H), 8.45 (s, 1H), 8.27 (d, *J* = 4.7 Hz, 1H), 8.23–8.13 (m, 1H), 7.94–7.58 (m, 4H), 7.57–7.43 (m, 2H), 7.20–7.00 (m, 2H), 6.93 (d, *J* = 7.9 Hz, 1H), 6.82 (t, *J* = 10.0 Hz, 1H), 5.03 (dd, *J* = 13.0, 5.3 Hz, 1H), 4.62 (d, *J* = 23.3 Hz, 2H), 3.66 (d, *J* = 7.0 Hz, 2H), 3.18–3.00 (m, 2H), 2.85 (d, *J* = 21.0 Hz, 2H), 2.74–2.67 (m, 1H), 2.62–2.53 (m, 2H), 2.44–2.33 (m, 2H), 2.05–1.88 (m, 1H), 1.61–1.45 (m, 4H), 1.37–1.24 (m, 10H). ^13^C NMR (151 MHz, DMSO-*d*_6_) *δ* 173.29, 171.54, 170.66, 168.18, 167.62, 161.15, 160.18 (d, *J*_*C-F*_ = 236.8 Hz), 154.93, 146.73 (d, *J*_*C-F*_ = 15.6 Hz), 142.13 (d, *J*_*C-F*_ = 4.5 Hz), 142.07, 142.09, 142.06, 141.35, 139.21, 137.37, 137.31, 134.33, 134.03, 130.13, 129.13, 129.03, 128.19, 128.16, 125.54, 123.68 (d, *J*_*C-F*_ = 28.7 Hz), 123.25 (d, *J*_*C-F*_ = 4.7 Hz), 122.52 (d, *J*_*C-F*_ = 3.1 Hz), 122.43, 119.12, 118.41, 118.22, 116.22, 111.87, 49.08, 44.17, 43.22, 42.95, 32.89, 31.46, 30.47, 29.42, 29.34, 29.24, 28.70, 28.83, 27.00, 25.23, 22.71. HRMS (ESI) for C_45_H_46_O_6_N_8_F [M+H]^+^, calcd: 813.3519; found: 813.3499. HPLC purity: 96.0%, 9.76 min.

#### 4.1.32. *N*-(2-(12-((2-(2,6-dioxopiperidin-3-yl)-1,3-dioxoisoindolin-5-yl)amino)-dodecanoyl)-1,2,3,4-tetrahydroisoquinolin-7-yl)-5-(2-fluoropyridin-3-yl)-1*H*-indazole-3-carboxamide (5l)

Compound **5l** was synthesized following a procedure similar to that used for **5a**. ^1^H NMR (400 MHz, DMSO-*d*_6_) *δ* 13.92 (s, 1H), 11.51–10.86 (m, 1H), 10.34 (d, *J* = 7.9 Hz, 1H), 8.46 (d, *J* = 3.8 Hz, 1H), 8.26 (d, *J* = 4.7 Hz, 1H), 8.18 (t, *J* = 9.1 Hz, 1H), 7.92–7.58 (m, 4H), 7.58–7.43 (m, 2H), 7.14 (dd, *J* = 8.6, 3.6 Hz, 1H), 7.09–7.02 (m, 1H), 6.93 (d, *J* = 5.2 Hz, 1H), 6.82 (t, *J* = 7.9 Hz, 1H), 5.03 (dd, *J* = 12.9, 5.4 Hz, 1H), 4.62 (d, *J* = 21.6 Hz, 2H), 3.67 (q, *J* = 5.5 Hz, 2H), 3.19–3.04 (m, 2H), 2.95–2.77 (m, 2H), 2.72 (t, *J* = 6.0 Hz, 1H), 2.63–2.52 (m, 2H), 2.38 (q, *J* = 8.3 Hz, 2H), 2.06–1.93 (m, 1H), 1.67–1.41 (m, 4H), 1.40–1.08 (m, 14H). ^13^C NMR (151 MHz, DMSO-*d*_6_) *δ* 173.29, 171.55, 170.66, 168.18, 167.62, 161.15, 160.18 (d, *J*_*C-F*_ = 236.8 Hz), 154.93, 146.72 (d, *J*_*C-F*_ = 15.7 Hz), 142.12 (d, *J*_*C-F*_ = 4.6 Hz), 142.08, 142.06, 141.35, 139.22, 137.34 (d, *J*_*C-F*_ = 9.5 Hz), 134.33, 134.03, 130.13, 129.16, 129.03, 128.19, 128.16, 125.55, 123.67 (d, *J*_*C-F*_ = 28.7 Hz), 123.24 (d, *J*_*C-F*_ = 5.0 Hz), 122.54 (d, *J*_*C-F*_ = 3.0 Hz), 122.42, 119.11, 118.40, 118.21, 116.21, 111.85, 55.39, 49.08, 44.16, 43.21, 42.95, 33.25, 32.89, 31.45, 29.50, 29.44, 29.39, 29.36, 29.27, 29.22, 28.82, 28.68, 26.99, 25.23. HRMS (ESI) for C_47_H_49_O_6_N_8_FNa [M+Na]^+^, calcd: 863.3651; found: 863.3632.purity: 96.1%, 6.83 min.

#### 4.1.33. *Tert*-butyl 10-((2-(1-methyl-2,6-dioxopiperidin-3-yl)-1,3-dioxoisoindolin-5-yl)amino) decanoate (24)

Methyl iodide (0.1 μL, 0.6 mmol) was added to a solution of compound **23** (160 mg, 0.32 mmol) and K_2_CO_3_ in DMF (3.0 mL). The reaction mixture was heated overnight at 50 °C and the solvent was evaporated under reduced pressure. The residue was purified by silica gel column chromatography to afford compound **24** (123 mg, 75% yield). ^1^H NMR (400 MHz, CDCl_3_) δ 7.67–7.57 (m, 1H), 7.00–6.93 (m, 1H), 6.79–6.68 (m, 1H), 4.95 (dd, 1H), 3.23 (s, 3H), 3.01–2.92 (m, 1H), 2.89–2.69 (m, 3H), 2.29–1.99 (m, 4H), 1.74–1.50 (m, 9H), 1.46 (s, 9H), 1.43–1.34 (m, 5H).

#### 4.1.34. 5-(2-fluoropyridin-3-yl)-*N*-(2-(10-((2-(1-methyl-2,6-dioxopiperidin-3-yl)-1,3-dioxoisoindolin-5-yl)amino)decanoyl)-1,2,3,4-tetrahydroisoquinolin-7-yl)-1-(tetrahydro-2*H*-pyran-2-yl)-1*H*-indazole-3-carboxamide (25)

Trifluoroacetic acid (3.0 mL) was added to a solution of compound **24** (123 mg, 0.24 mmol) in DCM (3.0 mL). The mixture was stirred at room temperature for one hour. The solvent was evaporated under reduced pressure and the residue was used in the next step without purification. The above freshly prepared carboxylic acid was added to a solution of compound **16** (129 mg, 0.27 mmol), HATU (117.0 mg, 30.00 mmol) and DIPEA (0.2 mL, 1.16 mmol) in DMF (5.0 mL). The reaction mixture was stirred at room temperature for one hour. Precipitates formed upon the addition of water (15.0 mL) to the mixture. The resulting precipitated solids were collected by filtration and purified by silica gel column chromatography to afford the title compound **25** (165 mg, 76% yield). ^1^H NMR (400 MHz, CDCl_3_) δ 8.86 (d, *J* = 12.2 Hz, 1H), 8.63 (s, 1H), 8.24 (d, *J* = 4.8 Hz, 1H), 8.06–7.90 (m, 2H), 7.76 (s, 2H), 7.67–7.48 (m, 2H), 7.32 (t, *J* = 6.0 Hz, 1H), 7.18 (d, *J* = 8.4 Hz, 1H), 6.97 (d, *J* = 10.6 Hz, 1H), 6.75 (t, *J* = 9.2 Hz, 1H), 5.85 (dd, *J* = 9.6, 2.5 Hz, 1H), 4.94 (dd, *J* = 12.2, 5.3 Hz, 1H), 4.84–4.58 (m, 2H), 4.21– 3.99 (m, 1H), 3.93–3.76 (m, 2H), 3.76–3.66 (m, 1H), 3.22 (s, 3H), 3.03–2.58 (m, 6H), 2.44 (t, *J* = 7.6 Hz, 2H), 2.29–2.01 (m, 4H), 1.90–1.63 (m, 8H), 1.45–1.23 (m, 10H).

#### 4.1.35. 5-(2-fluoropyridin-3-yl)-*N*-(2-(10-((2-(1-methyl-2,6-dioxopiperidin-3-yl)-1,3-dioxoisoindolin-5-yl)amino)decanoyl)-1,2,3,4-tetrahydroisoquinolin-7-yl)-1*H*-indazole-3-carboxamide (5m)

Compound **5m** was synthesized following a procedure similar to that used for **5a**. ^1^H NMR (400 MHz, DMSO-*d*_6_) *δ* 13.95 (s, 1H), 10.34 (d, *J* = 8.0 Hz, 1H), 8.45 (s, 1H), 8.26 (d, *J* = 4.8 Hz, 1H), 8.18 (t, *J* = 9.0 Hz, 1H), 7.90–7.57 (m, 4H), 7.57–7.46 (m, 2H), 7.18–7.02 (m, 2H), 6.93 (d, *J* = 7.8 Hz, 1H), 6.82 (t, *J* = 9.7 Hz, 1H), 5.09 (dd, *J* = 13.0, 5.4 Hz, 1H), 4.62 (d, *J* = 21.7 Hz, 2H), 3.66 (t, *J* = 5.8 Hz, 2H), 3.18–3.06 (m, 2H), 3.00 (s, 3H), 2.81 (d, *J* = 5.8 Hz, 1H), 2.78–2.66 (m, 4H), 2.44–2.28 (m, 2H), 2.05–1.93 (m, 1H), 1.60–1.44 (m, 4H), 1.40–1.15 (m, 10H). ^13^C NMR (151 MHz, DMSO-*d*_6_) *δ* 172.29, 171.57, 170.40, 168.17, 167.61, 161.16, 160.18 (d, *J*_*C-F*_ = 236.8 Hz), 154.95, 146.72 (d, *J*_*C-F*_ = 15.8 Hz), 142.11 (d, *J*_*C-F*_ = 4.5 Hz), 142.07, 142.04, 141.36, 139.19, 137.36, 137.29, 134.33, 134.03, 130.13, 129.17, 129.03, 128.18, 128.15, 125.58, 123.67 (d, *J*_*C-F*_ = 28.7 Hz), 123.24 (d, *J*_*C-F*_ = 3.0 Hz), 122.53 (t, *J*_*C-F*_ = 3.1 Hz), 122.41, 119.12, 118.41, 118.22, 116.17, 111.87, 49.66, 46.11, 44.16, 43.21, 42.95, 33.25, 32.89, 31.60, 29.41, 29.33, 29.27, 29.22, 28.82, 28.68, 27.05, 26.99, 25.22. HRMS (ESI) for C_46_H_48_O_6_N_8_F [M+H]^+^, calcd: 827.3663; found: 827.36754. HPLC purity: 100%, 10.35 min.

### 4.2. Cell Line

PC3 and H660 human prostate cancer cell lines were procured from the American Type Culture Collection (ATCC), while LTL-331R-CL, a neuroendocrine cell line model developed in-house, was also utilized. These cell lines were cultured under standard conditions of 5% CO_2_ at 37°C, following the medium specifications provided by ATCC. The LTL-331R-CL cells were maintained in a similar medium as the H660 cells. Prior to experimentation, all cell lines underwent mycoplasma testing and were authenticated through genotyping procedures.

### 4.3. Western Blot Analysis

Cell lysates were collected in Pierce radioimmunoprecipitation assay (RIPA) buffer (Thermo Scientific), supplemented with protease and phosphatase inhibitor cocktails. Protein concentration was determined utilizing the Pierce 660 nm Protein Assay Reagent (Thermo Scientific). Subsequently, denatured lysates were resolved on NuPage 4–12% Bis-Tris Midi protein gels (Novex) and transferred onto 0.45 μm nitrocellulose membrane (Thermo Scientific) employing a TransBlot Turbo dry transfer system (Bio-Rad). The membrane was blocked in 5% non-fat dry milk blocking buffer for 1 hour at ambient temperature, followed by overnight incubation with primary antibodies at 4°C. Signal detection was achieved using the ECL prime (Amersham) detection kit, and visualization was performed utilizing an Odyssey imaging system (Li-Cor). Signal intensity quantification of TRIB2 was analyzed through the image studio software and normalized by the signal of loading control from the corresponding blots. Primary antibodies targeting TRIB2 (CST, 13533S), cPARP (CST, 5625S), PARP (CST, 9542S), GAPDH (CST, 3683S), and H3 (CST, 3638S) were procured from cell signaling technologies (CST).

### 4.4. TMT-Based Quantitative Proteomic Analysis

LTL-331R-CL cells were seeded at a density of 5 × 10^6^ cells per well in a 6-well plate and incubated overnight. Subsequently, cells were treated with either DMSO or compound **5k** at a concentration of 0.5 μM for 4 hours. Whole-cell lysates were harvested in RIPA buffer (Thermo Scientific) supplemented with protease inhibitors. Total protein extracted from three technical replicates was subjected to trypsin digestion followed by labeling with 6-plex Tandem Mass Tag (TMT) label reagent (Thermo Scientific), adhering to the manufacturer’s protocol. The labeled protein samples were subjected to fractionation using liquid chromatography-mass spectrometry (LC-MS/MS) to identify differentially expressed proteins.

### 4.5. CETSA Assay

PC3 cells were plated at a density of 2 × 10^7^ cells per 15 cm plate. The following day, cells were treated with either DMSO or 2 μM **5k** for 3 hours. Subsequently, the cells were detached using trypsin, rinsed with PBS, and resuspended in 700 μL of PBS supplemented with a protease inhibitor cocktail. Samples of 100 μL each from the DMSO and **5k**-treated cells were subjected to heat treatment at temperatures ranging between 45–50°C for 3 minutes using a thermal cycler. Following the heat treatment, the tubes were incubated at room temperature for 3 minutes before snap-freezing using liquid nitrogen. The cells underwent three cycles of freeze-thawing thrice and were then centrifuged at 13000 rpm for 10 minutes. The resulting supernatant was utilized for immunoblotting analysis.

### 4.6. Radiometric Kinase Assay

Human TRIB2 recombinant protein is incubated with 8 mM MOPS pH 7.0, 0.2 mM EDTA, 250 μM RRRFRPASPLRGPPK, 10 mM Magnesium acetate, [γ−33P-ATP], and the annotated compounds (specific activity and concentration as required). The reaction is initiated by the addition of the Mg/ATP mix. After incubation for 120 minutes at room temperature, the reaction is stopped by the addition of phosphoric acid to a concentration of 0.5%. An aliquot of the stopped reaction is spotted onto a filter and washed four times for 4 minutes in 0.425% phosphoric acid and once in methanol prior to drying and scintillation counting.

### 4.7. Proliferation Assays

PC3 and LTL-331R-CL cells were seeded in a 96-well plate at densities of 1000 and 10,000 cells per well, respectively. The following day, cells were treated with DMSO, **5k**, **5m**, **1**, or pomalidomide at 0.5 μM, with each condition conducted in five independent replicate wells. Cell viability was evaluated at various time points using CellTiter-Glo (CTG) reagent (Promega), according to the manufacturer’s instructions. The luminescent signal was measured using the Infinite M1000 Pro plate reader (Tecan).

### 4.8. IC_50_ Determination

PC3 and LTL-331R-CL cells were plated in 96-well plate at 1000 and 5,000 cells per well, respectively. Cells were treated with compound **5k**, **5m**, or **1** at a concentration of 30 μM for PC3 and at a concentration of 5 μM for LTL331RCL cells, followed by serial dilutions in six replicates per dilution. On day 5, cell viability was evaluated using CTG reagent and the luminescent signal was measured using the Infinite M1000 Pro plate reader (Tecan). Graphs are generated using GraphPad Prism software.

### 4.9. Computational Method

Protein structure of TRIB2 (PDB ID: 7UPM) was obtained from the Protein Data Bank (http://www.rcsb.org) and prepared using the Protein Preparation Wizard (Schrödinger, LLC, New York, NY, 2022). Small molecules were prepared using LigPrep. Molecular docking was performed using Glide standard precision (SP) using default settings.

## Supplementary Material

Figure,NMR Spectra, HRMS Spectra and HPLC Purity Data,Unprocessed western blots

Appendix A. Supplementary data

The original Western blot for preliminary screening; ^1^H NMR and ^13^C NMR spectra for all TRIB2 degraders; HPLC traces for all TRIB2 degraders; the unprocessed western blots in the manuscript; the statistical raw data from applicable figure panels; the data set of TMT proteomics.

## Figures and Tables

**Table 1.** SAR study of TRIB2 PROTACs based on VHL ligand.

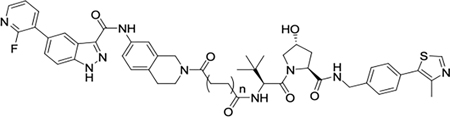		

Comp.	n	% Protein degradation

**4a**	*n* = 1	ND^a^
**4b**	*n* = 2	ND^a^
**4c**	*n* = 3	ND^a^
**4d**	*n* = 4	ND^a^
**4e**	*n* = 5	ND^a^

a ND, protein degradation is not detected in PC3 cells immunoblot analysis.

**Table 2:** SAR study of TRIB2 PROTACs based on CRBN ligand.

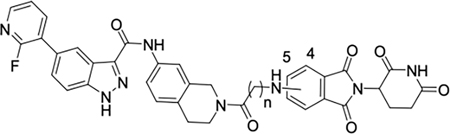				

Comp.	n	Position	% TRIB2 degradation	
			1 nM	100 nM

**5a**	*n* = 1	4	ND^a^	ND^a^
**5b**	*n* = 3	4	ND^a^	ND^a^
**5c**	*n* = 5	4	ND^a^	ND^a^
**5d**	*n* = 7	4	ND^a^	ND^a^
**5e**	*n* = 9	4	ND^a^	ND^a^
**5f**	*n* = 11	4	ND^a^	ND^a^
**5g**	*n* = 1	5	ND^a^	ND^a^
**5h**	*n* = 3	5	ND^a^	84.1
**5i**	*n* = 5	5	24.5	85.6
**5j**	*n* = 7	5	ND^a^	58.2
**5k**	*n* = 9	5	ND^a^	92
**5l**	*n* = 11	5	ND^a^	ND^a^
**5m**	*n* = 9	5	ND^a^	ND^a^

a ND, protein degradation is not detected in PC3 cells using immunoblot analysis.

## Data Availability

Data will be made available on request.

## Abbreviations

AKT: V-akt murine thymoma viral oncogene homolog
AP-4: Activating enhancer binding protein 4
ATP: Adenosine triphosphate
CAMK: Calcium calmodulin dependent protein kinases
CETSA: Cellular thermal shift assay
CHX: Cycloheximide
CRBN: Cereblon
DC_50_: Half maximal degradation concentration
DCM: Dichloromethane
DIPEA: *N,N*-diisopropylethylamine
D_max_: Maximum degradation ratio
DHP: 2*H*-pyrandichloropropene
DMF: *N,N*-dimethylformamide
DMSO: Dimethyl sulfoxide
ERK: Extracellular signal-regulated kinases
ESI-MS: Low-resolution electrospray ionization mass spectrometry
HATU: *O*-(7-azabenzotriazol-1-yl)-*N,N,N’,N’*-tetramethyluronium Hexafluorophosphate
HPLC: High-performance liquid chromatography
IC_50_: Half maximal inhibitory concentration
MAPK: Mitogen-activated protein kinase
NE: Neuroendocrine
OCT3/4: Octamer binding transcription factor
PEST domain: Pro/Glu/Ser/Thr-rich domain
POI: Protein-of-interest
PARP: Poly (ADP-ribose) polymerase
RIPA: Pierce radioimmunoprecipitation assay
PTSA: 4-toluenesulfonic acid
PROTACs: Proteolysis targeting chimeras
STK40: Serine/Threonine Kinase 40
TFA: Trifluoroacetic acid
TRIB2: Tribbles pseudokinase 2
TLC: Thin-layer chromatography
TEA: Triethylamine
THP: Tetrahydropyran
TMS: Tetramethylsilane
UPS: Ubiquitin proteasome system
VHL: Von Hippel-Lindau
