# Supplementary material for: Discovery of the First Selective and Potent PROTAC Degrader for the Pseudokinase TRIB2
Source: Eur J Med Chem. Author manuscript; Available in PMC 2025 Jun 4. (PMC12135709; doi:10.1016/j.ejmech.2024.117016)
Supplement: Figure,NMR Spectra, HRMS Spectra and HPLC Purity Data,Unprocessed western blots [file NIHMS2082142-supplement-Figure_NMR_Spectra__HRMS_Spectra_and_HPLC_Purity_Data_Unprocessed_western_blots.docx]

Supporting Information

Discovery of the First Selective and Potent PROTAC Degrader for the Pseudokinase TRIB2

Chaowei Wen^a, #^, Prathibha R. Gajjala^b, c, #^, Yihan Liu^b, d, #^, Bingzhong Chen^a^, Mehtab S. Bal^b^, Payal Sutaria^b^, Qiao Yuanyuan^b, c^, Yang Zheng^b, c^, Yang Zhou^a^, Jinwei Zhang^e^, Weixue Huang^e^, Xiaomei Ren^e^, Zhen Wang^e^, Ke Ding^a, e,^ *, Arul M. Chinnaiyan^b, c, f, g, h,^*, Fengtao Zhou^a,^ *

*^a^*International Cooperative Laboratory of Traditional Chinese Medicine Modernization and Innovative Drug Development, Ministry of Education (MoE) of People’s Republic of China, College of Pharmacy, Jinan University, 601 Huangpu Avenue West, Guangzhou 510632, China

^b^Michigan Center for Translational Pathology, University of Michigan, Ann Arbor, Michigan 48109, United States

^c^Department of Pathology, University of Michigan, Ann Arbor, Michigan 48109, United States

^d^Cancer Biology Program, University of Michigan, Ann Arbor, Michigan 48109, United States

^e^State Key Laboratory of Chemical Biology, Shanghai Institute of Organic Chemistry, University of Chinese Academy of Sciences, Chinese Academy of Sciences, 345 Lingling Road, Shanghai 200032, China

^f^Rogel Cancer Center, University of Michigan, Ann Arbor, Michigan 48109, United States

^g^Department of Urology, University of Michigan, Ann Arbor, Michigan 48109, United States

^h^Howard Hughes Medical Institute, University of Michigan, Ann Arbor, Michigan 48109, United States

^#^These authors contribute equally to the work.

^*^Corresponding authors. E-mail: dingk@sioc.ac.cn; (K. Ding.); [arul@med.umich.edu](mailto:arul@med.umich.edu) (A. M. Chinnaiyan)

fengtaozhou@jnu.edu.cn (F. Zhou.).

**Table of Contents**

**1. Supplementary Figure.……………………………………………………...S2**

**2. Copies of NMR Spectra, HRMS Spectra and HPLC Purity Data……….S3**

**3. Unprocessed western blots ..……………………………………………… S55**

**1. Supplementary Figure**

**
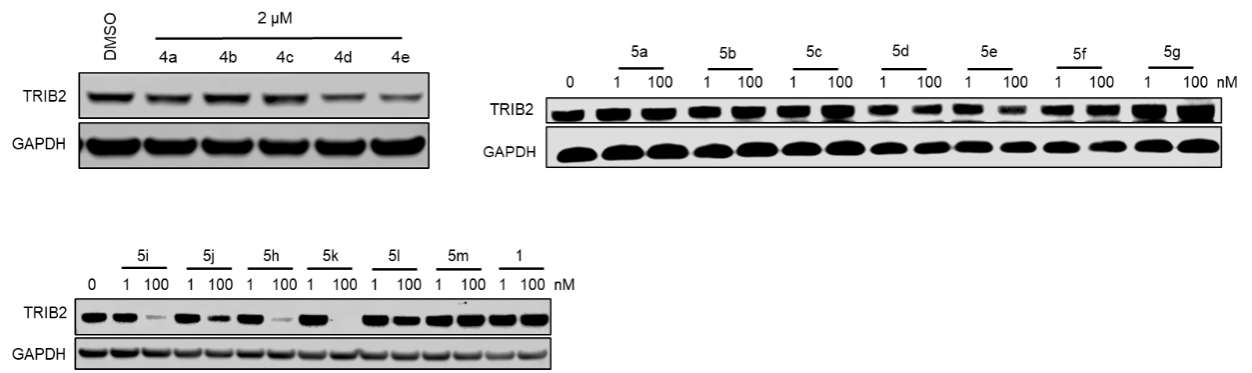
**

**Figure S1.** Preliminary screen of TRIB2 PROTACs. Cells were treated with compound at indicated concentrations for 24 h, and the levels of TRIB2 were detected by western blot analysis.

**2. ^1^H and ^13^C NMR Spectra of compound 1:**


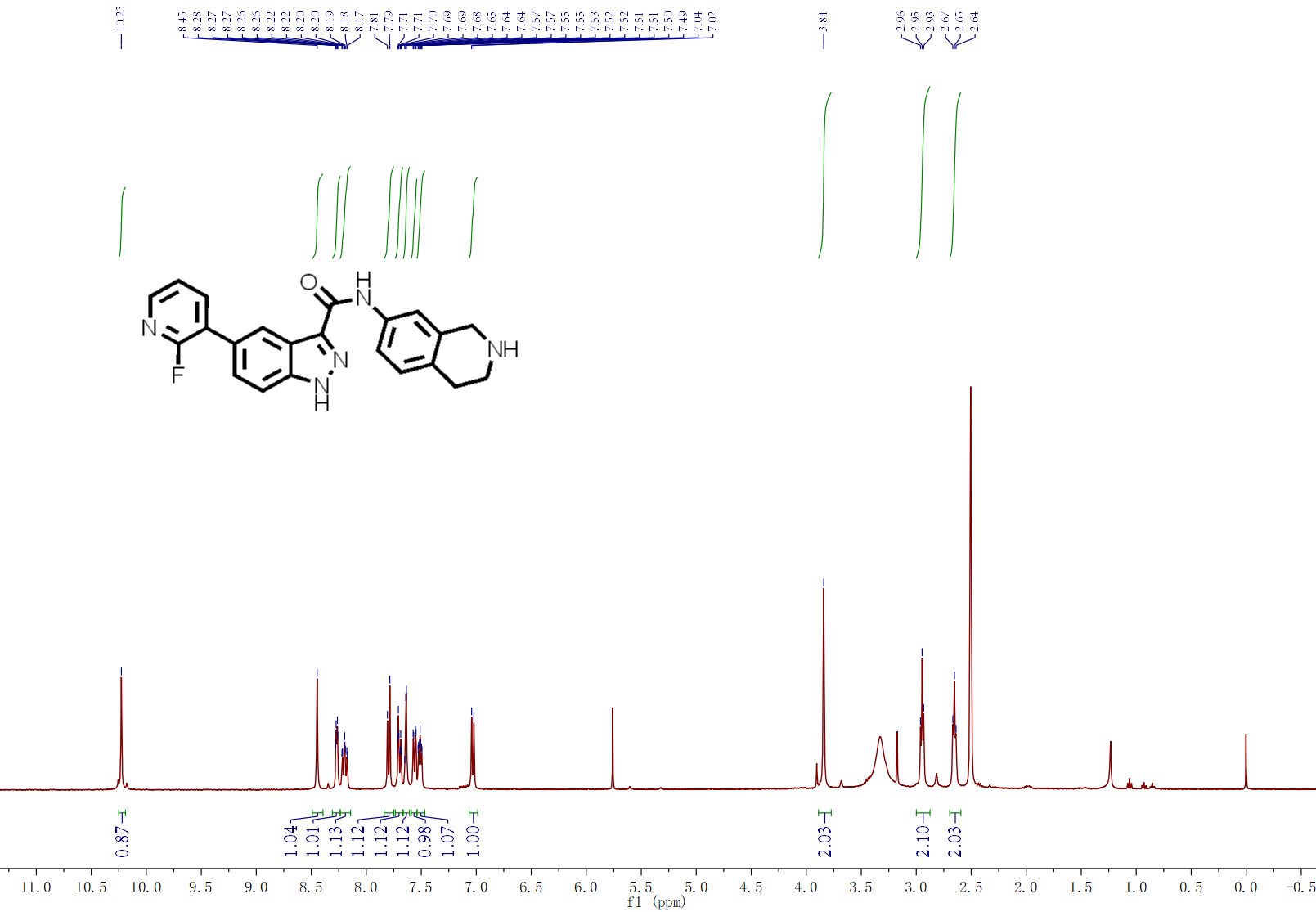

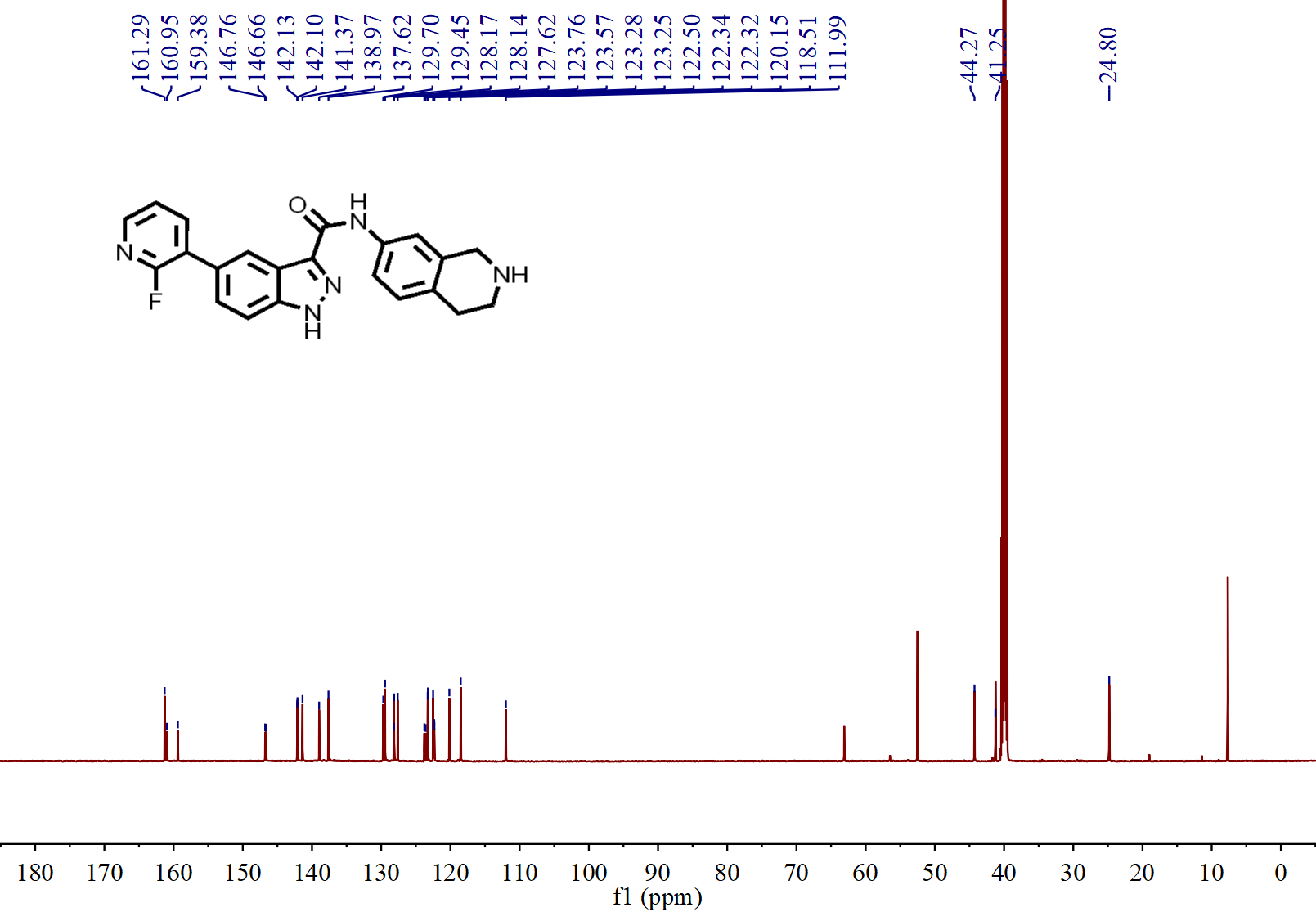


**HRMS Spectra of compound 1：**

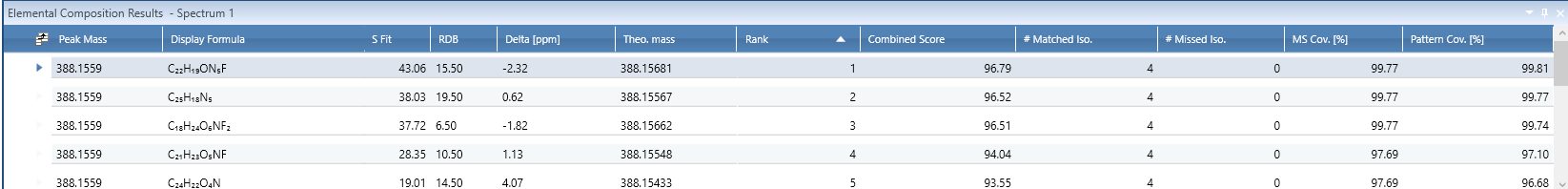


**HPLC Purity Data of compound 1**


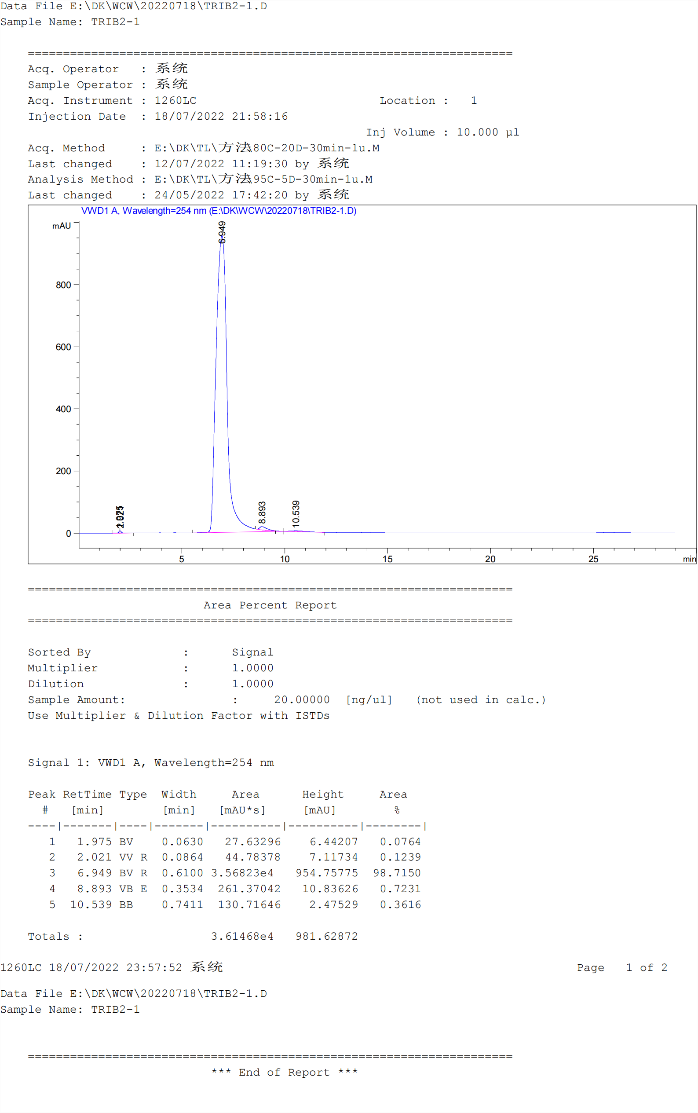


**^1^H and ^13^C NMR Spectra of compound 4a**


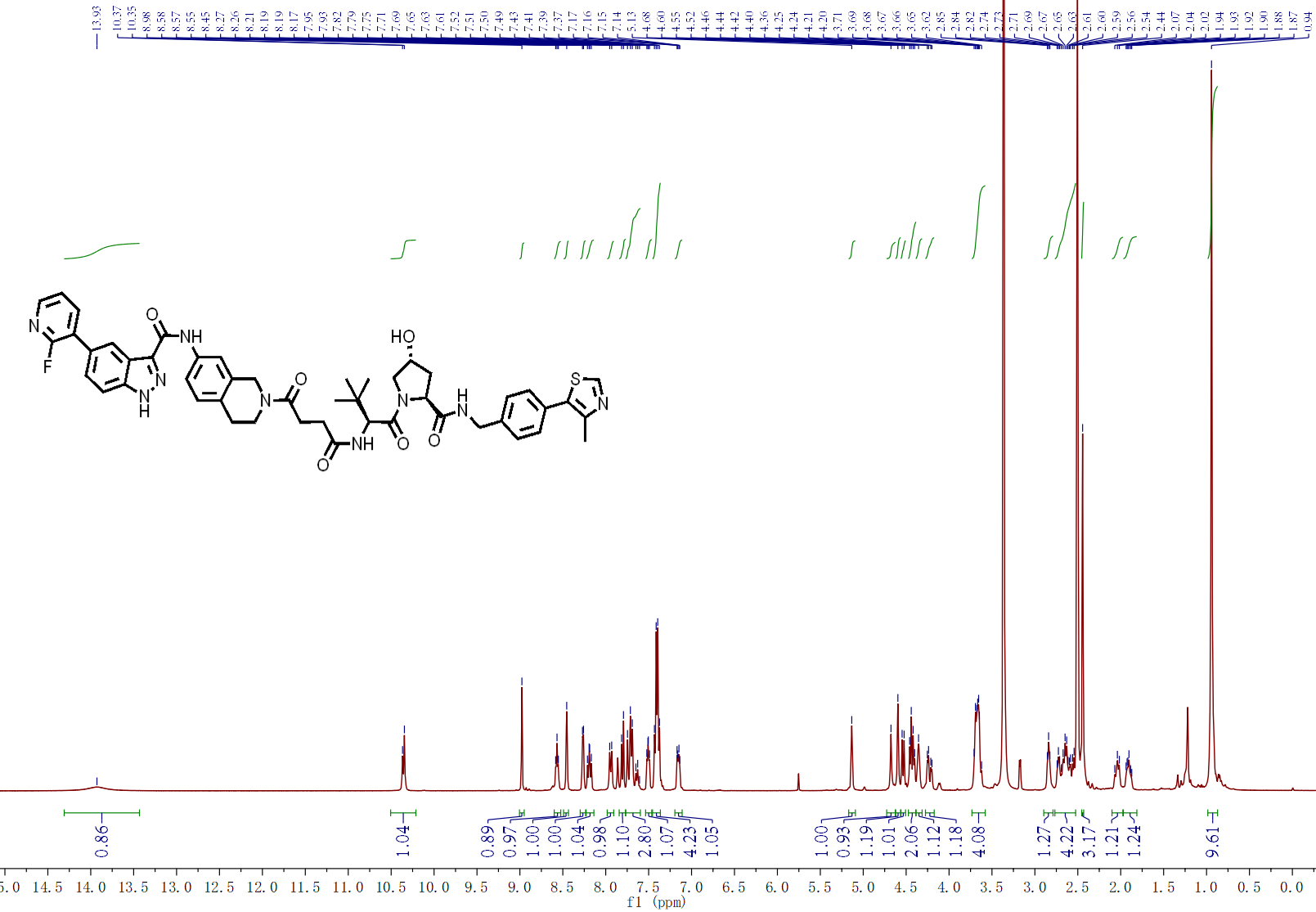

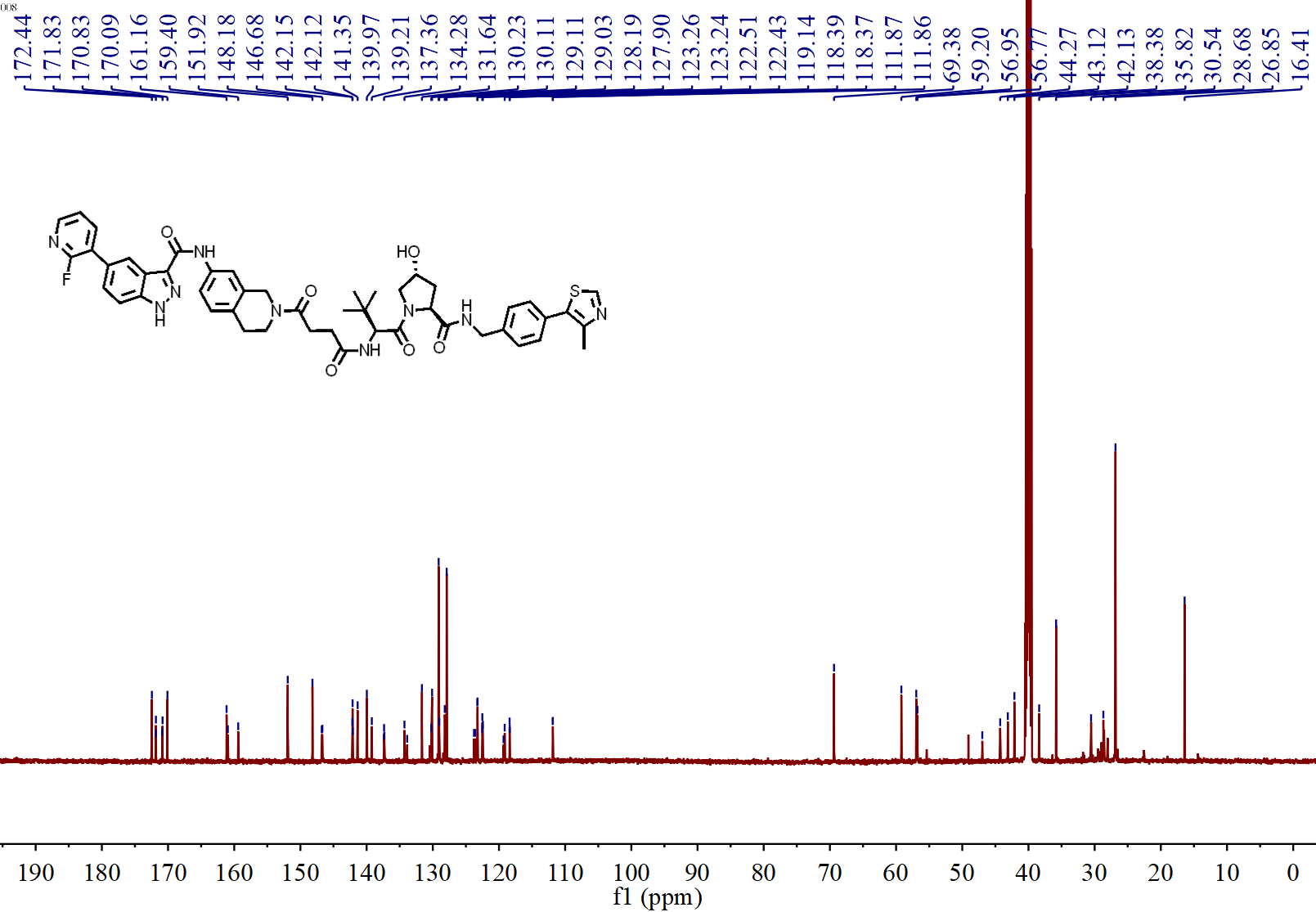


**HRMS Spectra of 4a**

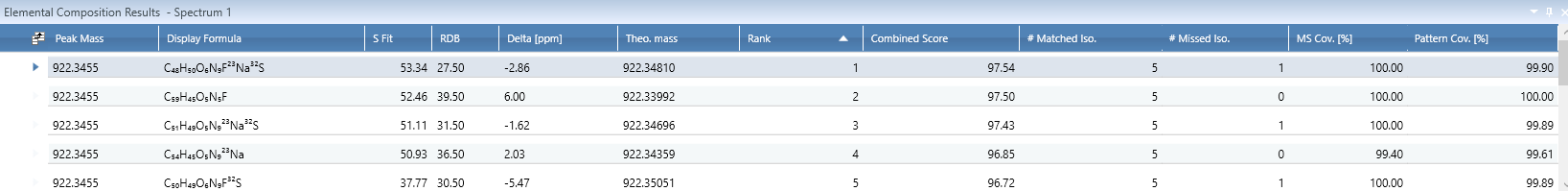


**HPLC Purity Data of 4a**


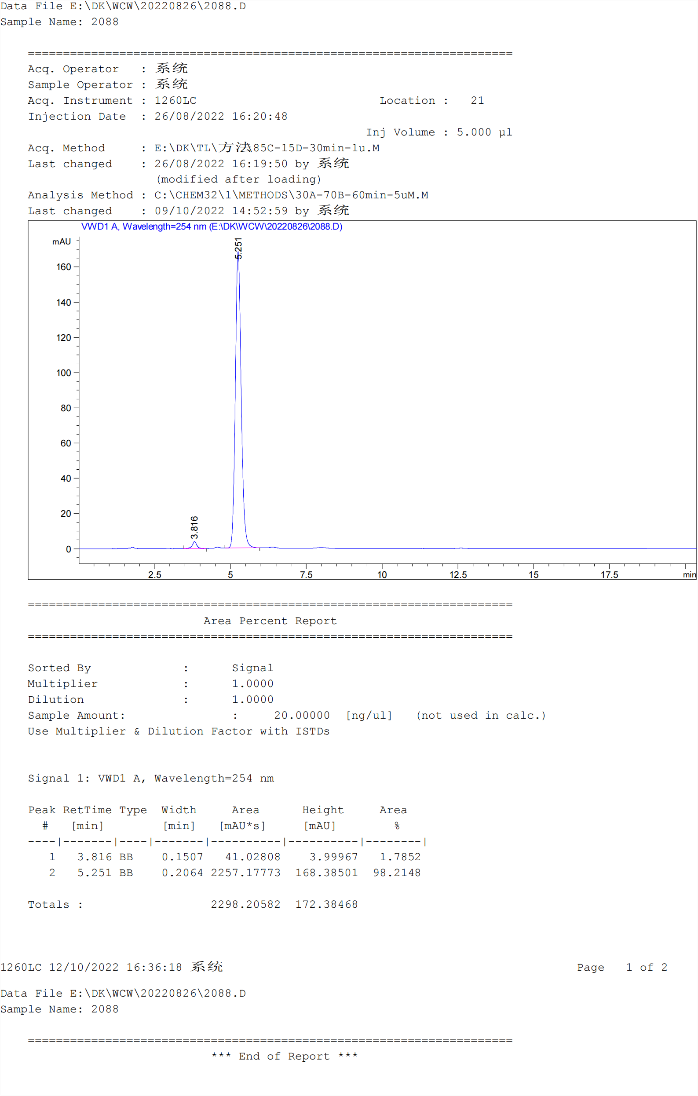


**^1^H and ^13^C NMR Spectra of compound 4b**


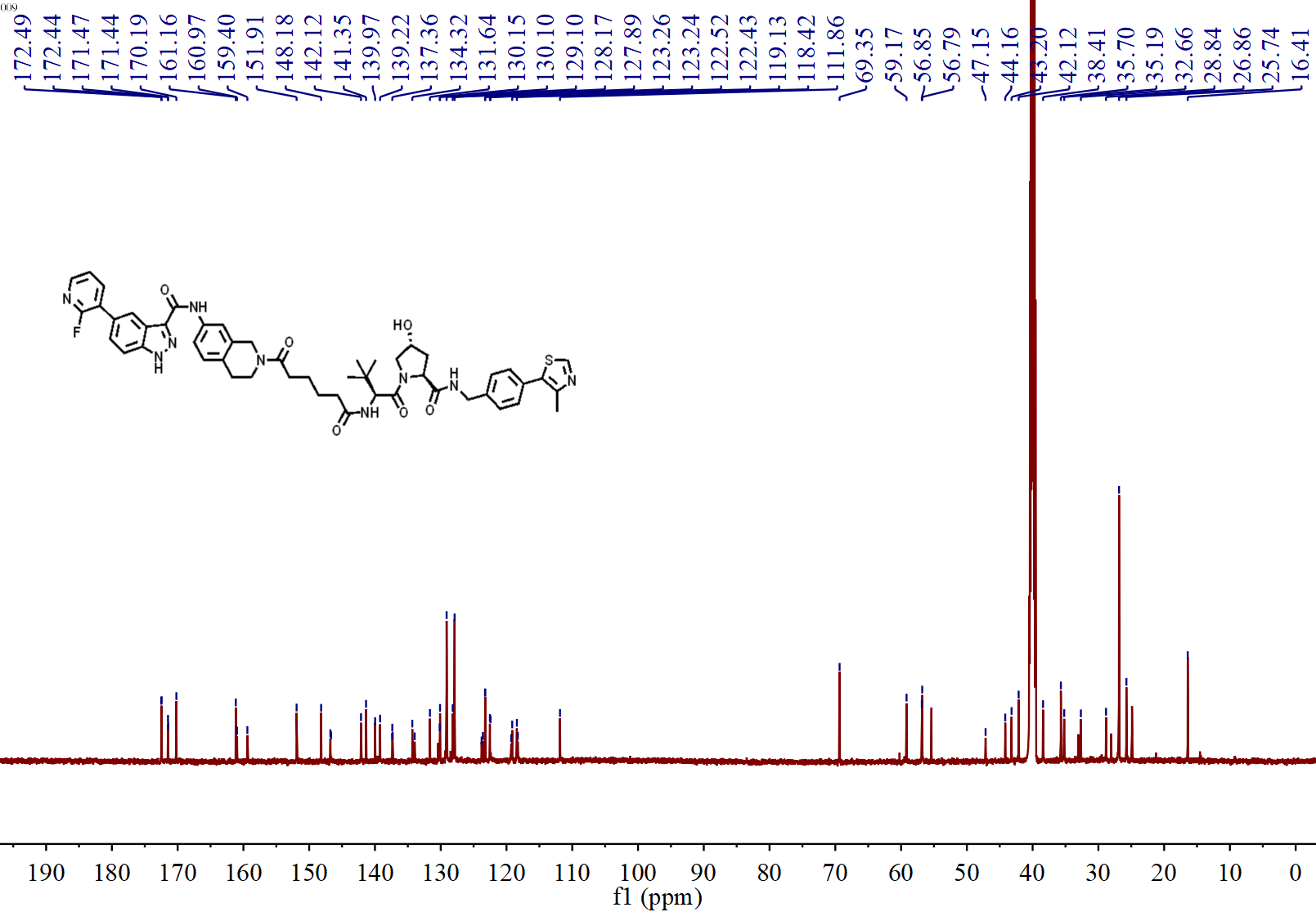

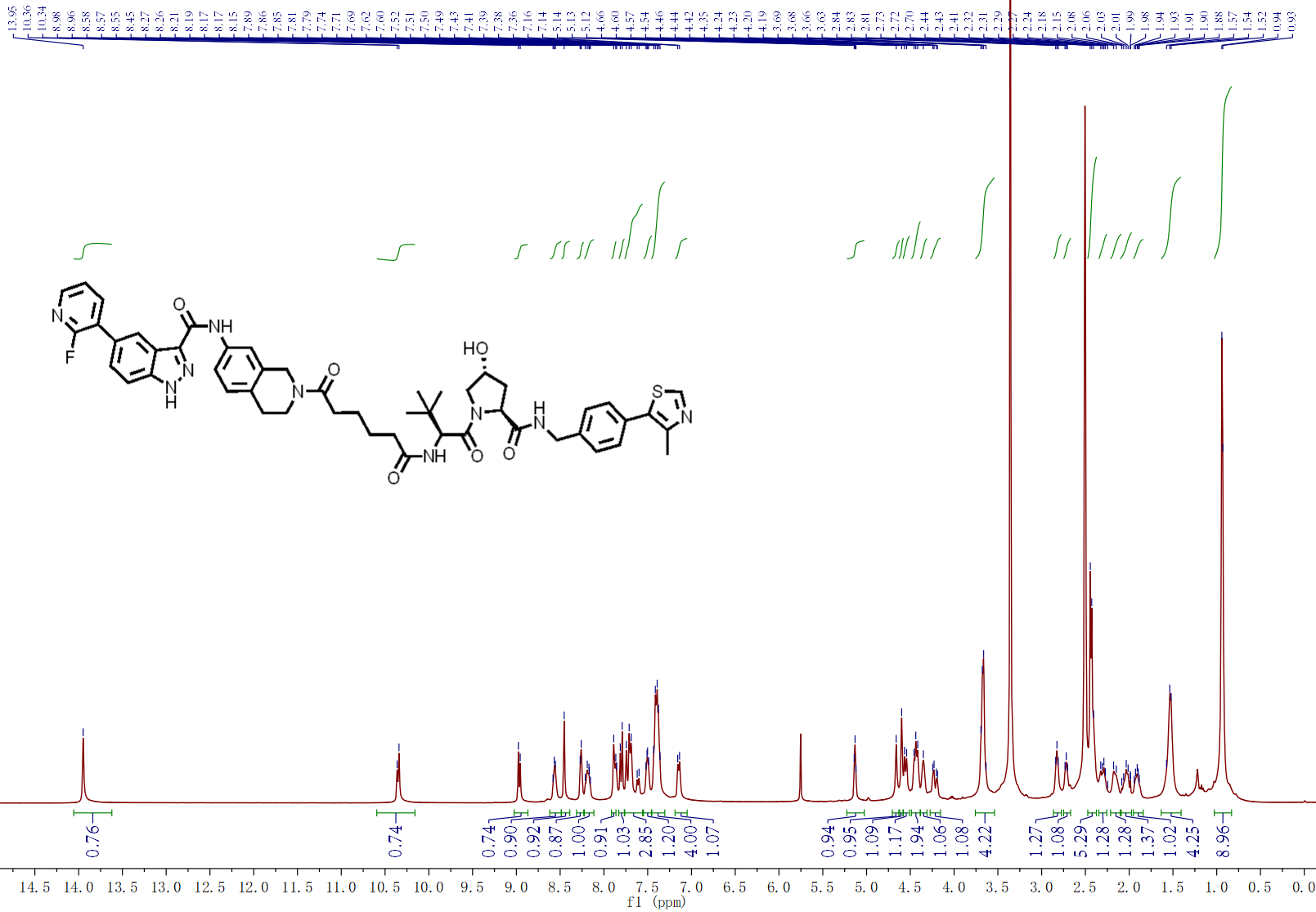


**HRMS Spectra of 4b**

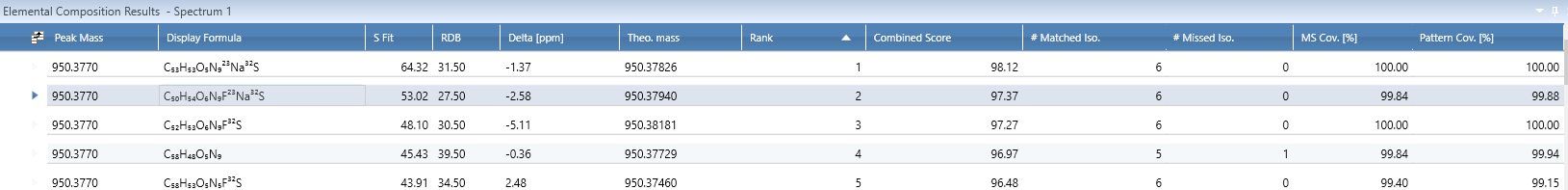


**HPLC Purity Data of 4b**


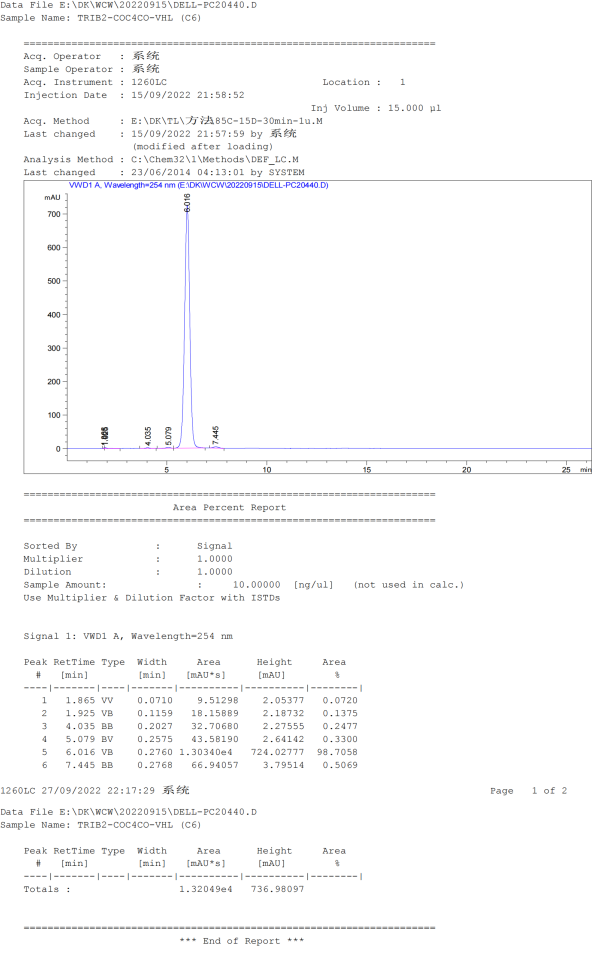


**^1^H and ^13^C NMR Spectra of compound 4c**


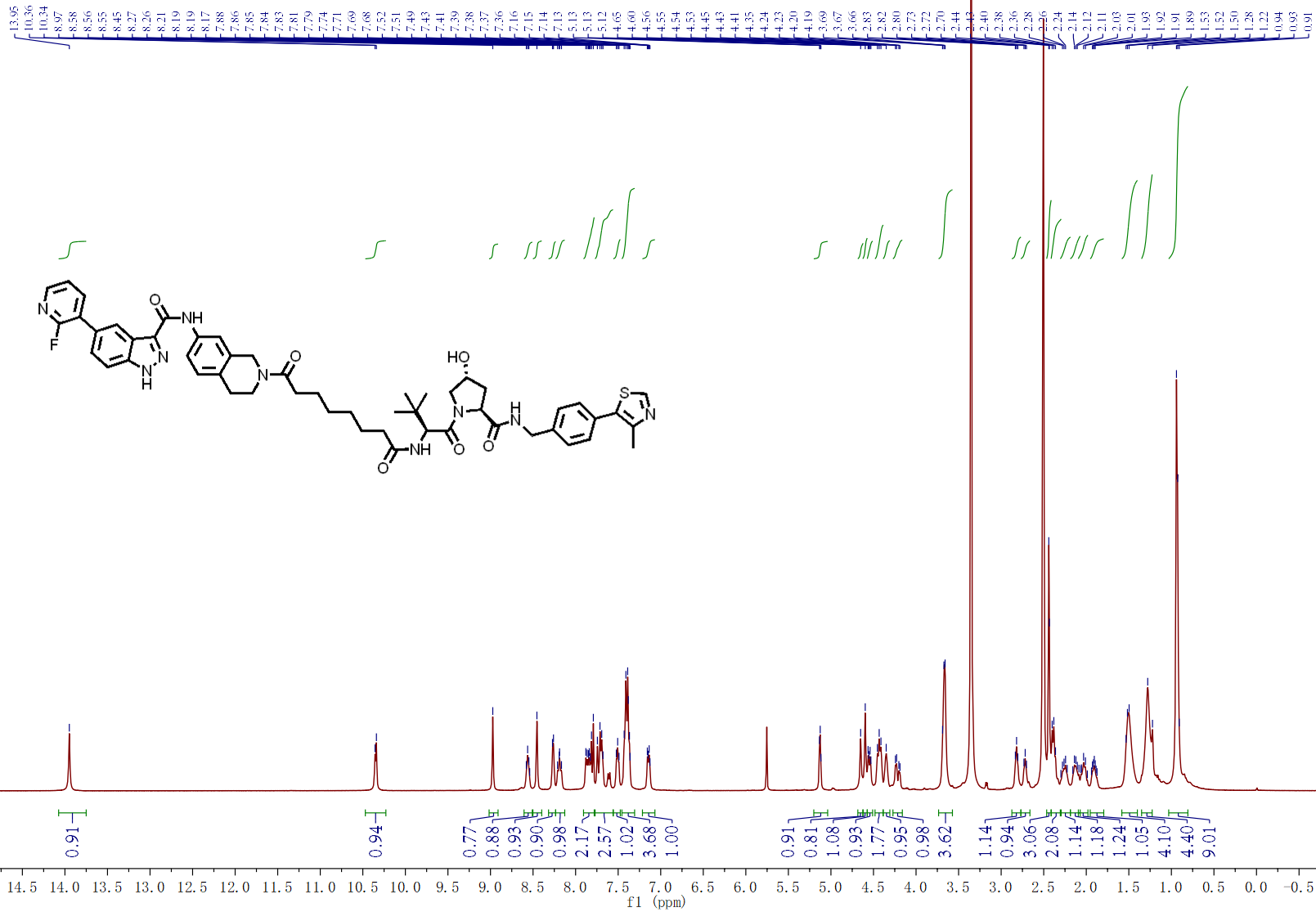


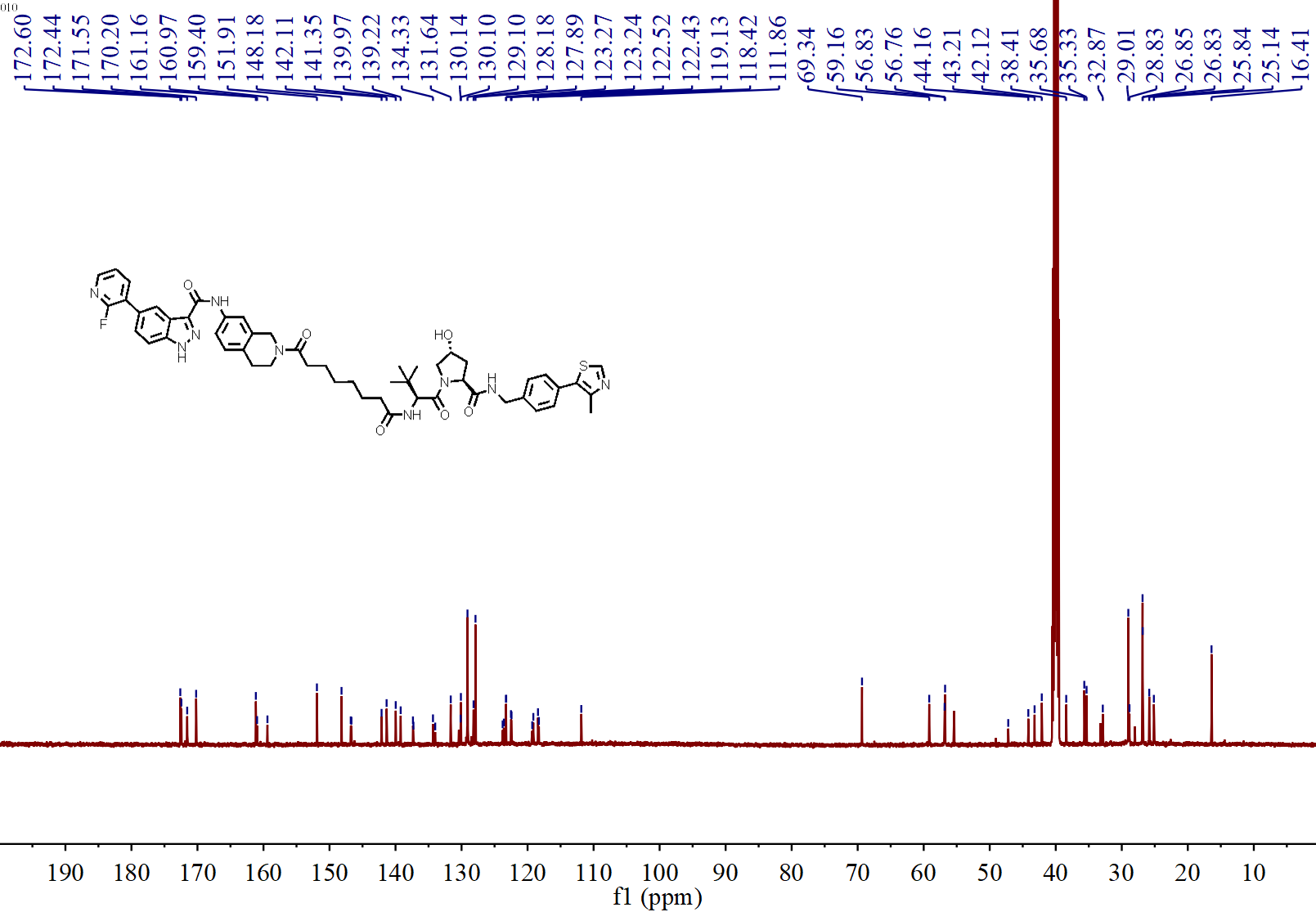


**HRMS Spectra of 4c**

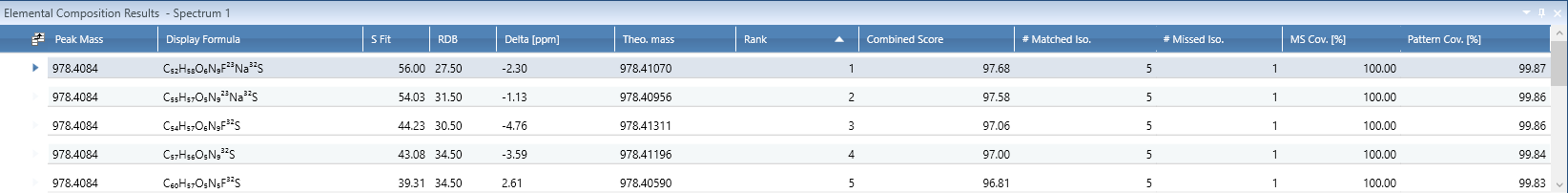


**HPLC Purity Data of 4c**


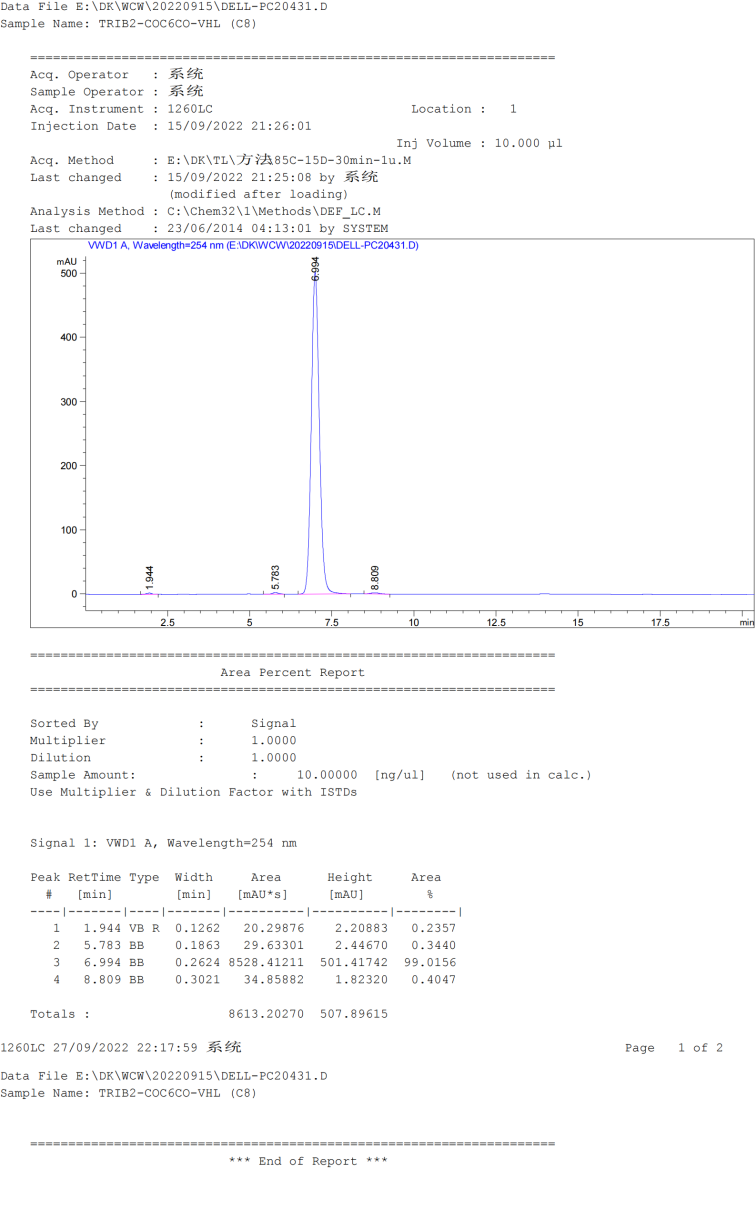


**^1^H and ^13^C NMR Spectra of compound 4d**


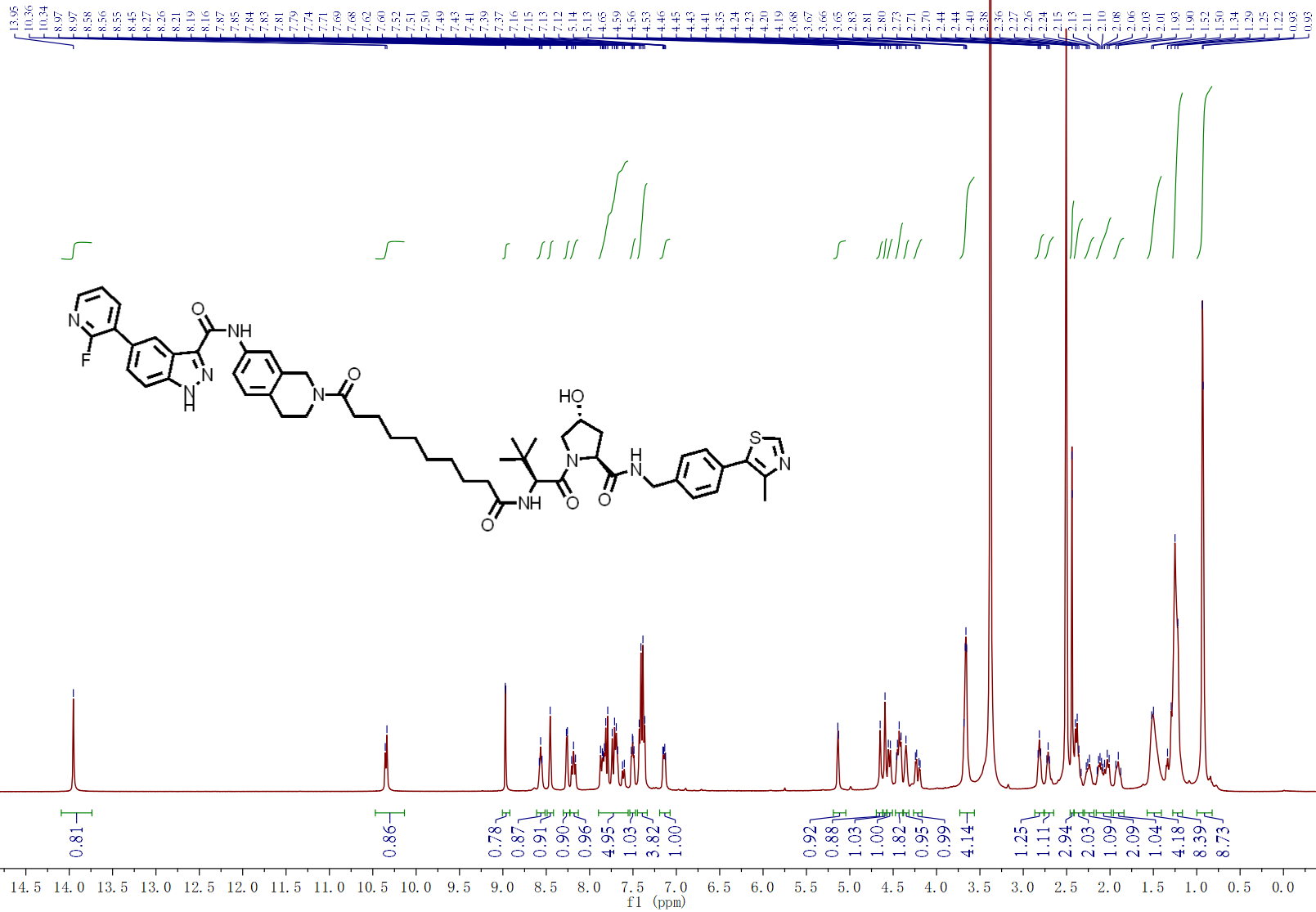

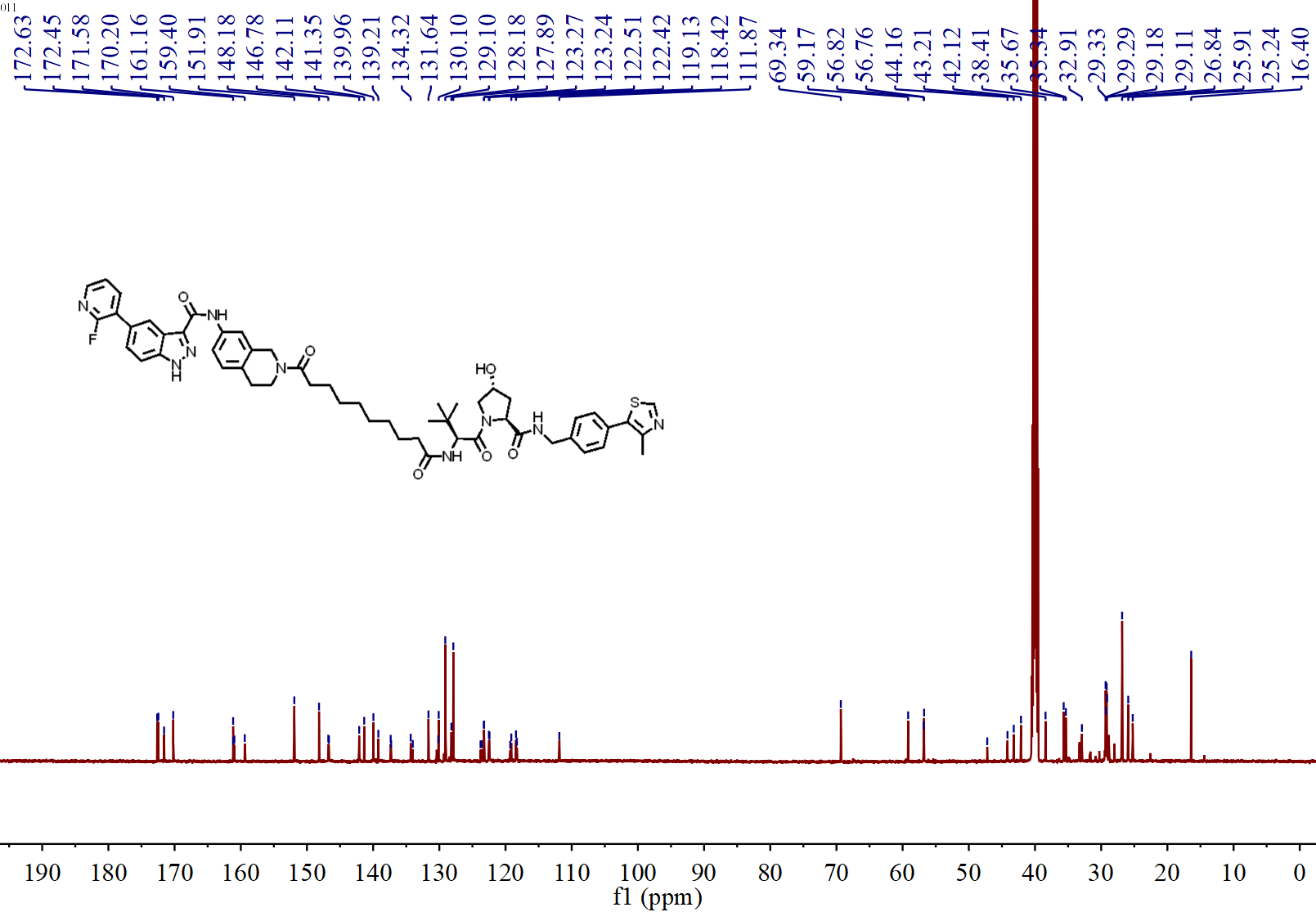


**HRMS Spectra of 4d**

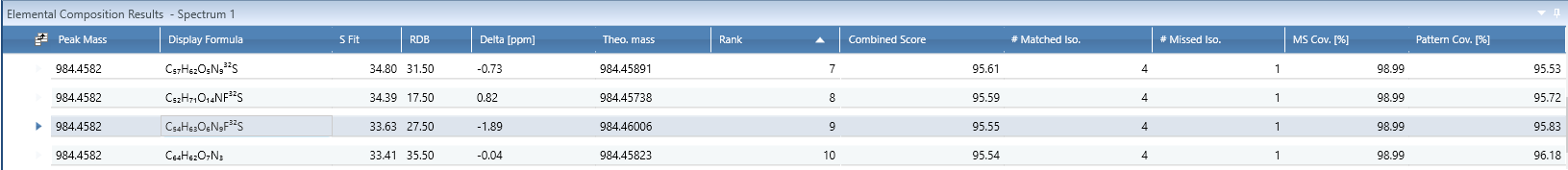


**HPLC Purity Data of 4d**


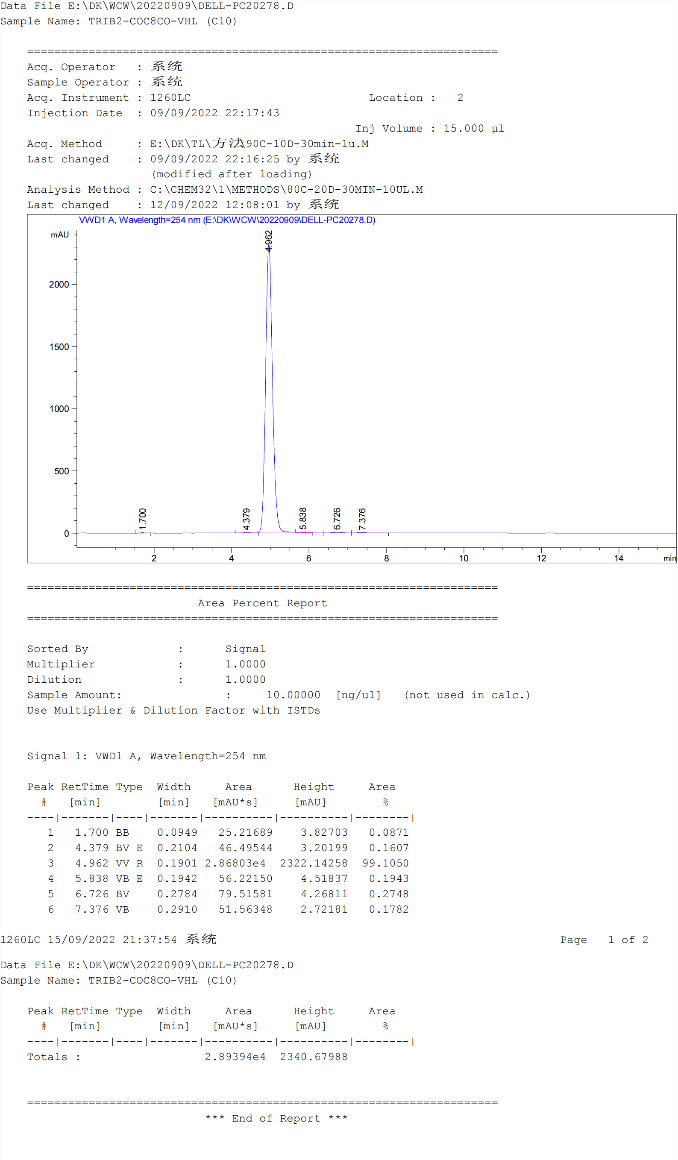


**^1^H and ^13^C NMR Spectra of compound 4e**


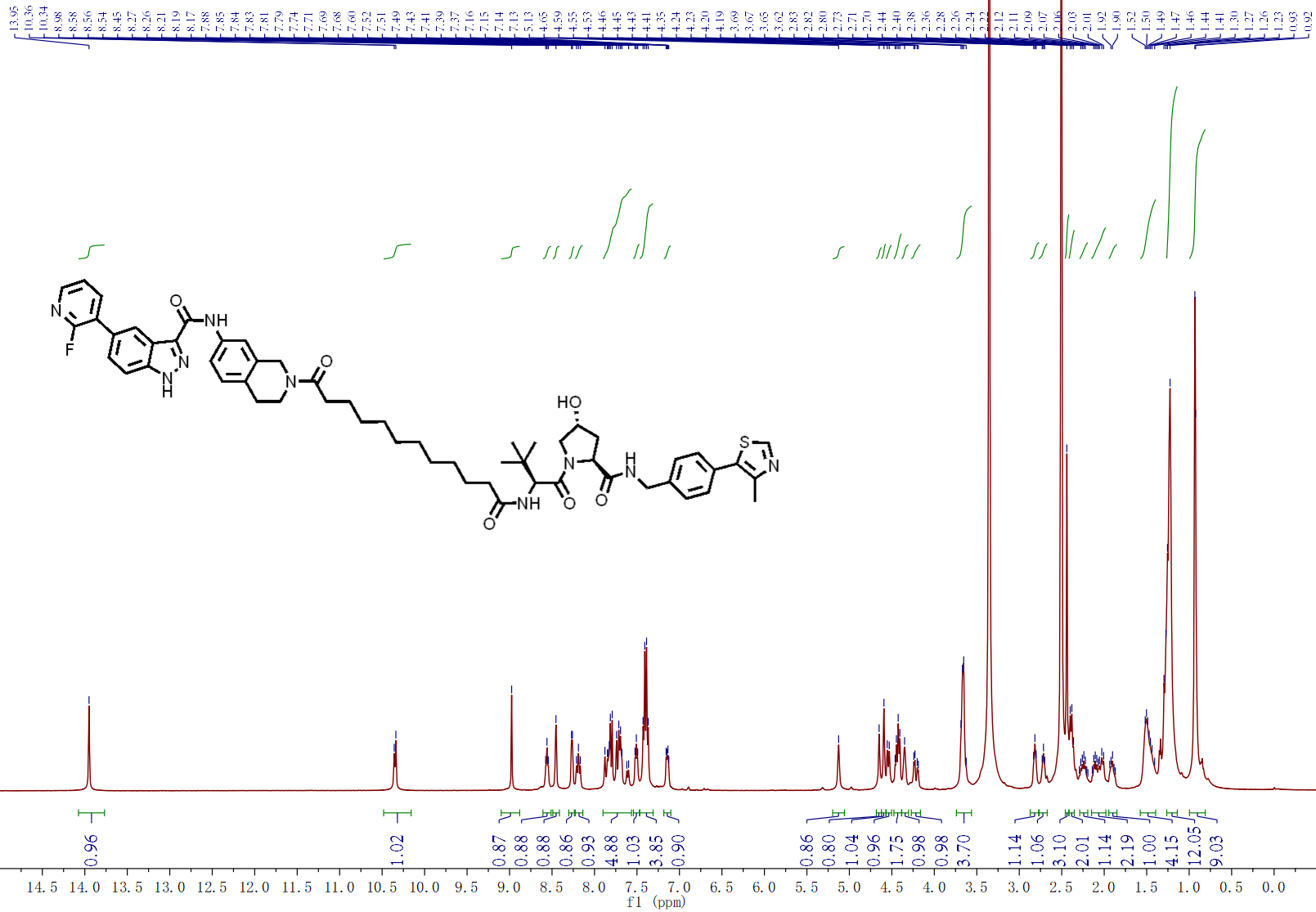

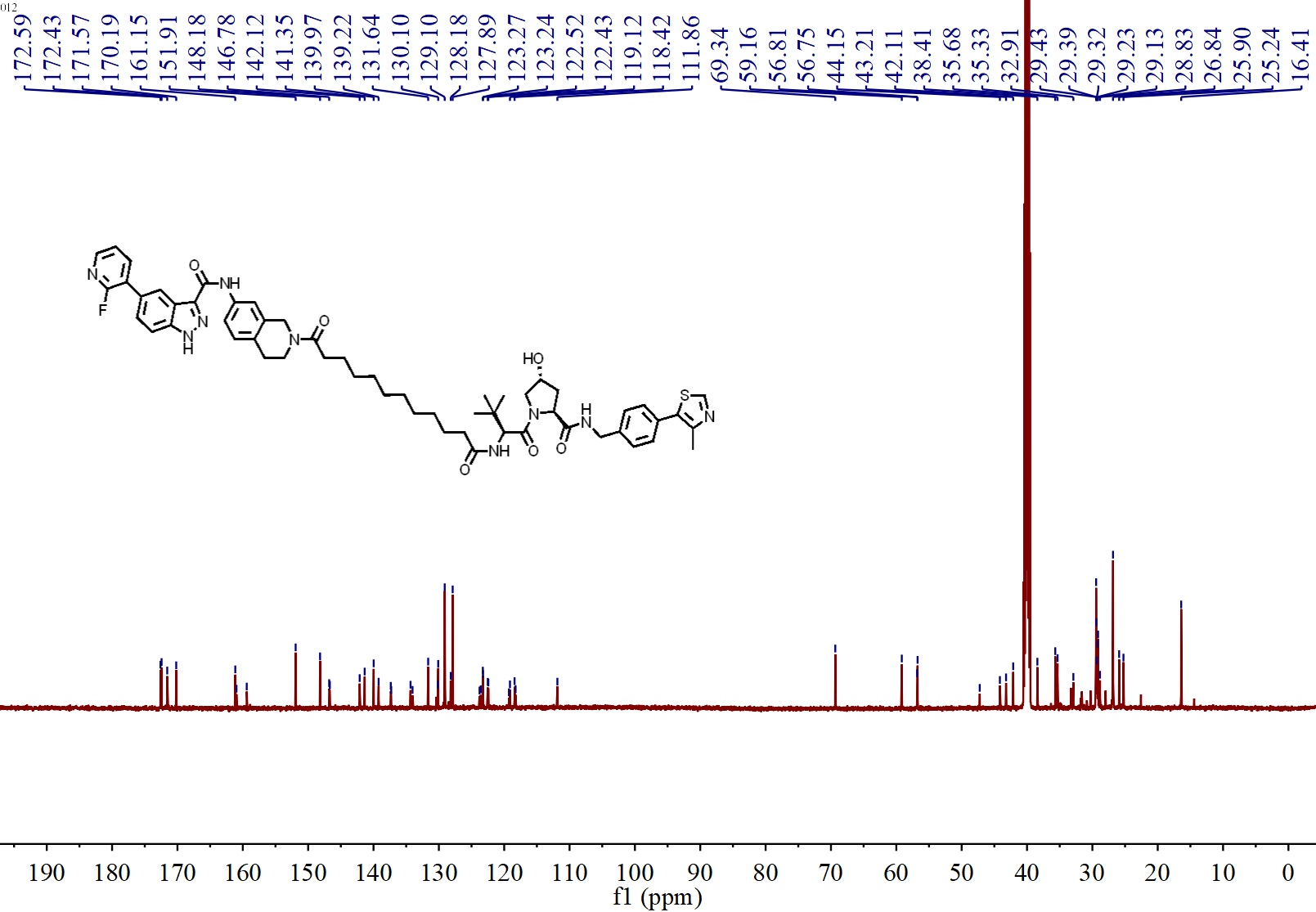


**HRMS Spectra of 4e**

**
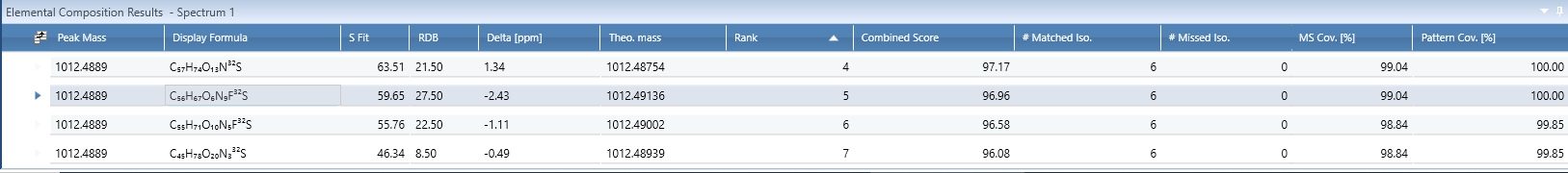
**

**HPLC Purity Data of 4e**


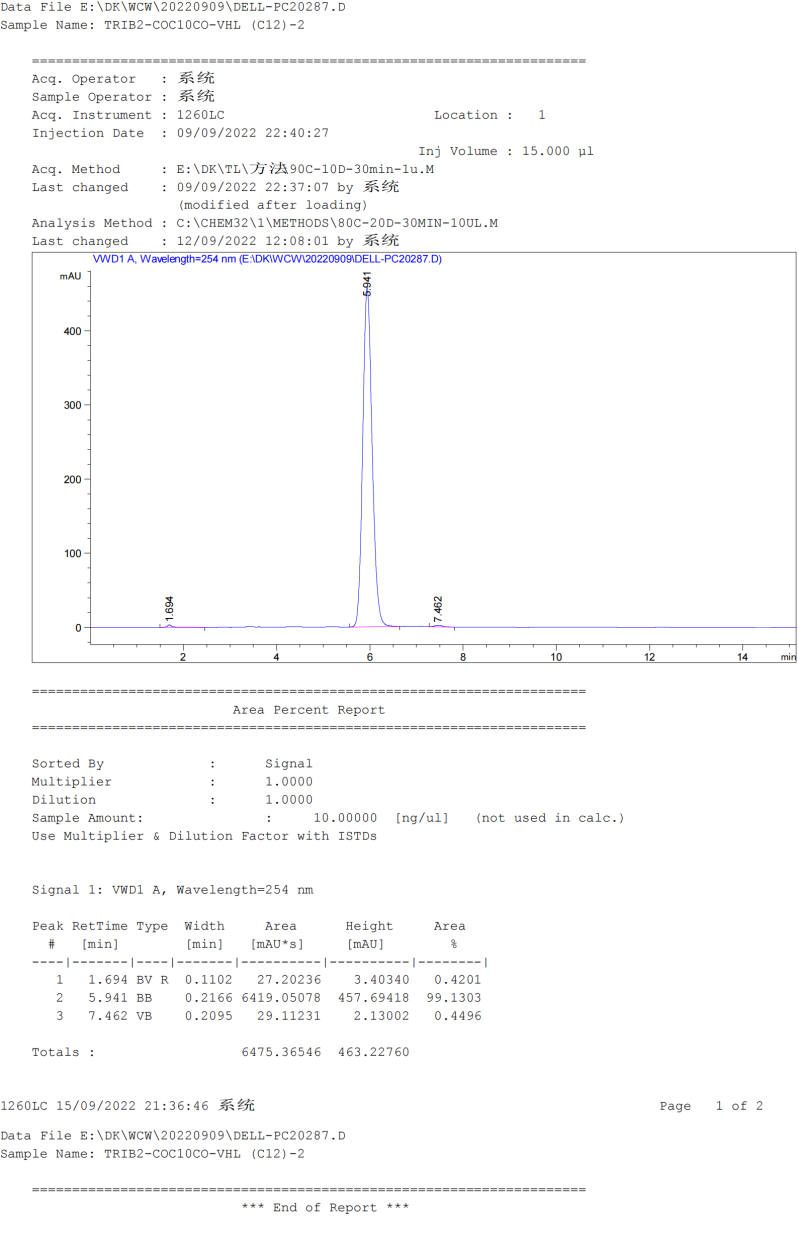


**^1^H and ^13^C NMR Spectra of compound 5a**


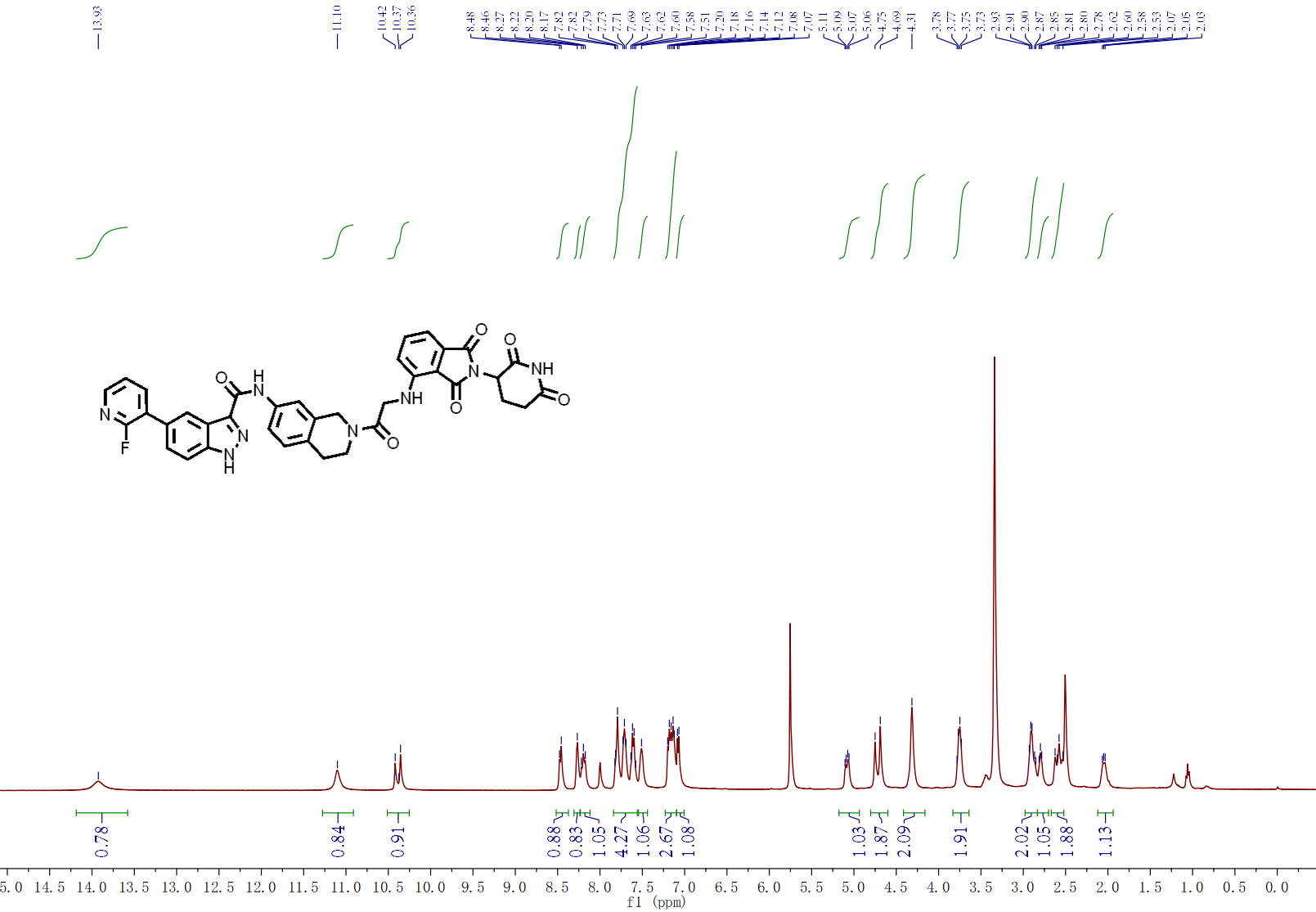


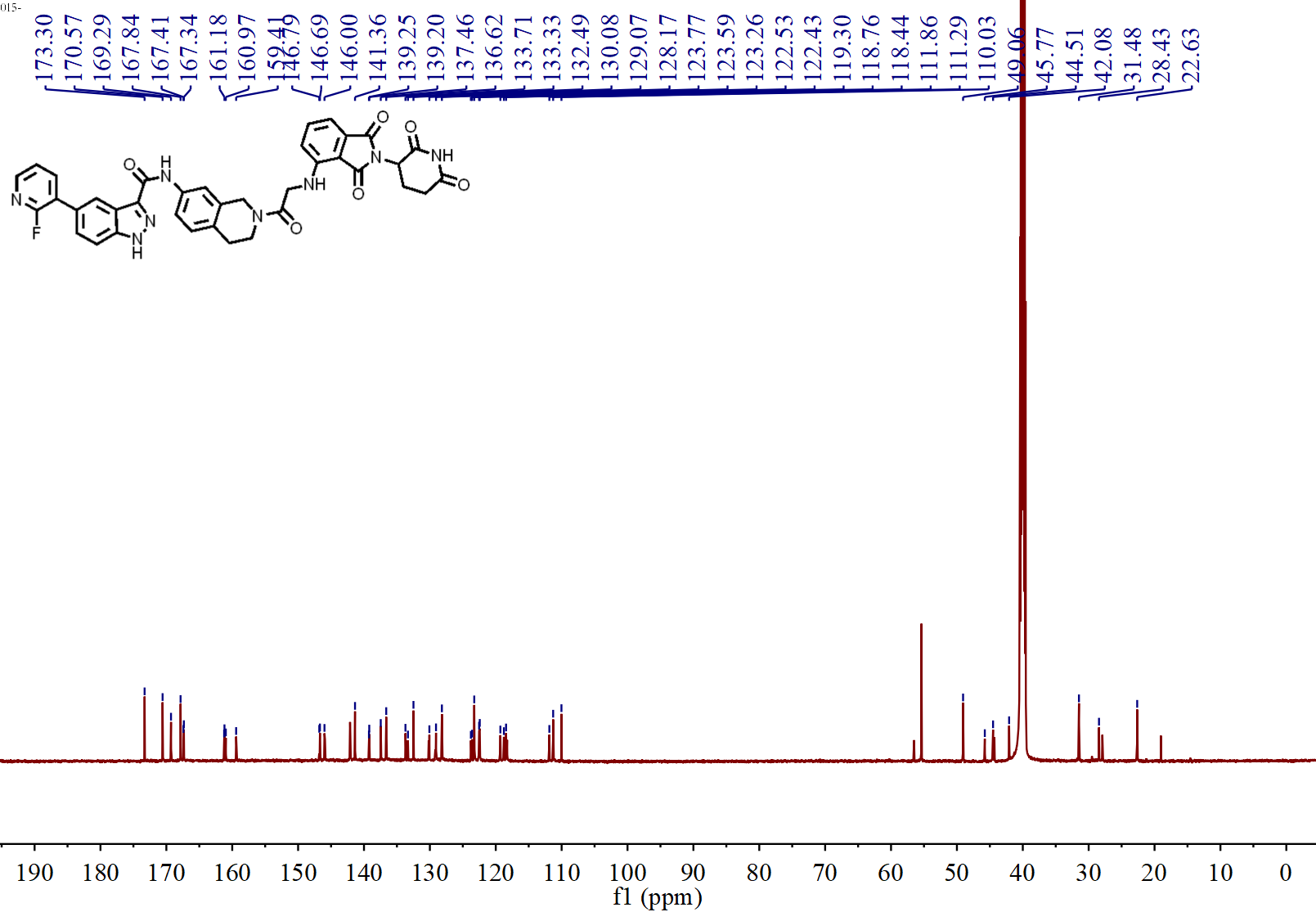


**HRMS Spectra of 5a**

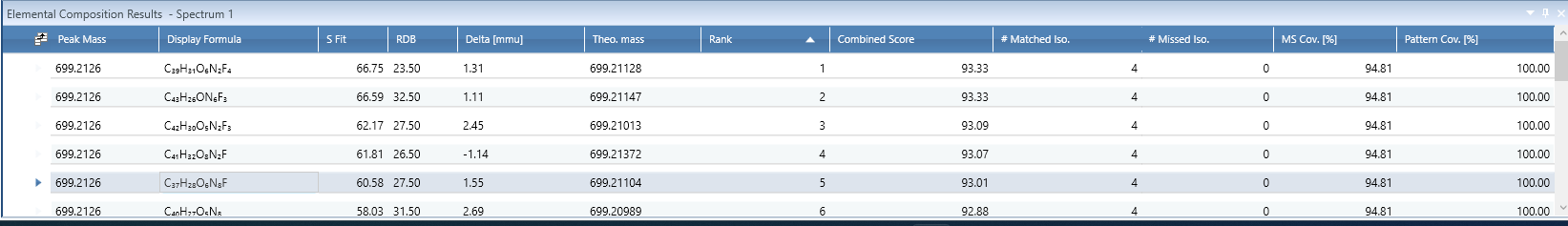


**HPLC Purity Data of 5a**

**
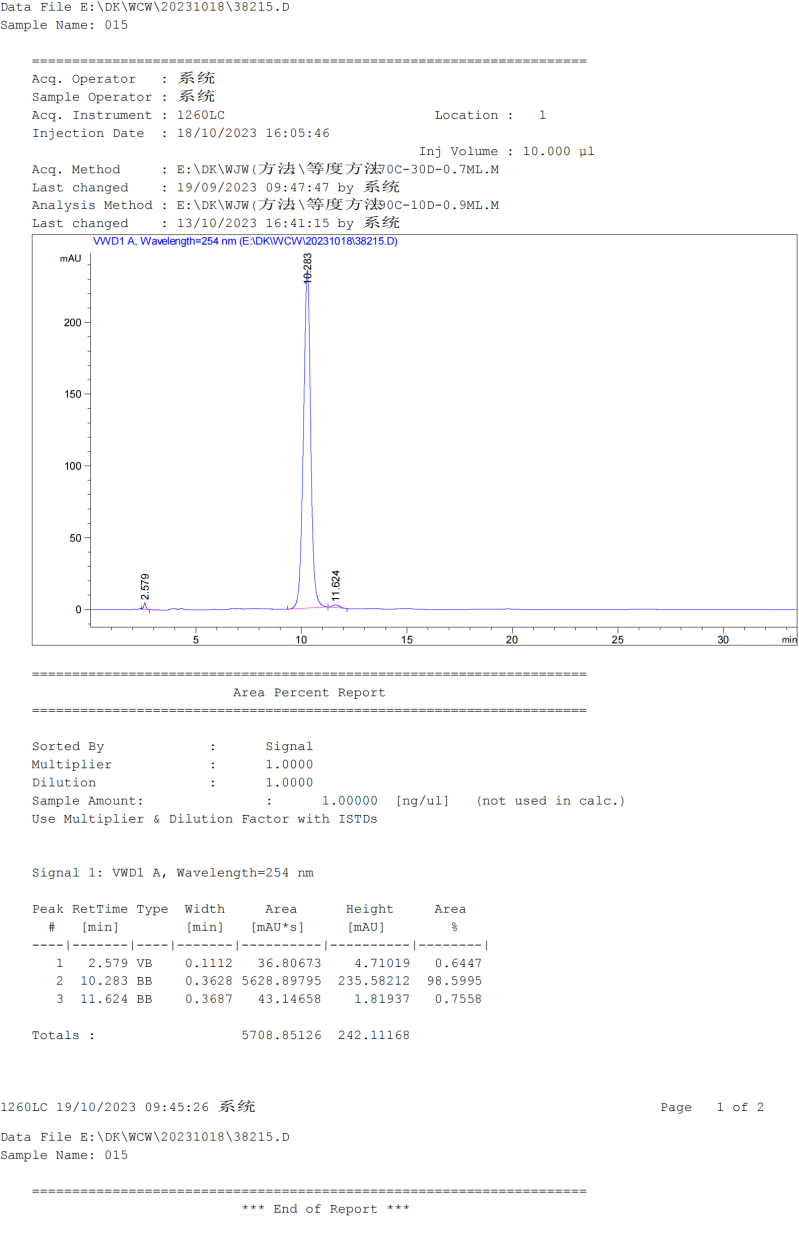
**

**^1^H and ^13^C NMR Spectra of compound 5b**


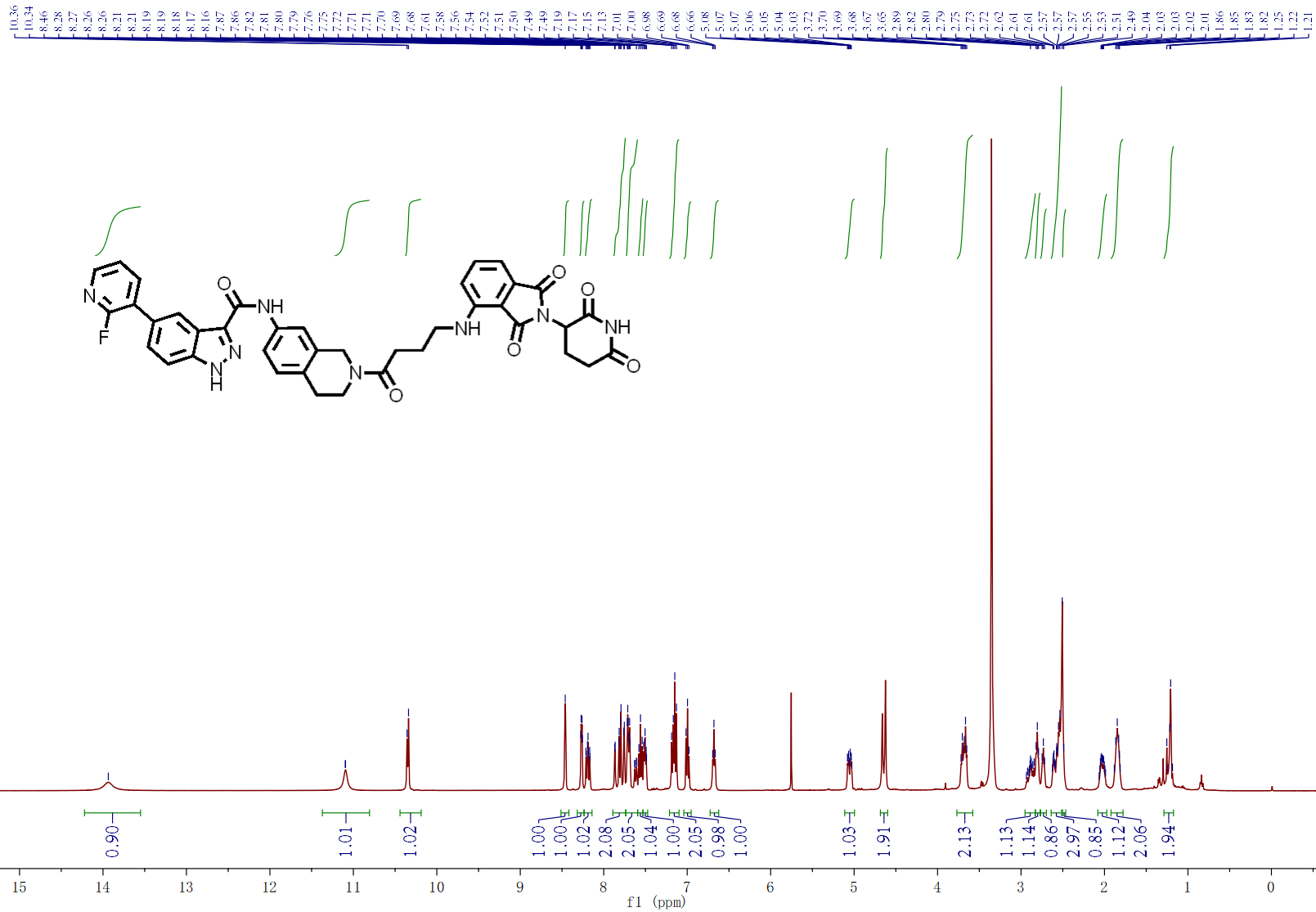

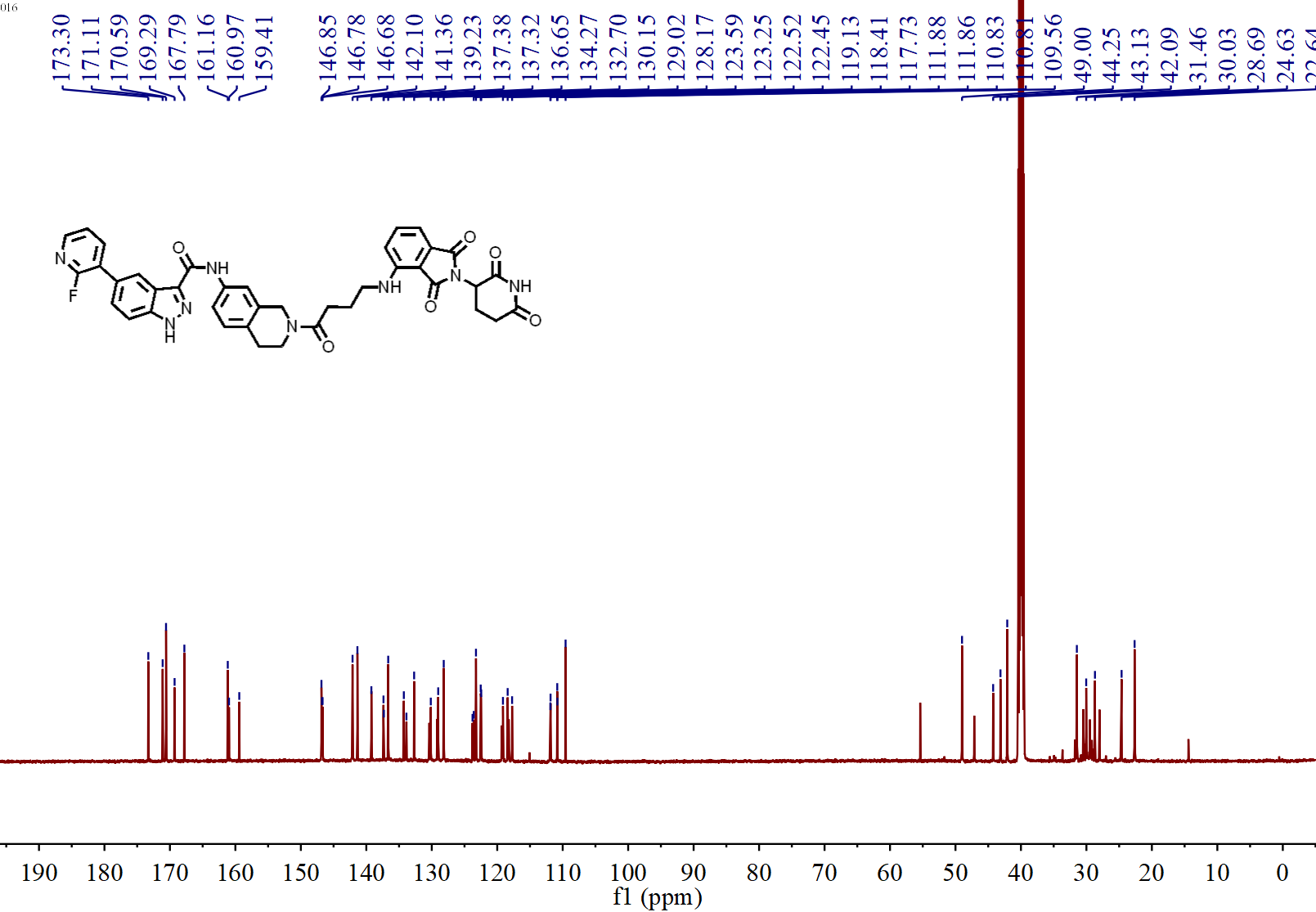


**HRMS Spectra of 5b**

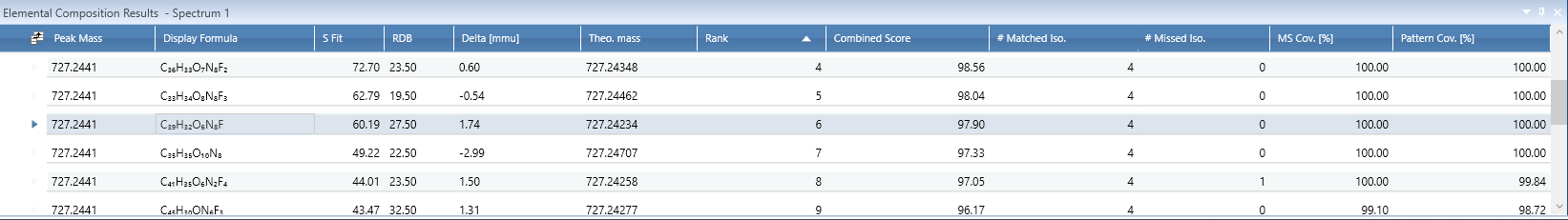


**HPLC Purity Data of 5b**

**
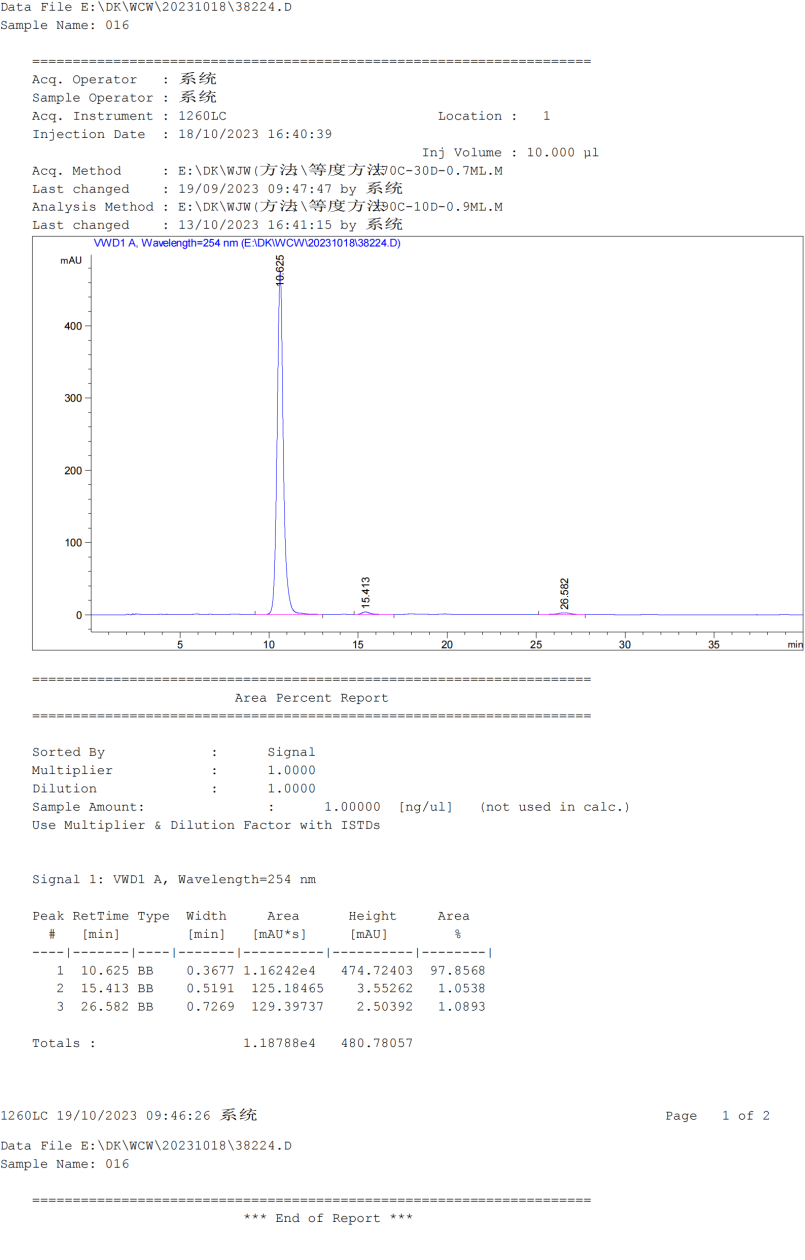
**

**^1^H and ^13^C NMR Spectra of compound 5c**


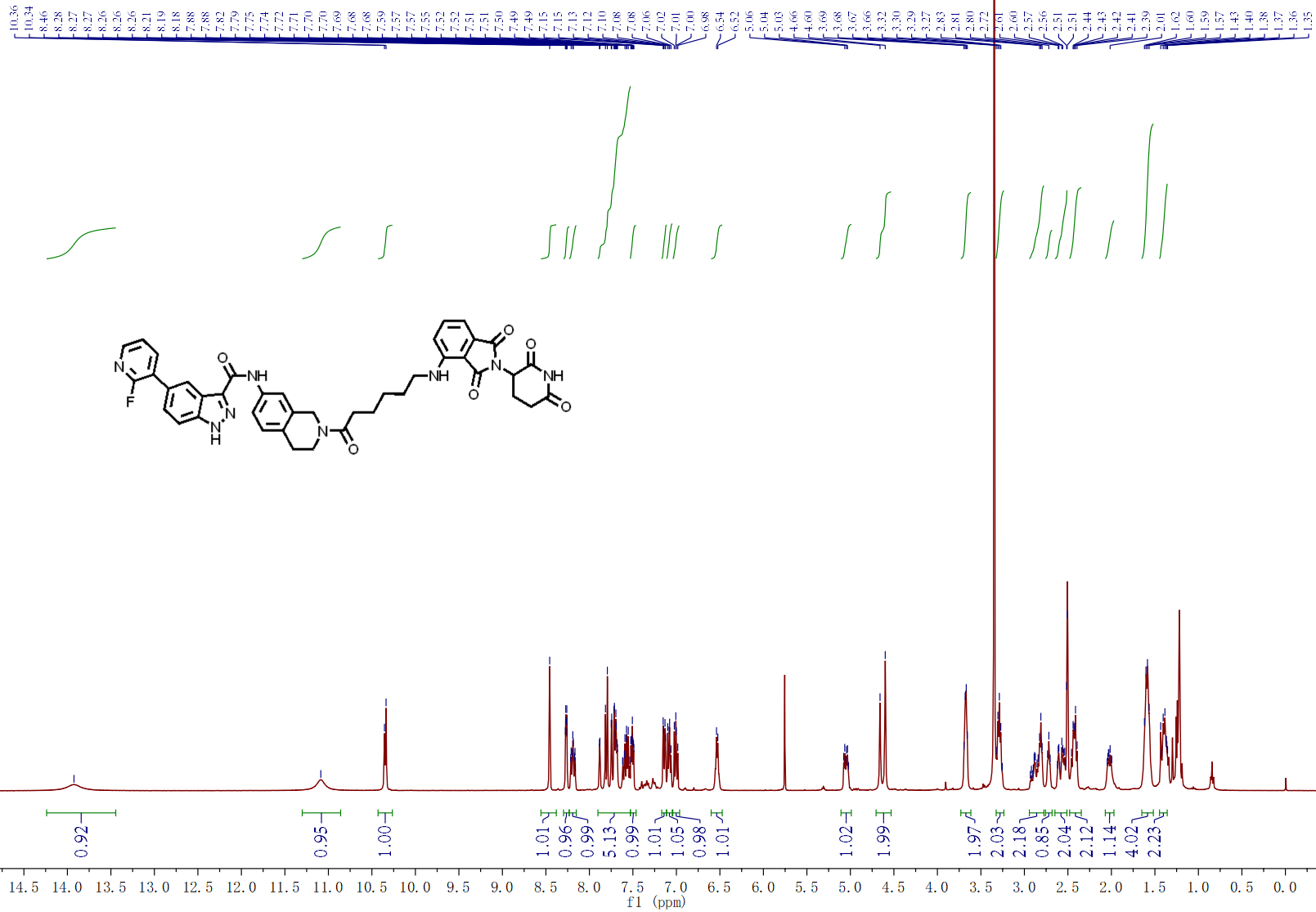

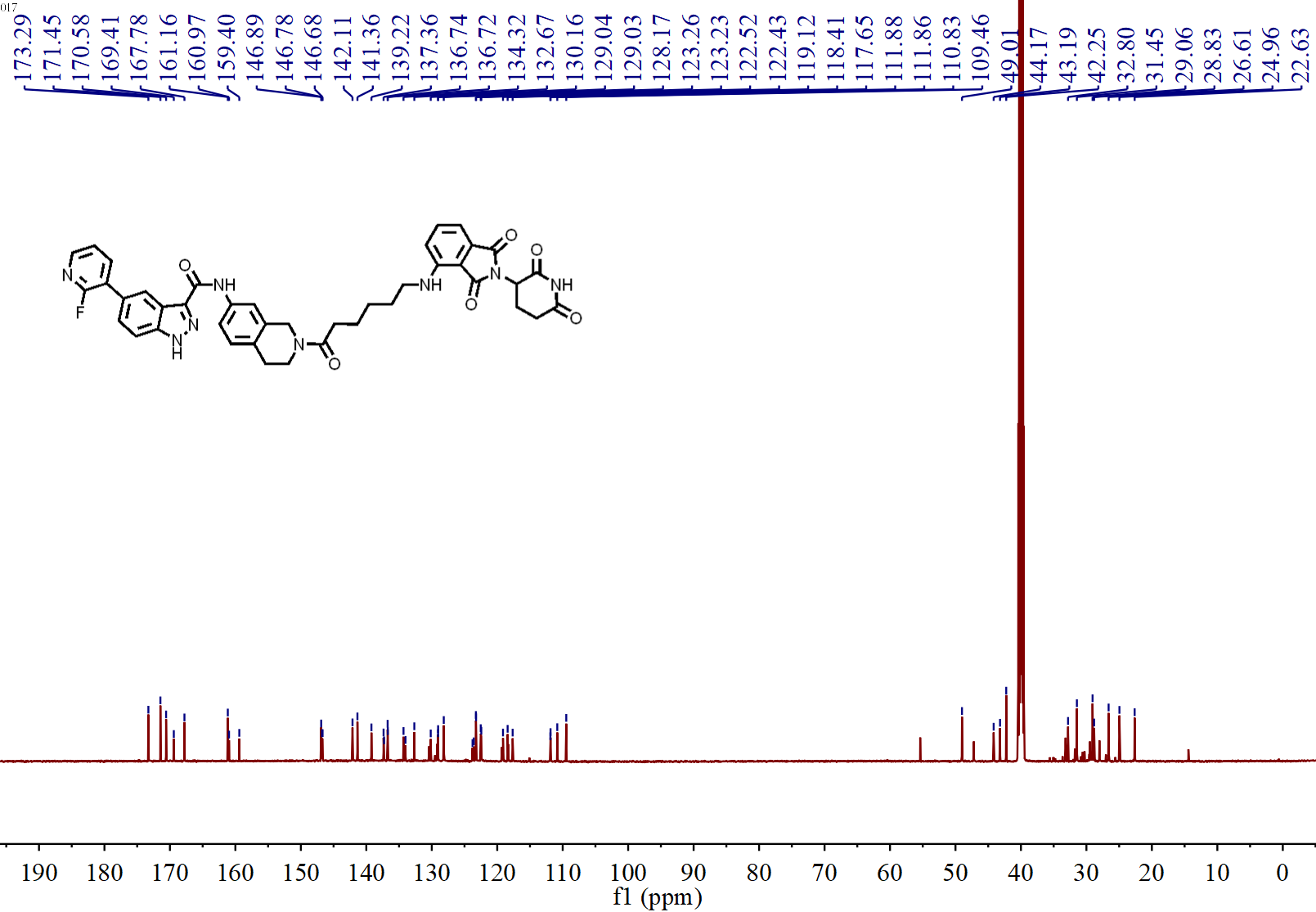


**HRMS Spectra of 5c**

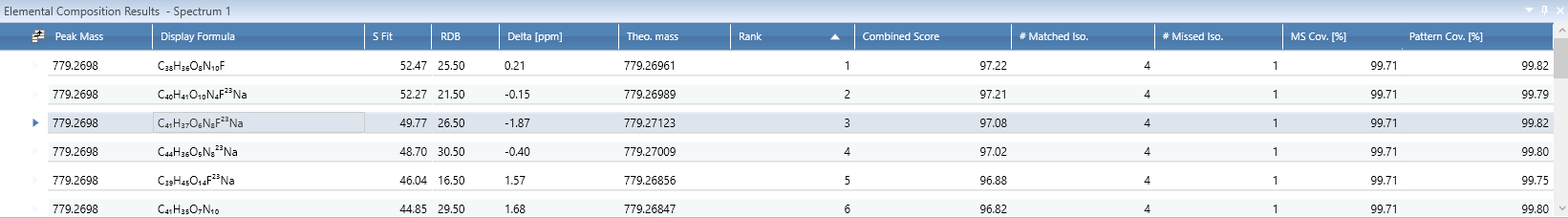


**HPLC Purity Data of 5c**


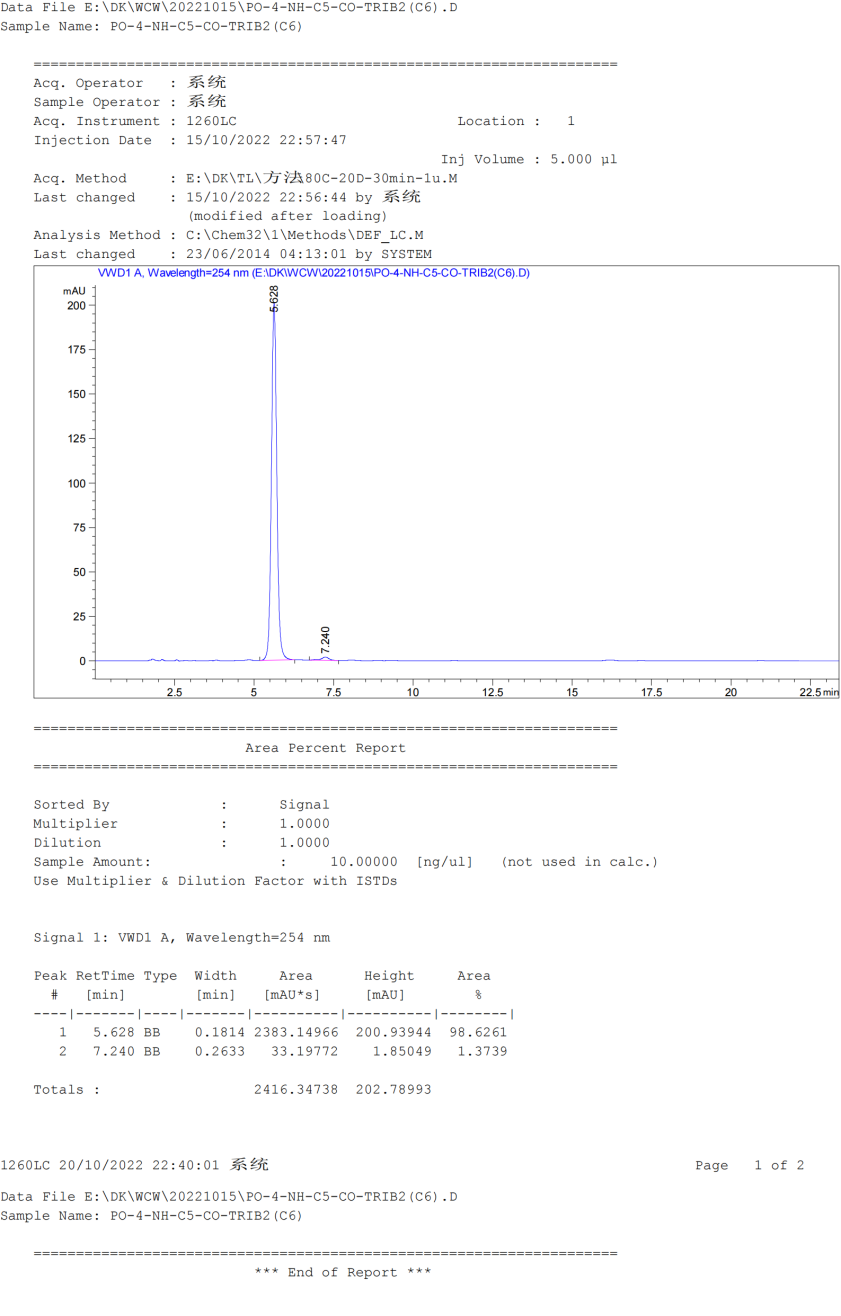


**^1^H and ^13^C NMR Spectra of compound 5d**


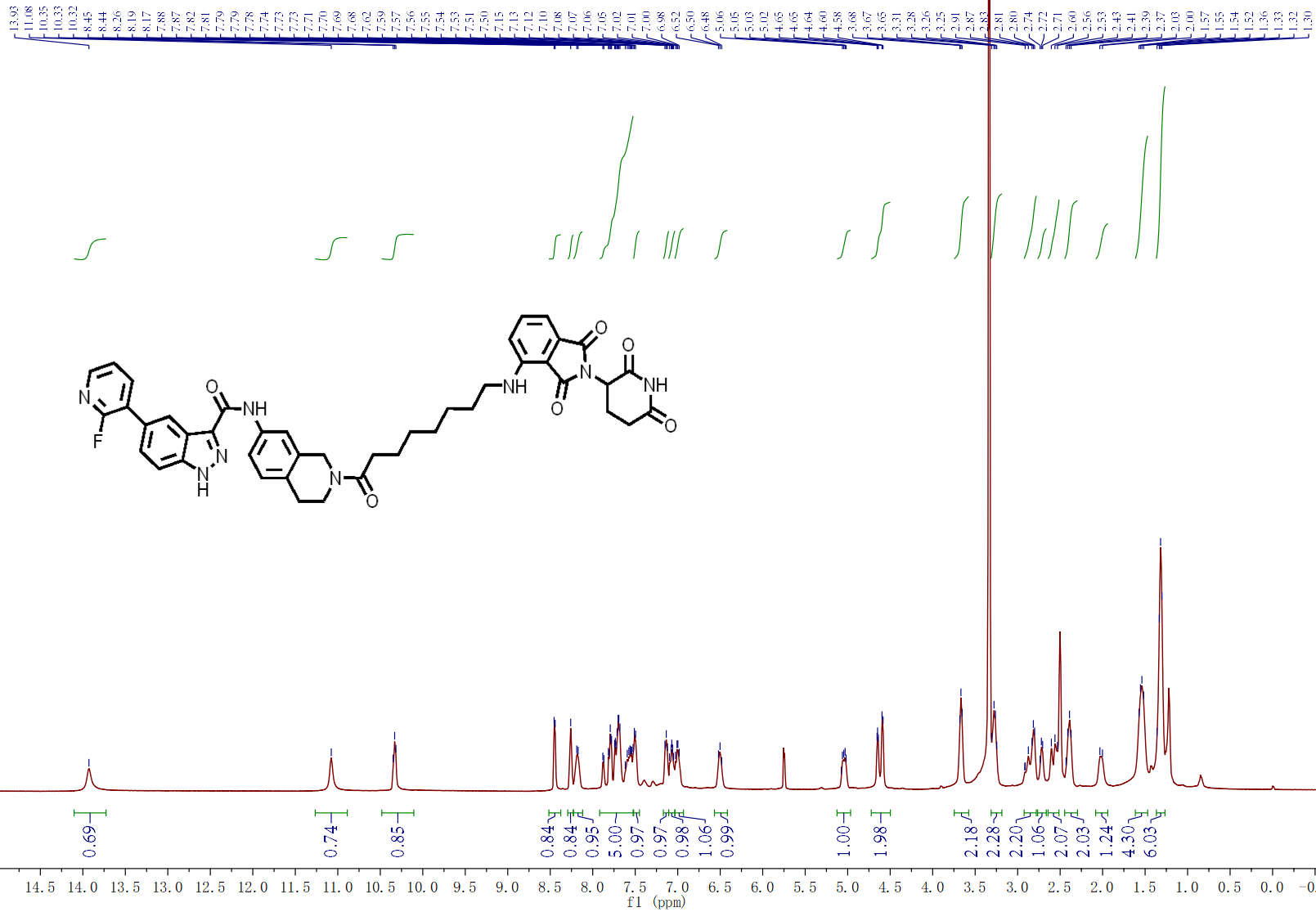

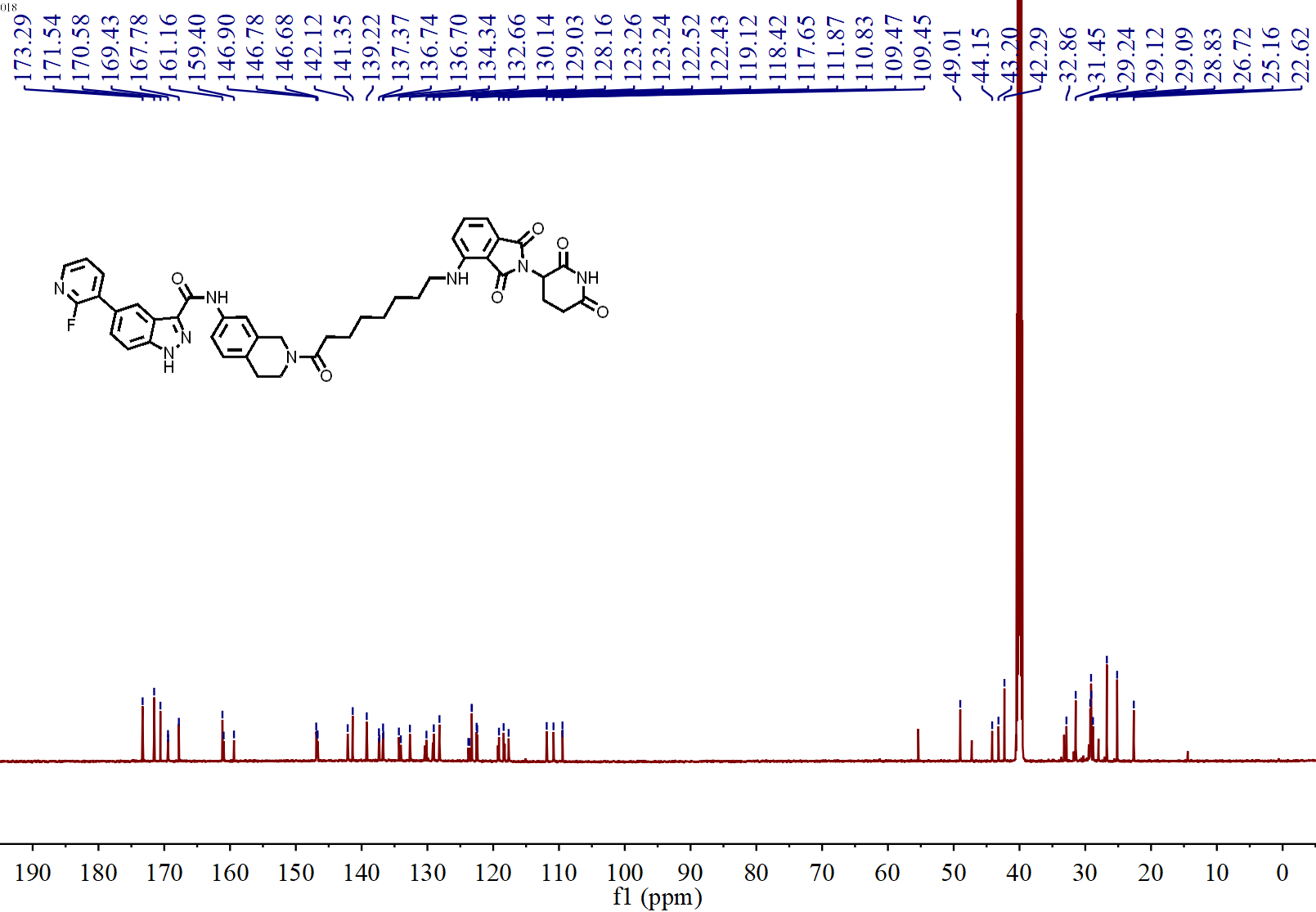


**HRMS Spectra of 5d**

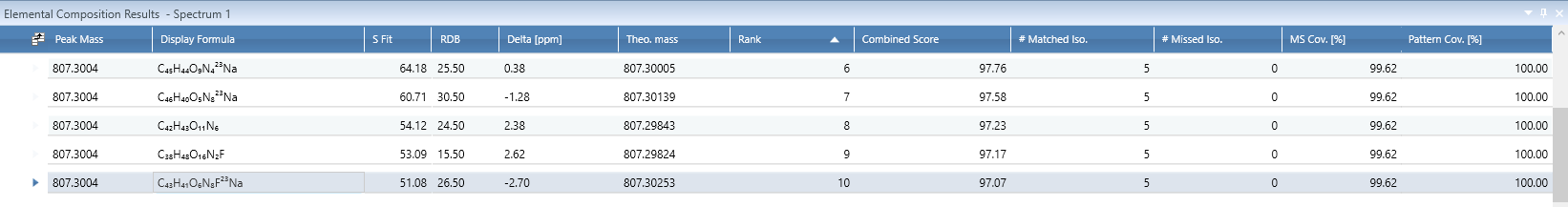


**HPLC Purity Data of 5d**


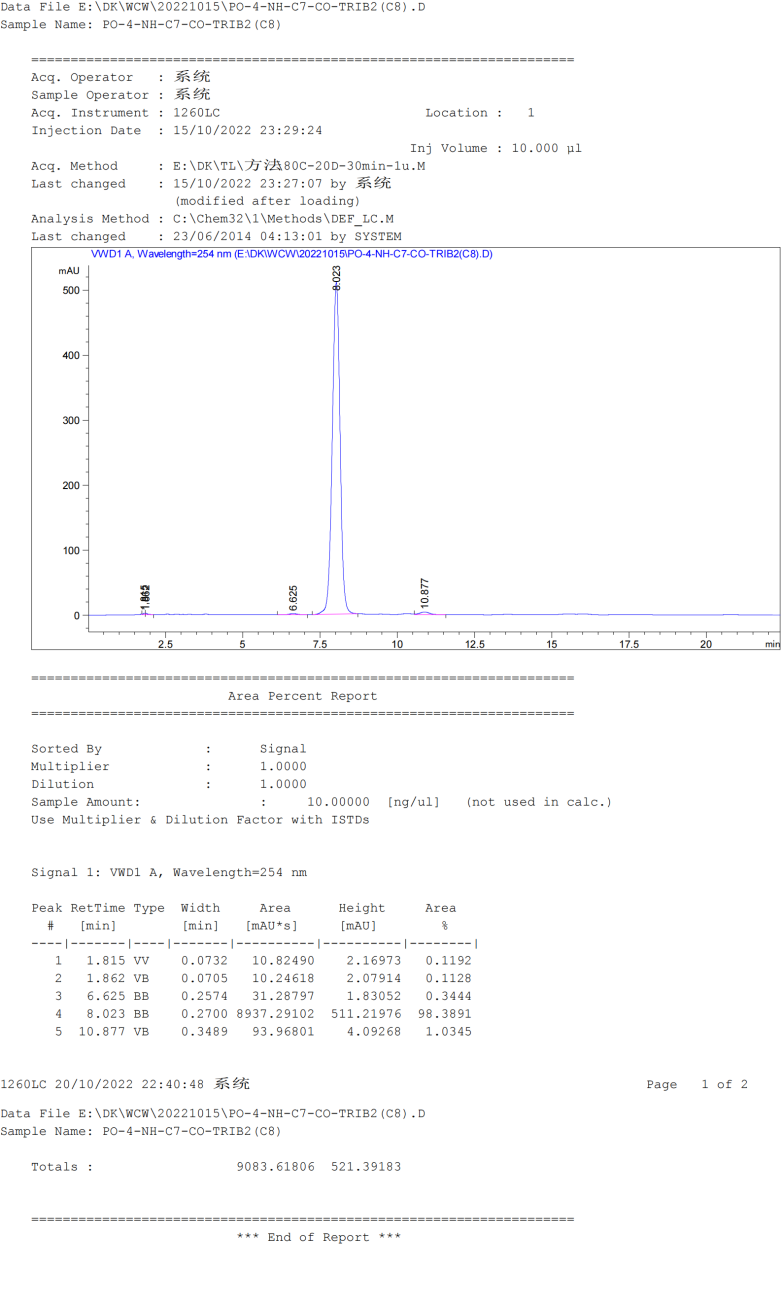


**^1^H and ^13^C NMR Spectra of compound 5e**


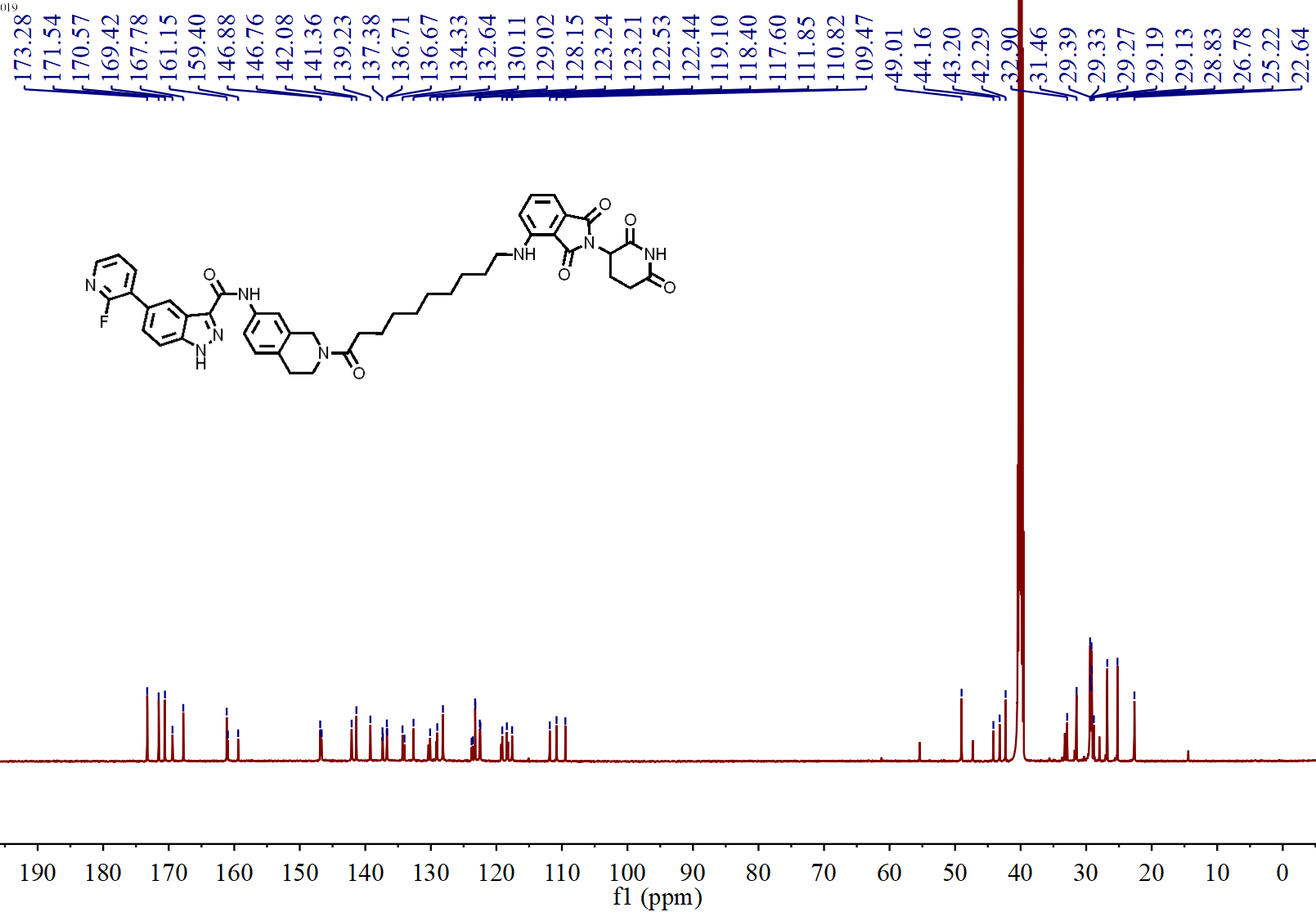

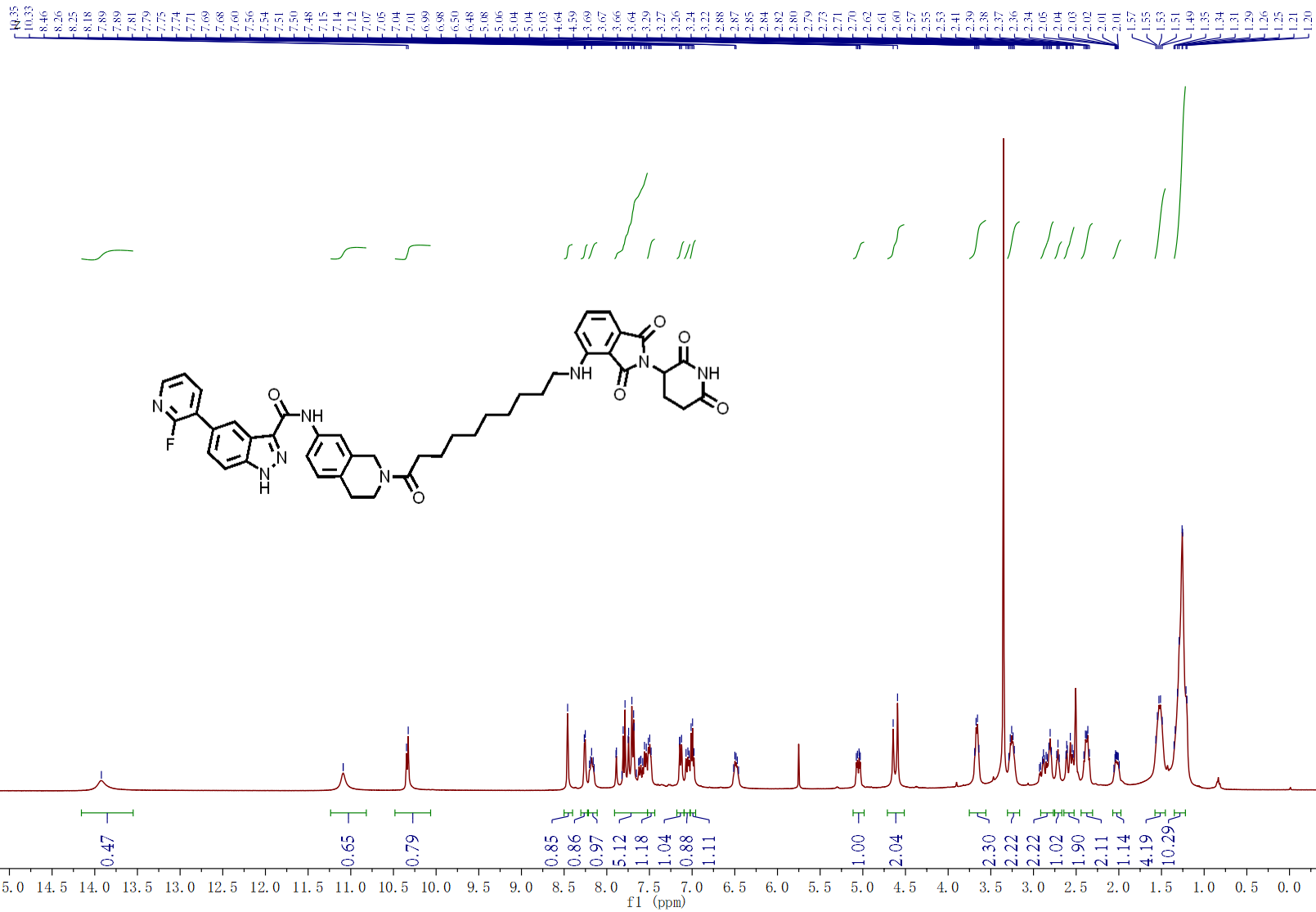


**HRMS Spectra of 5e**

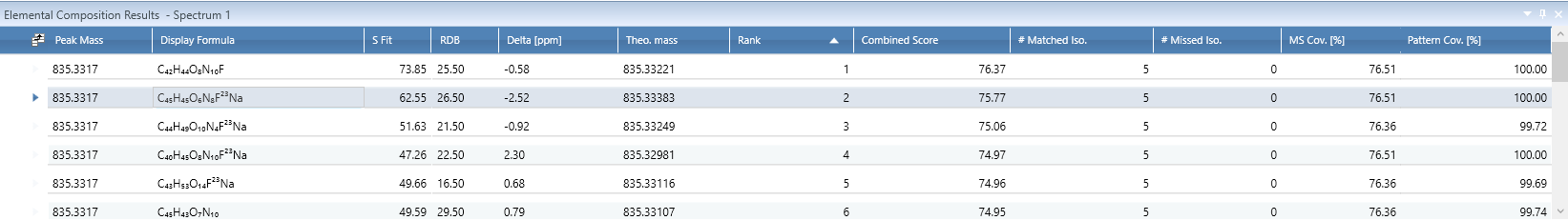


**HPLC Purity Data of 5e**


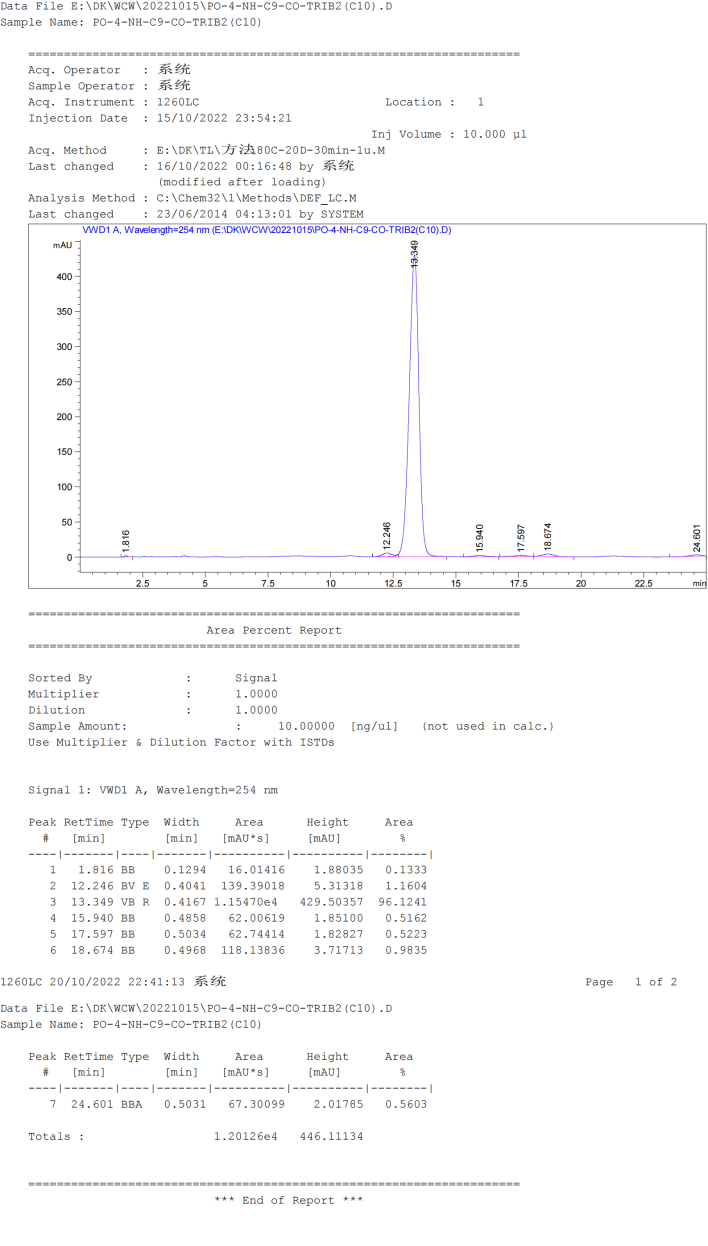


**^1^H and ^13^C NMR Spectra of compound 5f**


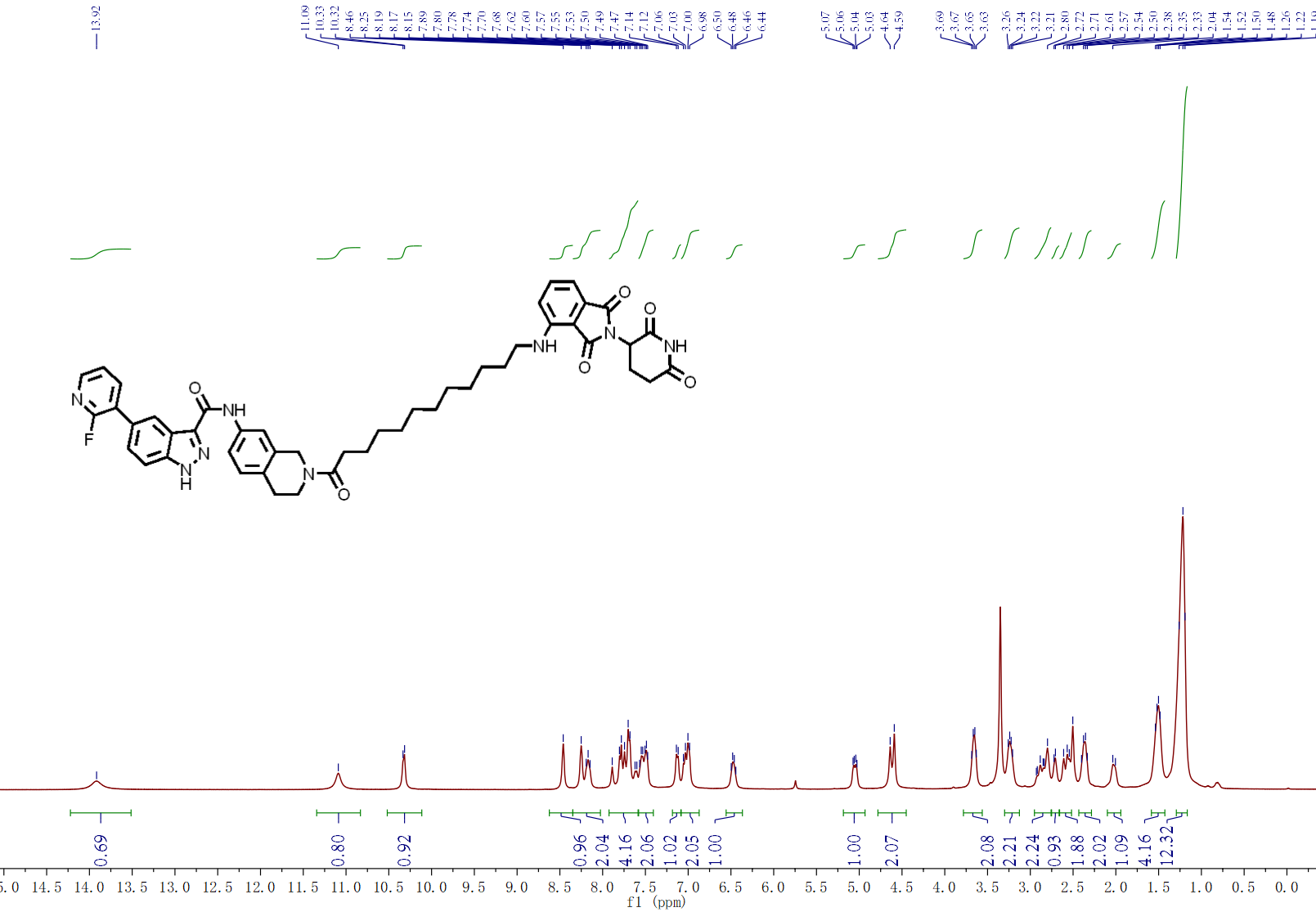

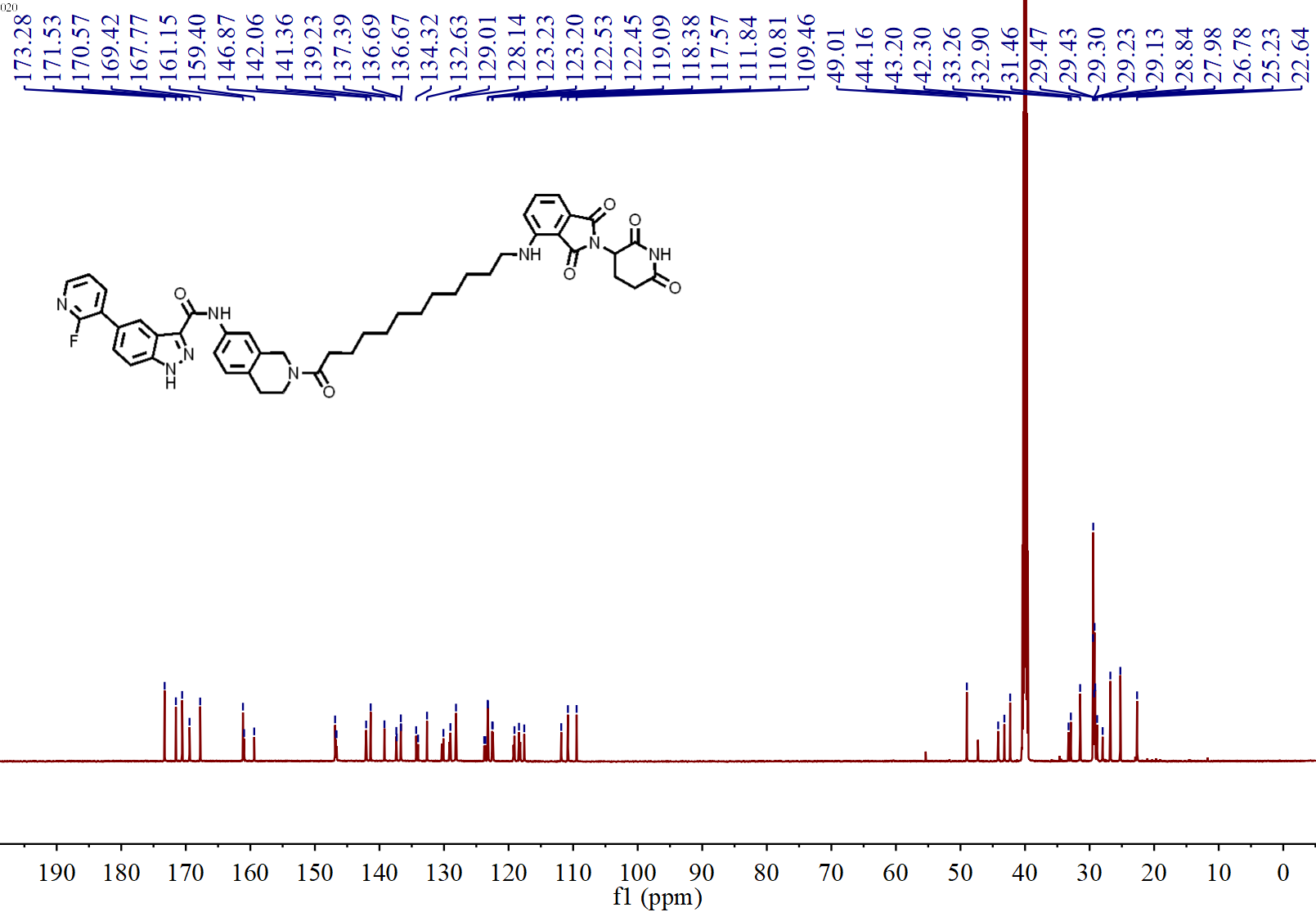


**HRMS Spectra of 5f**

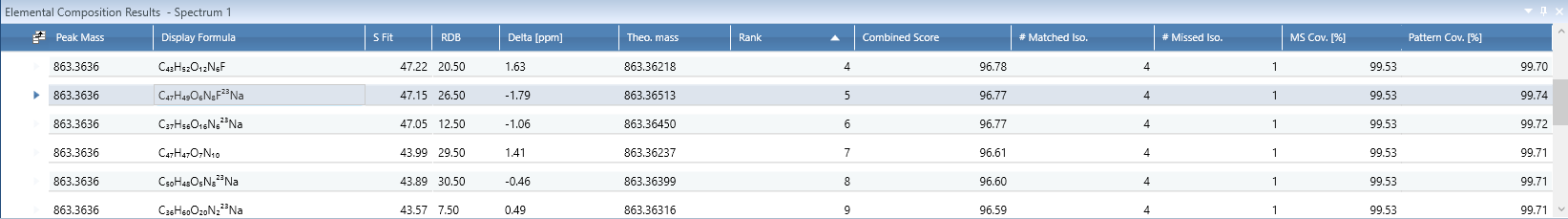


**HPLC Purity Data of 5f**


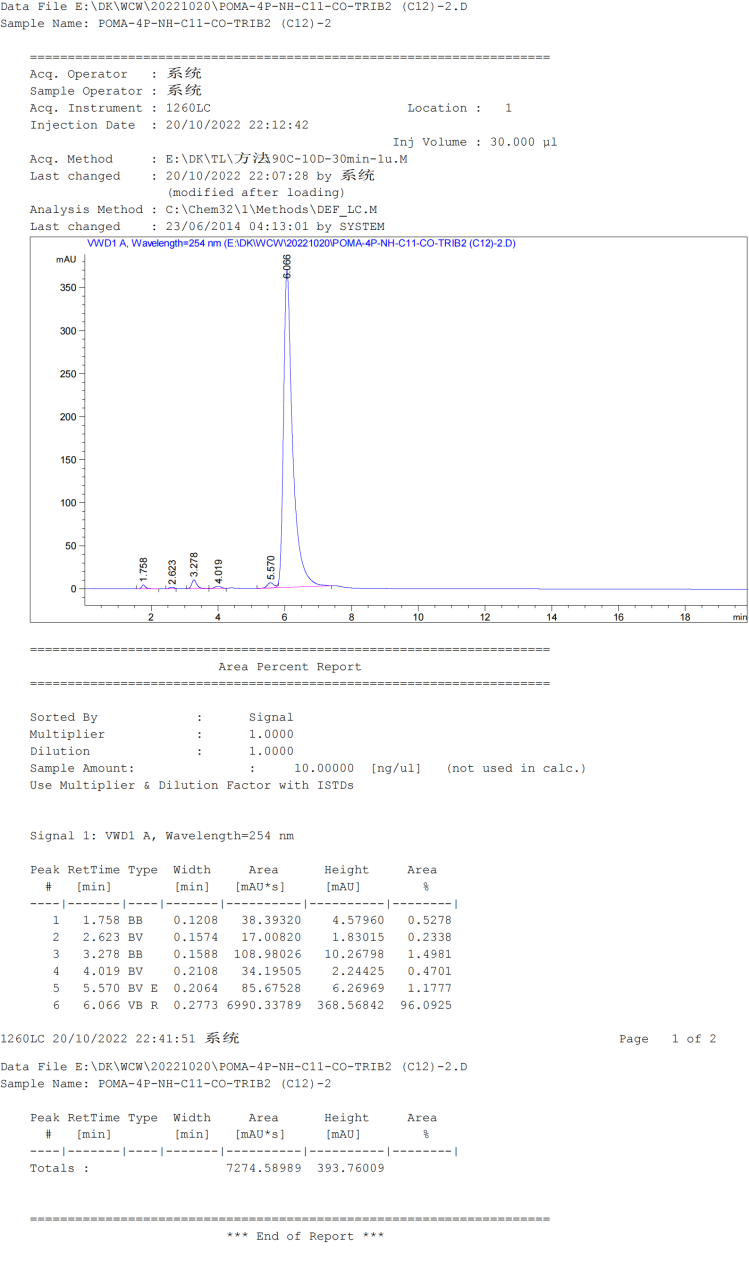


**^1^H and ^13^C NMR Spectra of compound 5g**


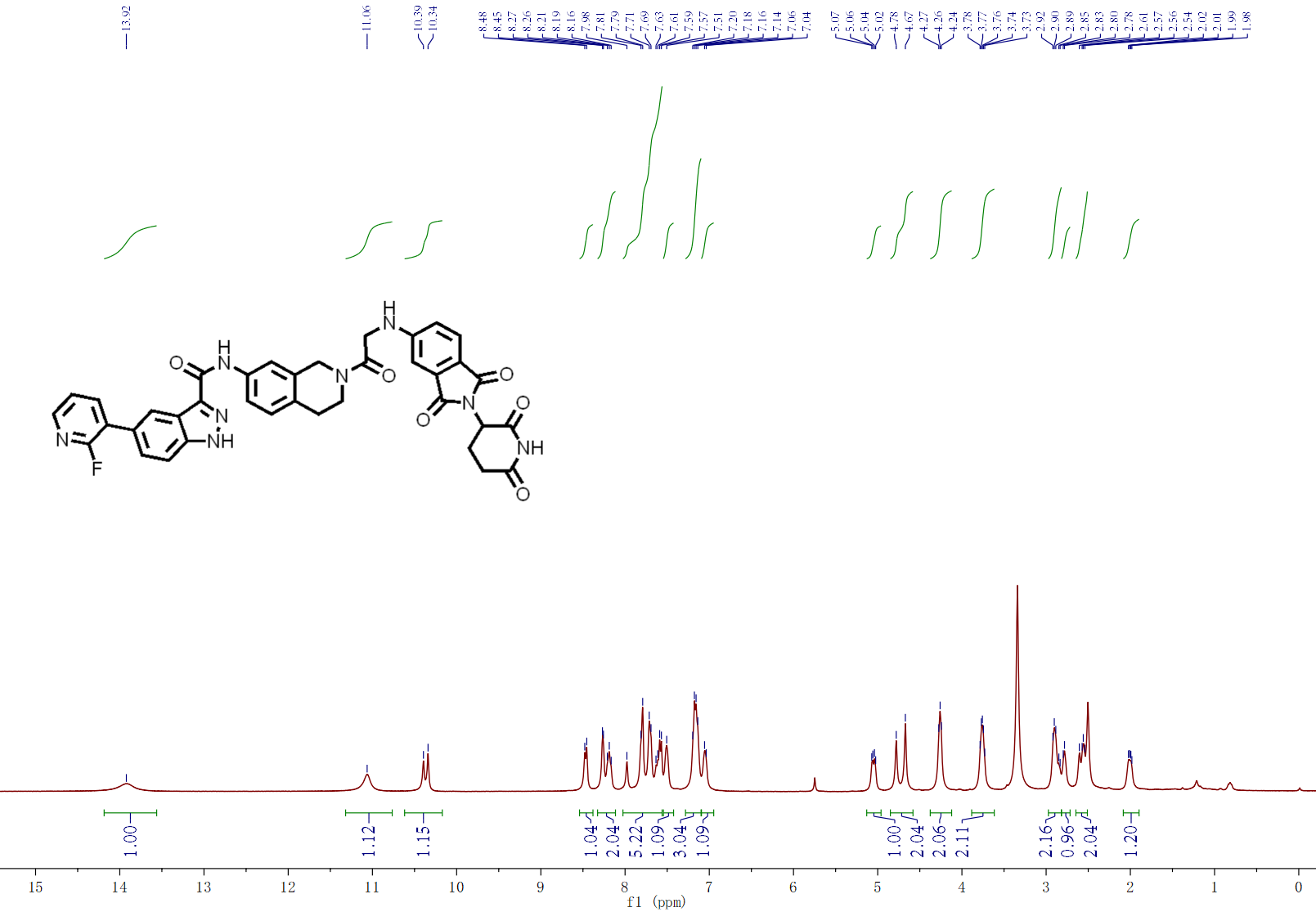

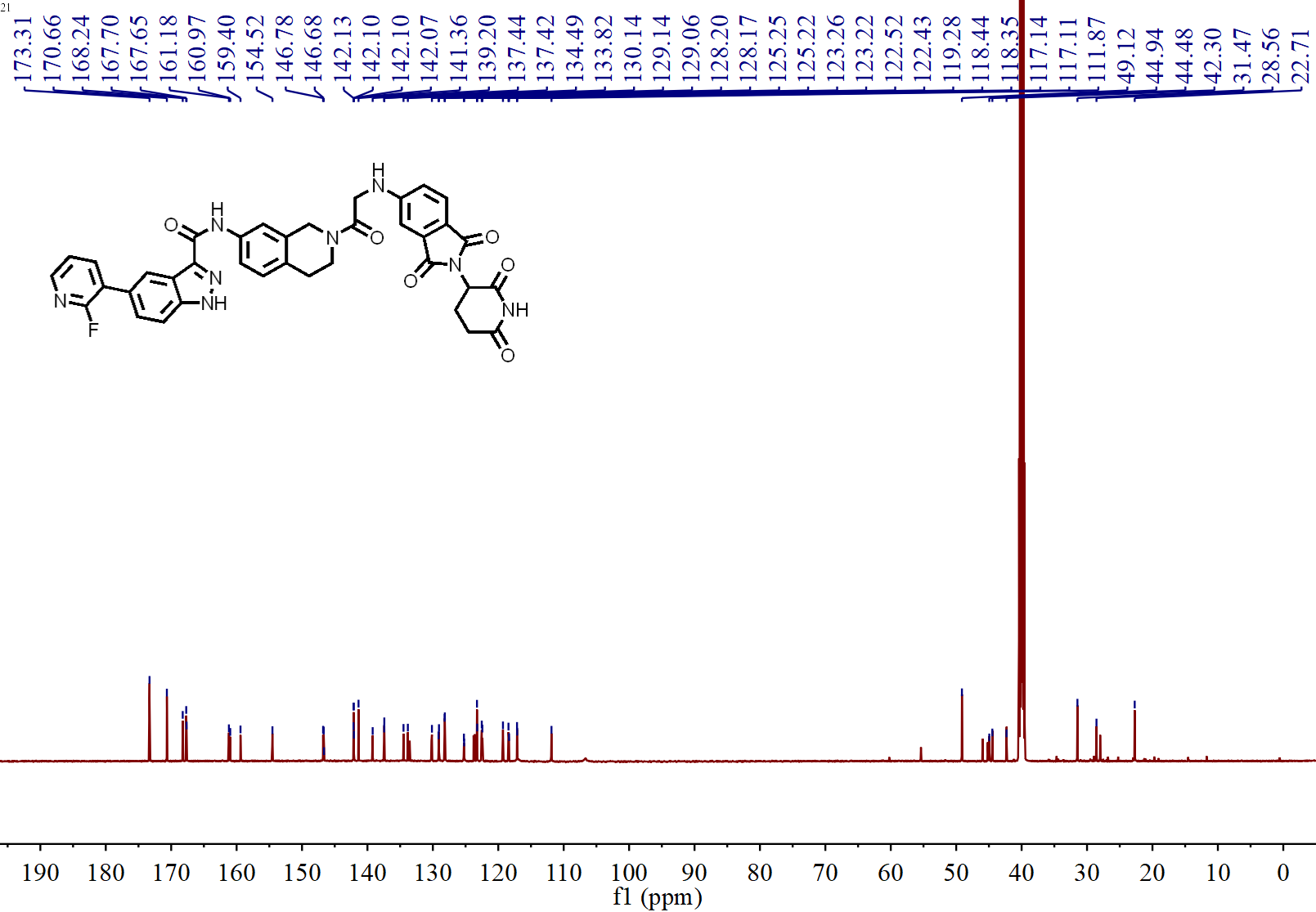


**HRMS Spectra of 5g**


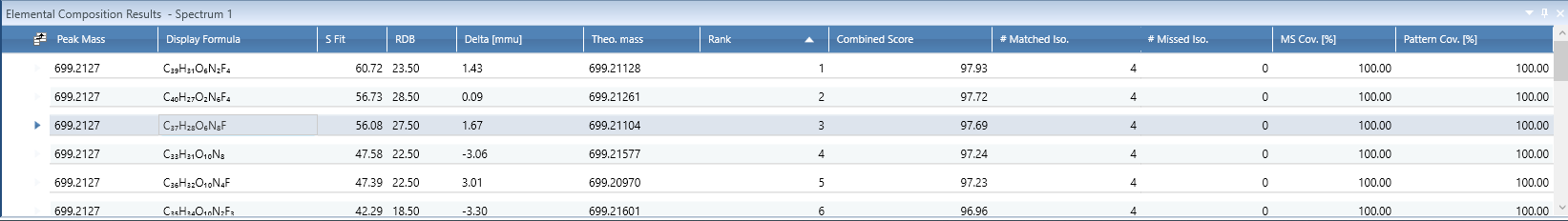


**HPLC Purity Data of 5g**

**
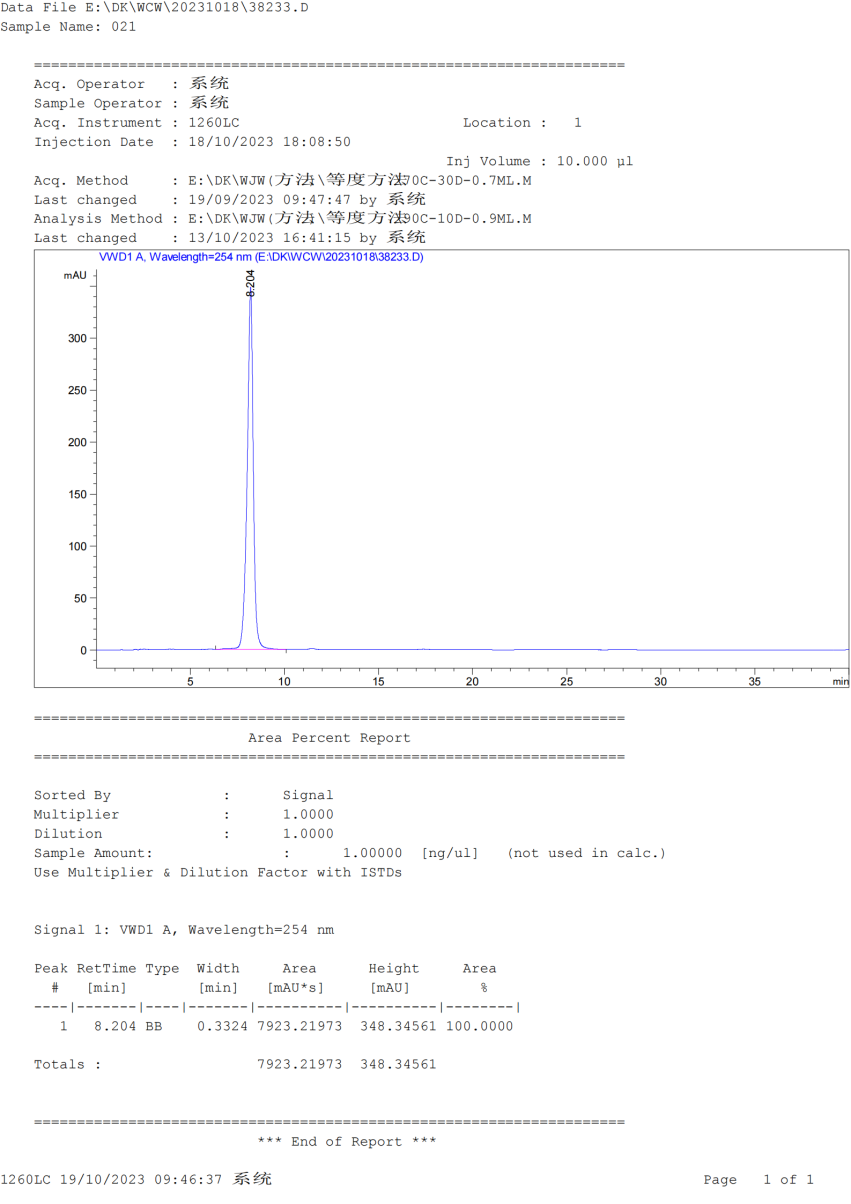
**

**^1^H and ^13^C NMR Spectra of compound 5h**
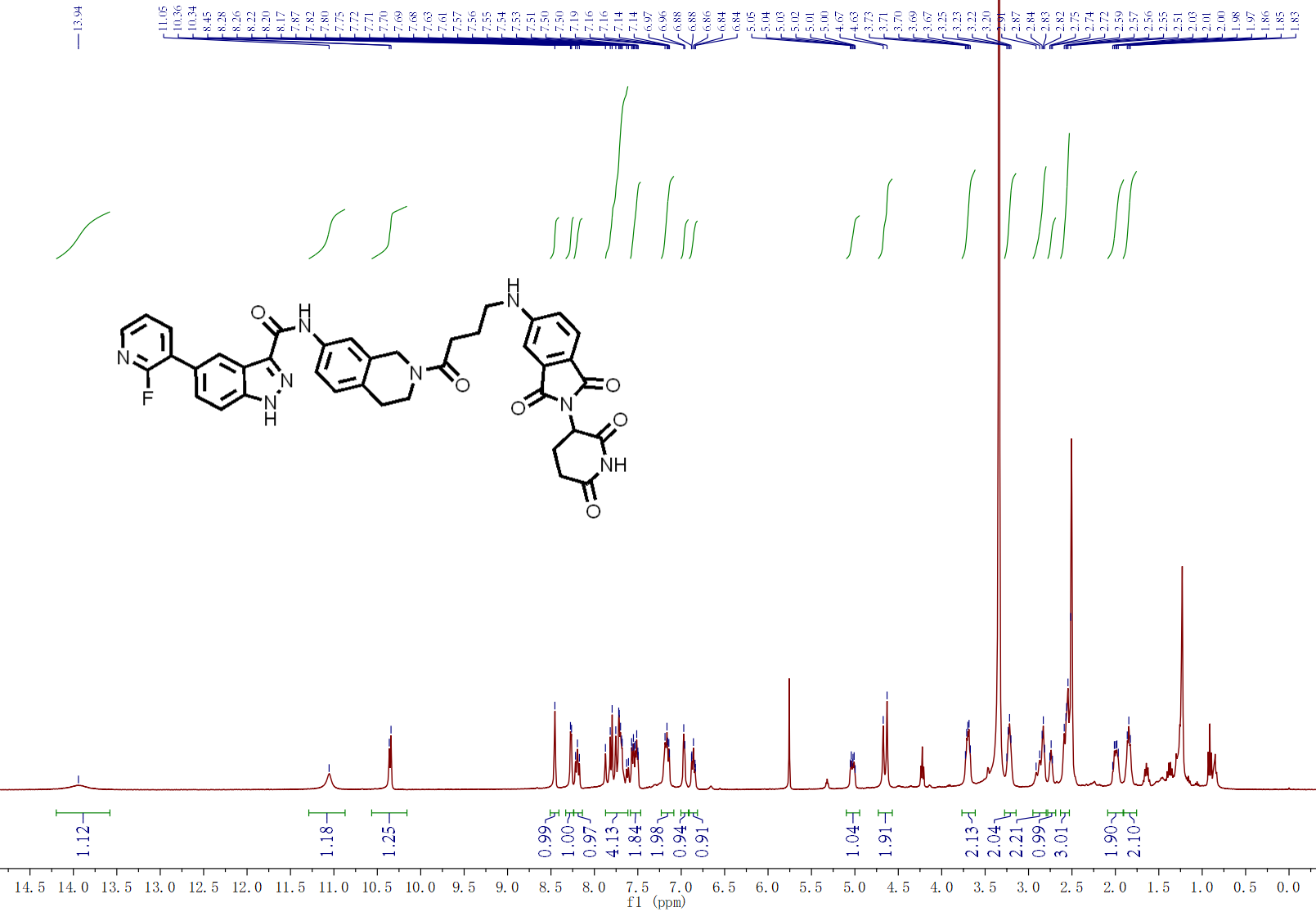

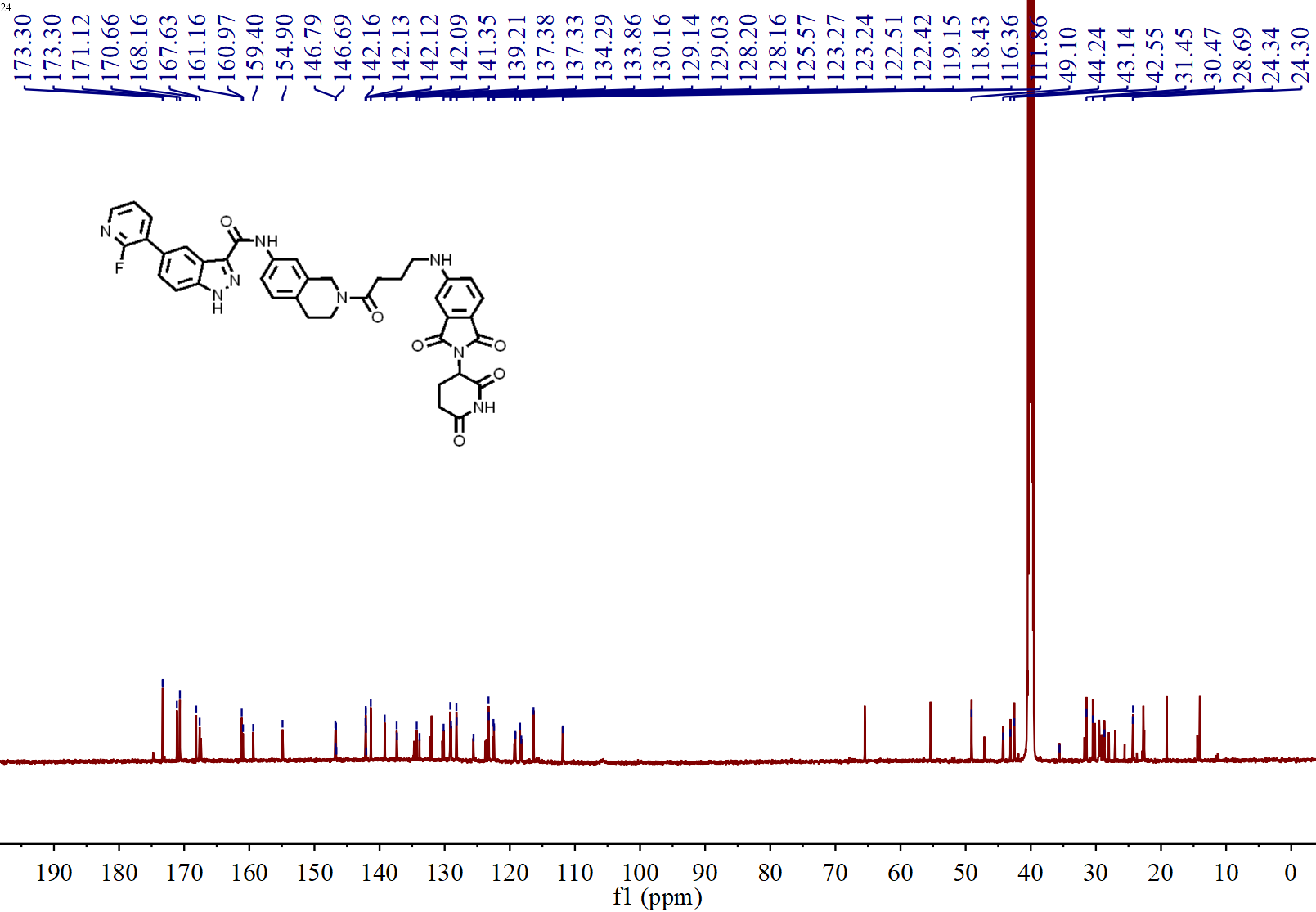


**HRMS Spectra of 5h**

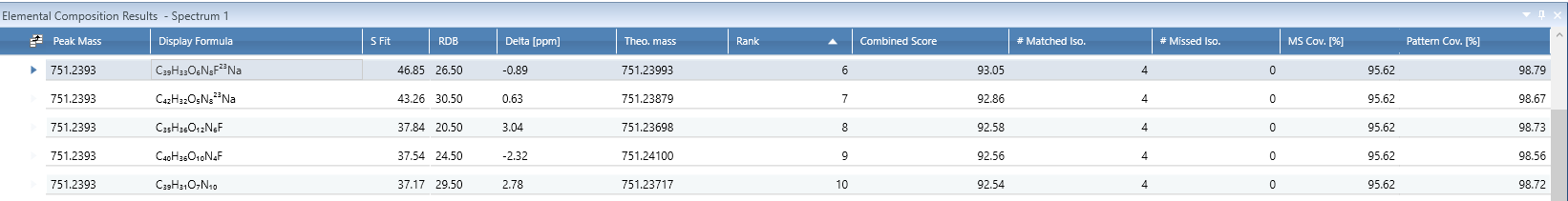


**HPLC Purity Data of 5h**


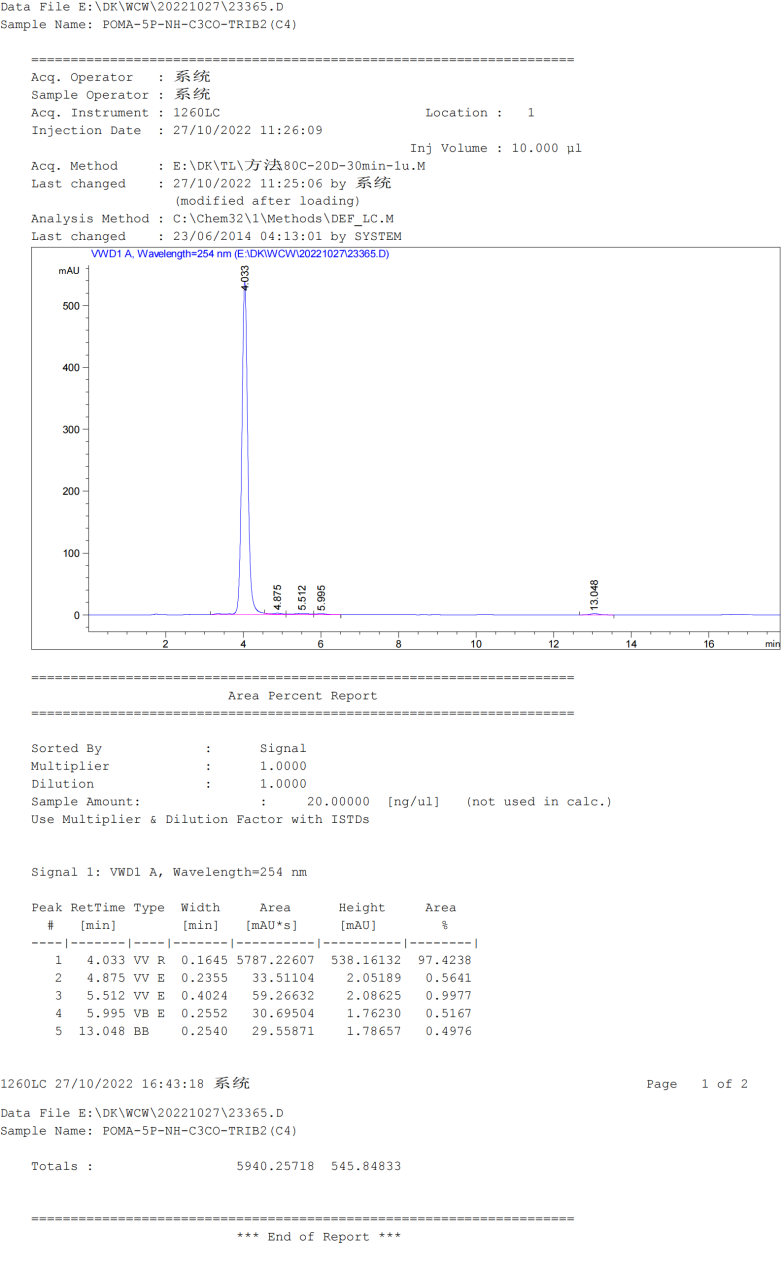


**^1^H and ^13^C NMR Spectra of compound 5i**


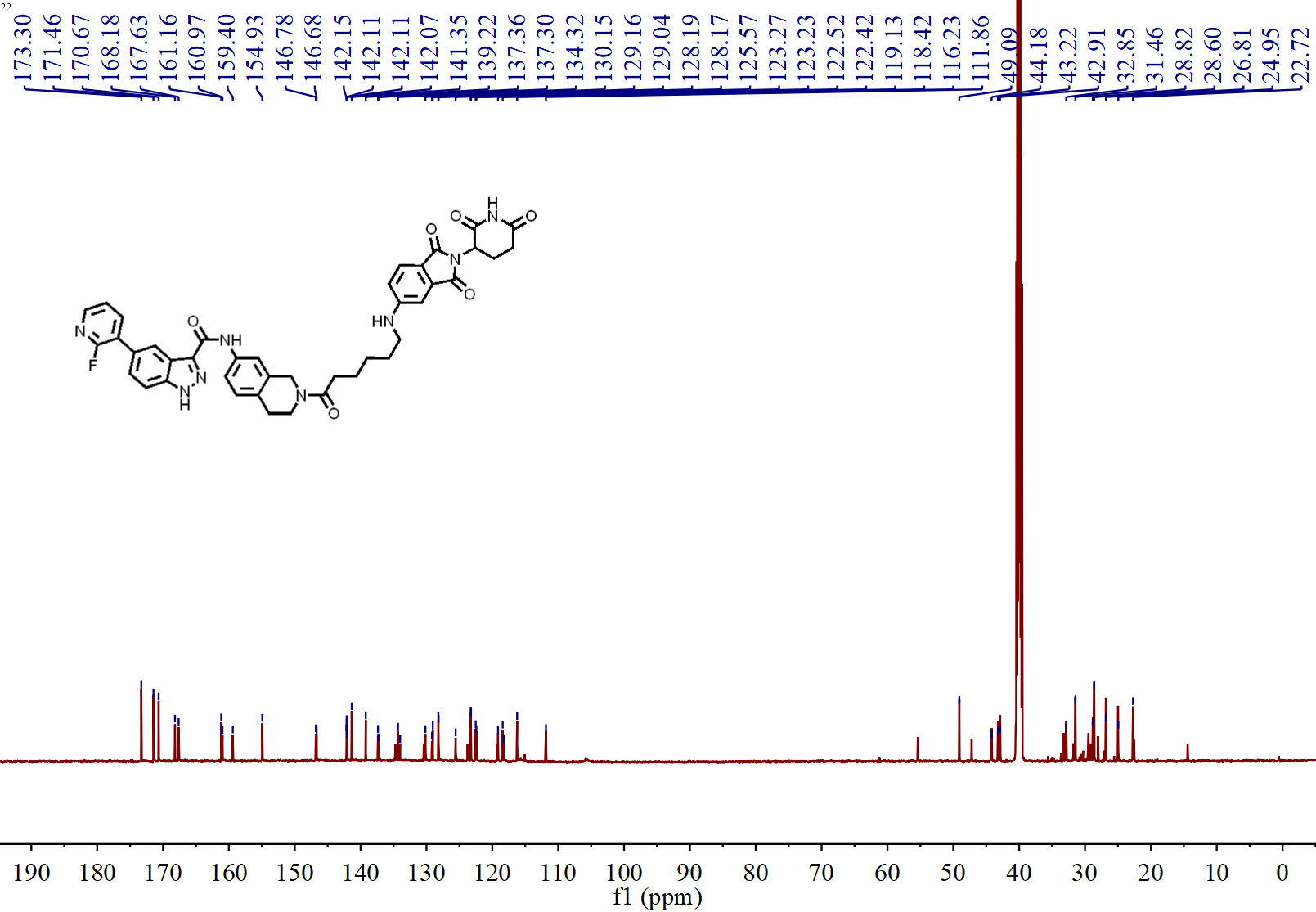

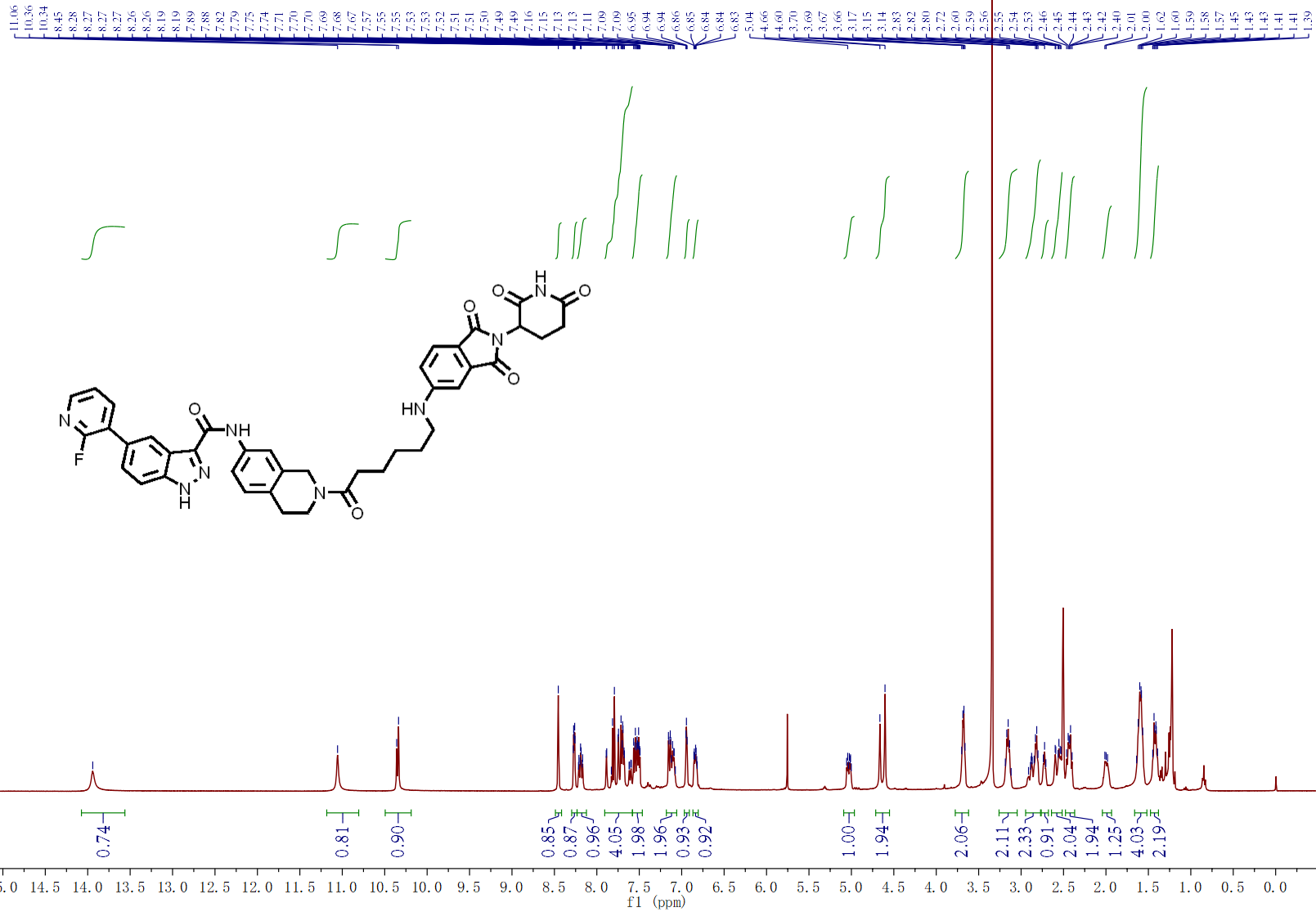


**HRMS Spectra of 5i**


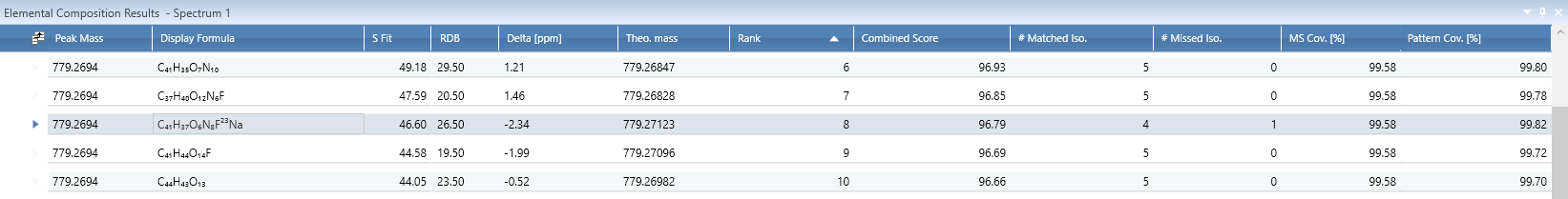


**HPLC Purity Data of 5i**


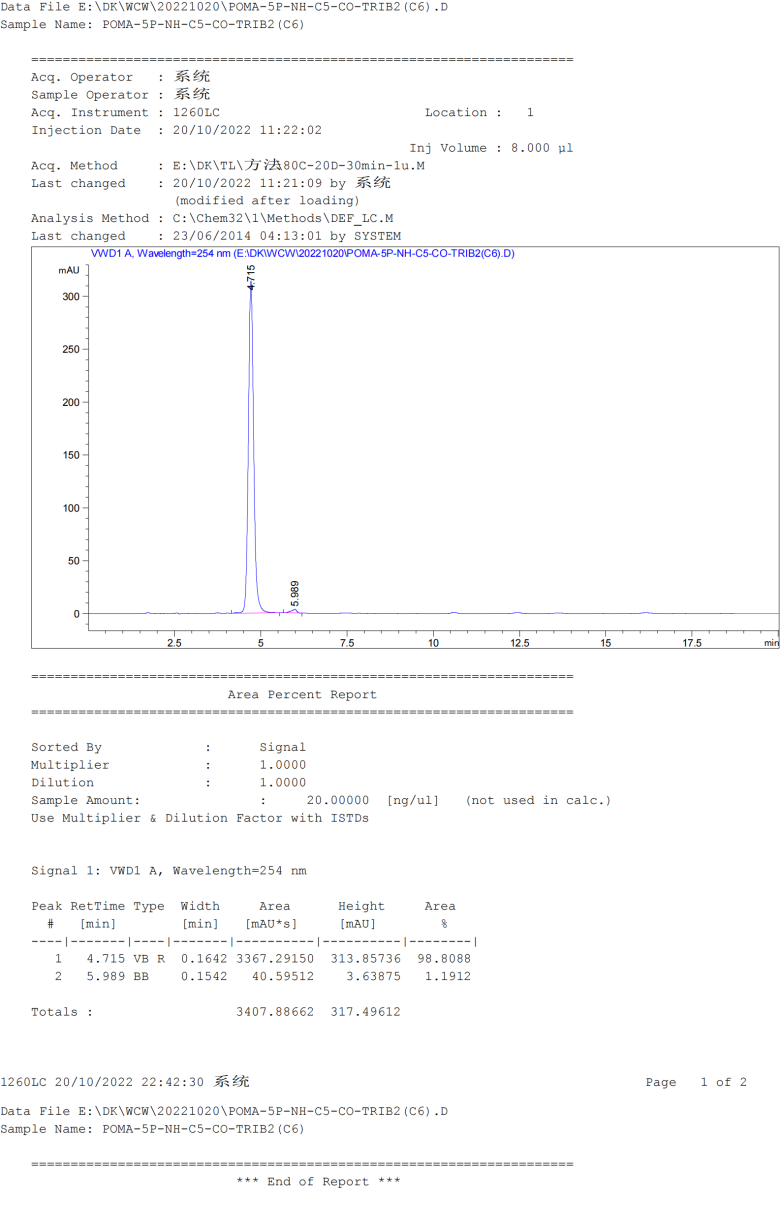


**^1^H and ^13^C NMR Spectra of compound 5j**


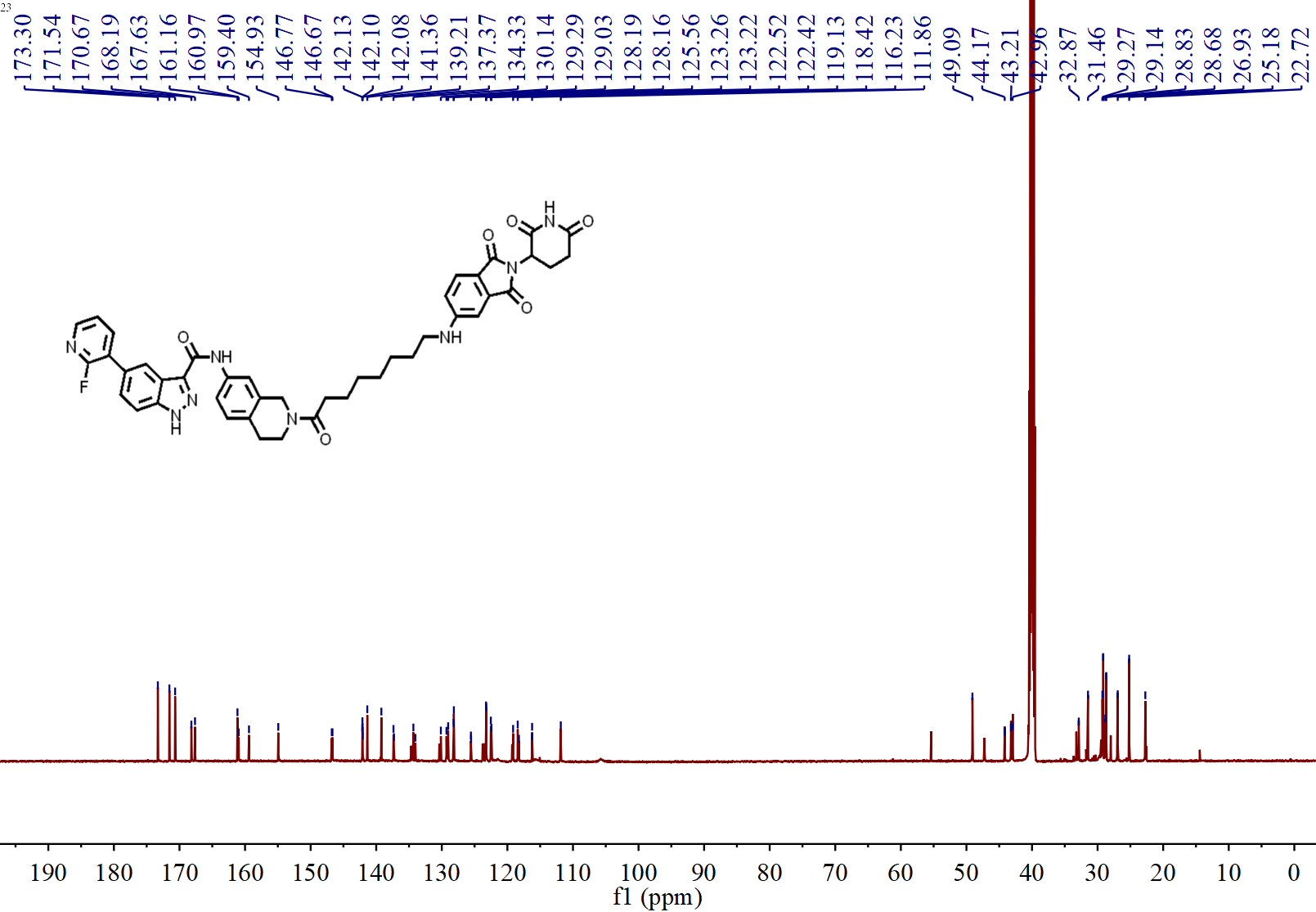


**HRMS Spectra of 5j**

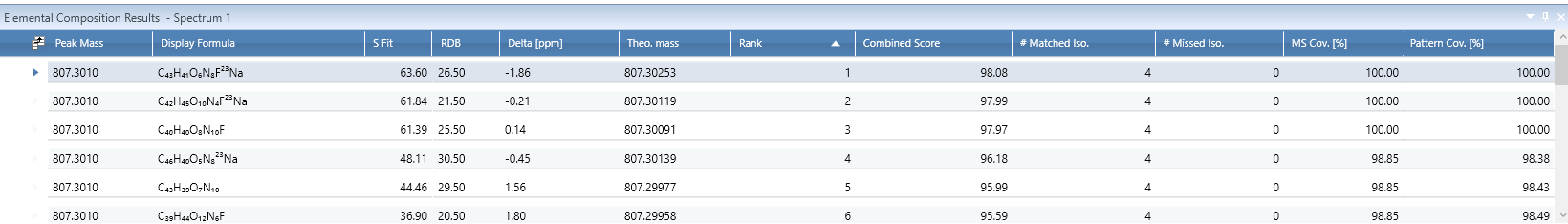


**HPLC Purity Data of 5j**


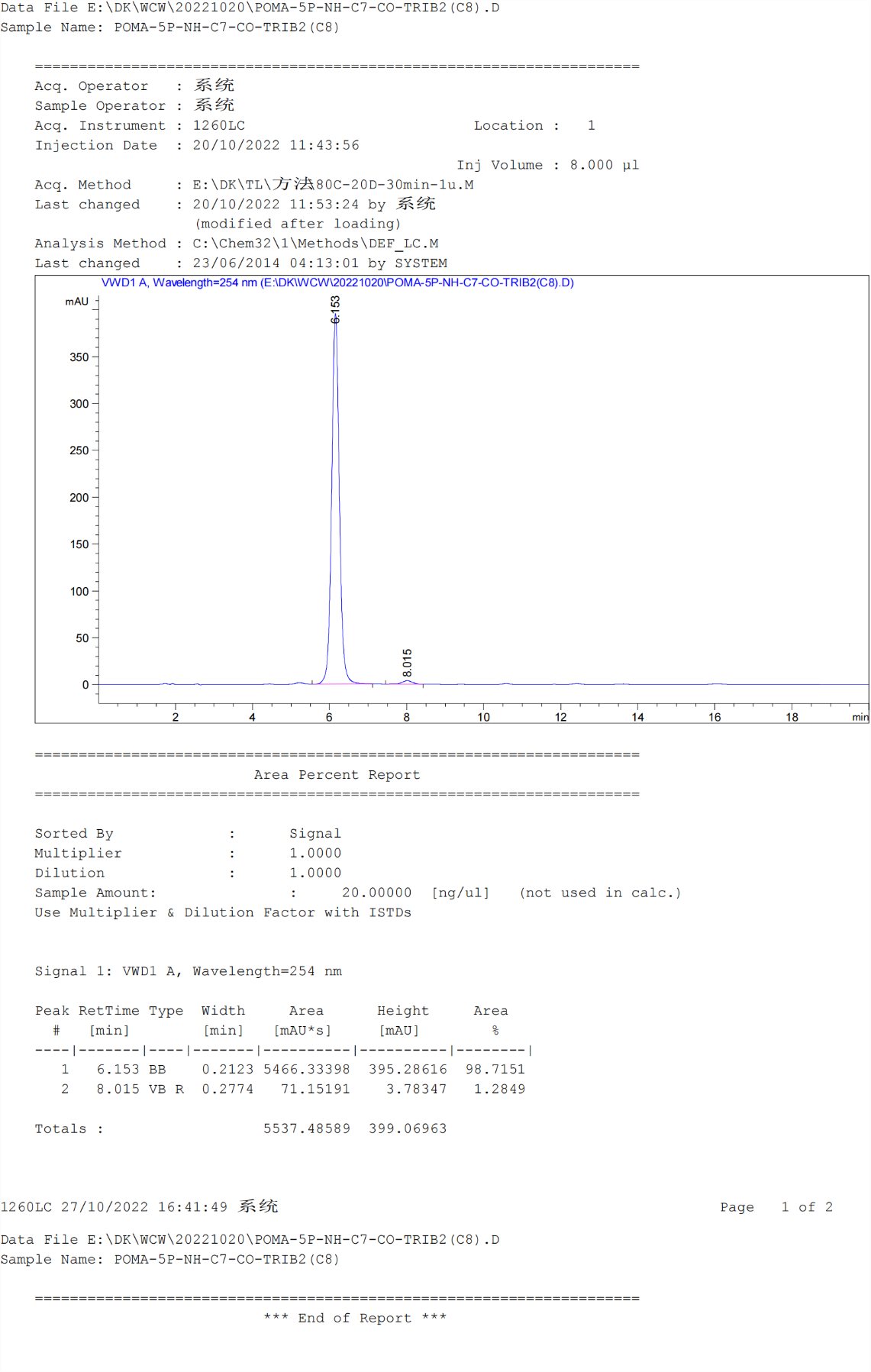


**^1^H and ^13^C NMR Spectra of compound 5k**


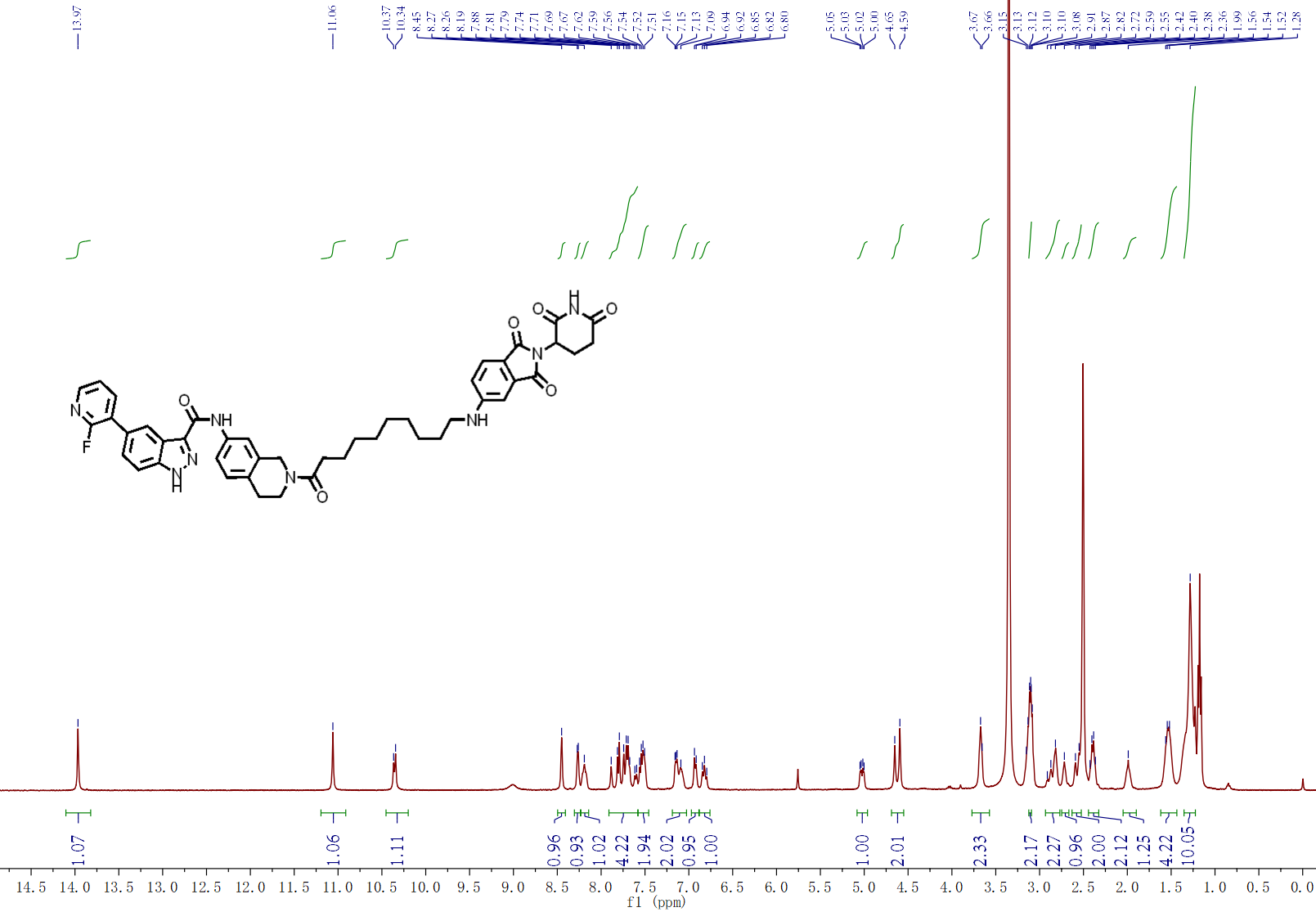

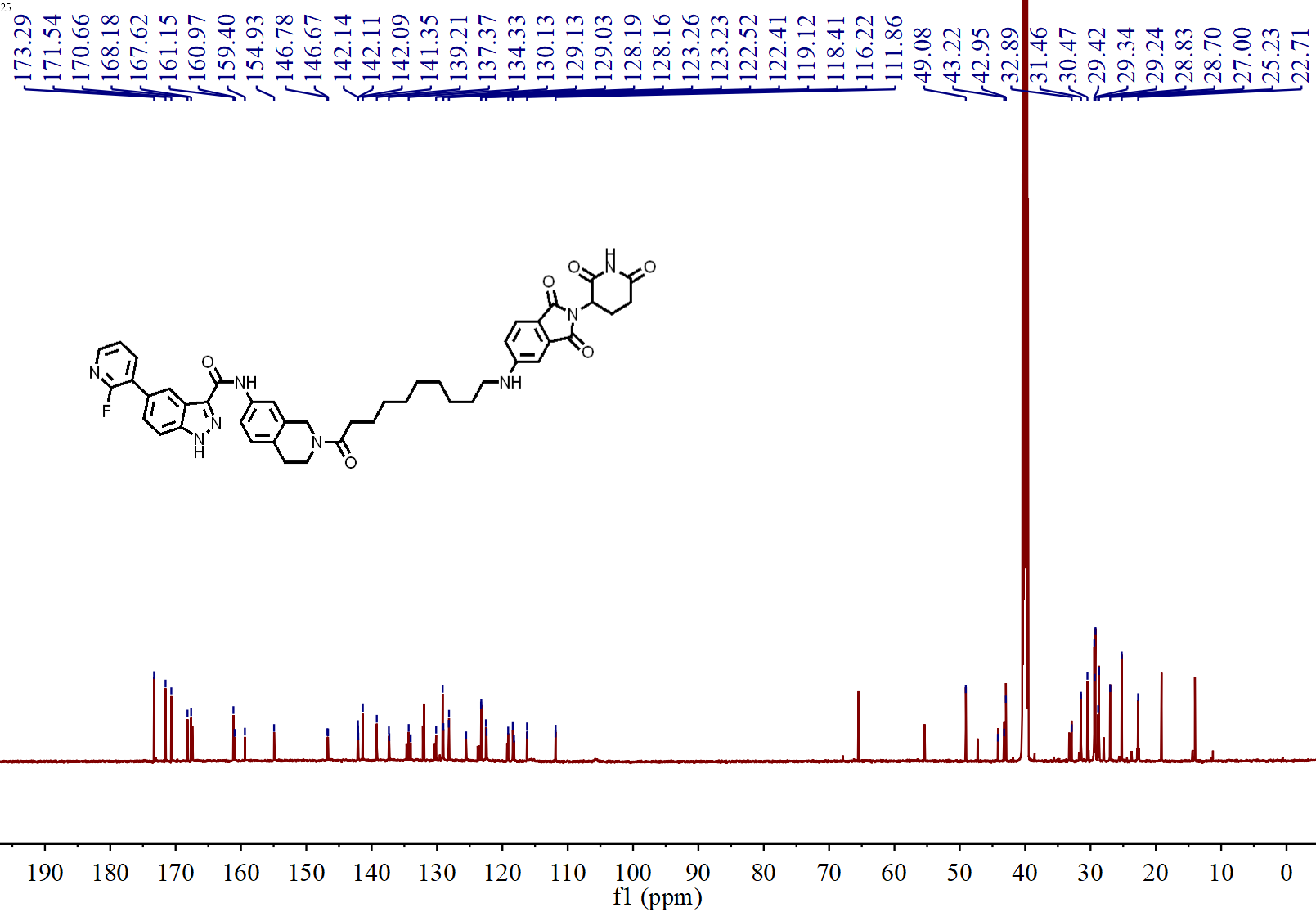


**HRMS Spectra of 5k**

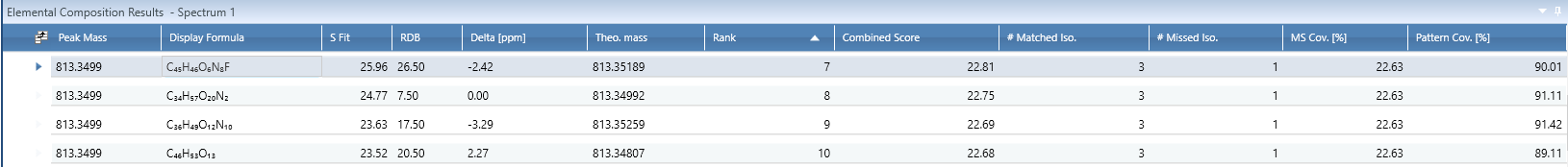


**HPLC Purity Data of 5k**


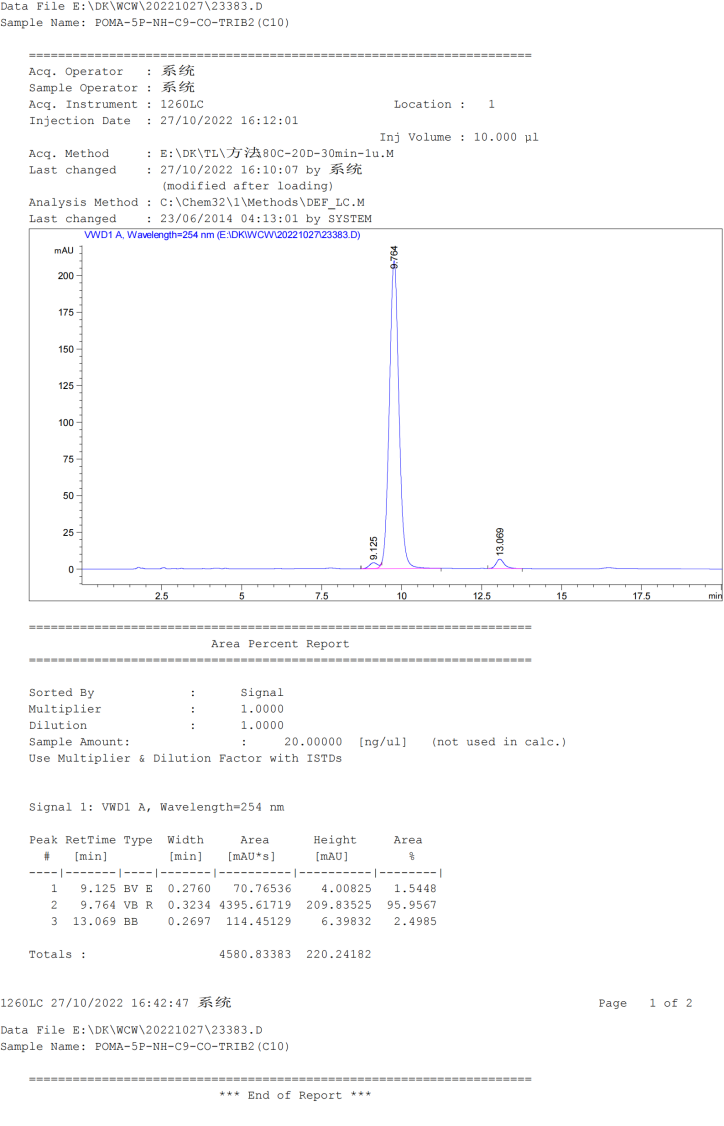


**^1^H and ^13^C NMR Spectra of compound 5l**


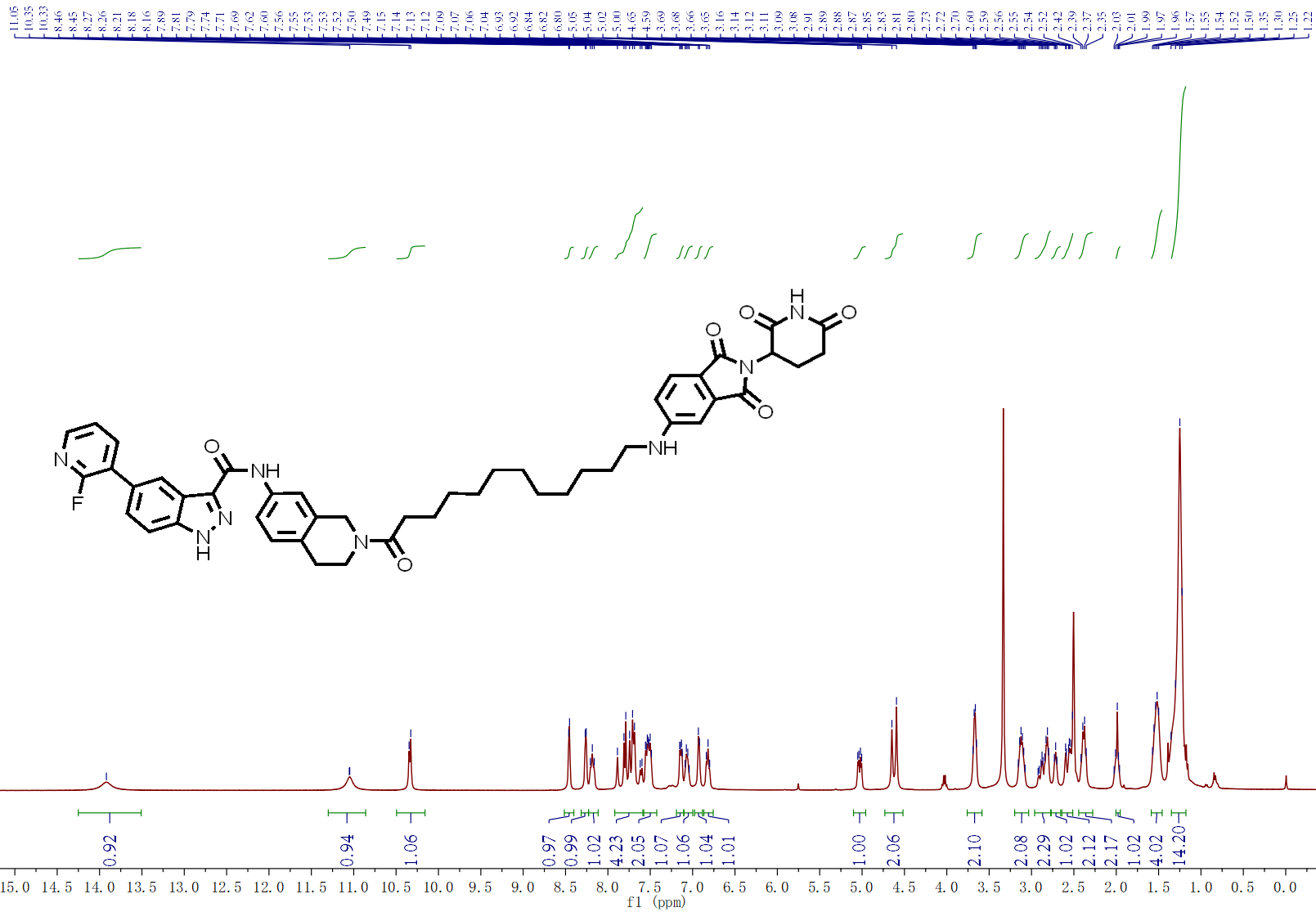

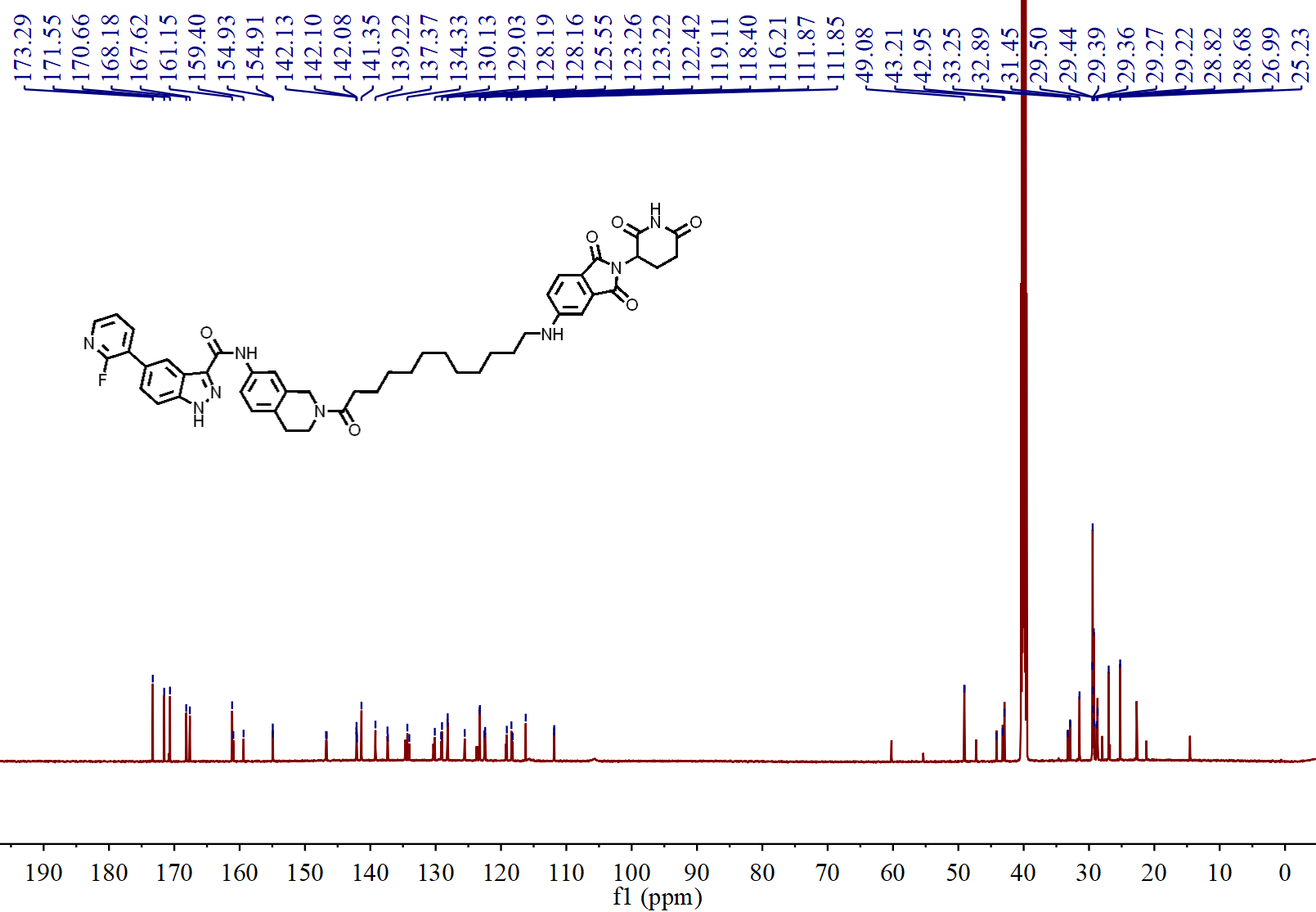


**HRMS Spectra of 5l**

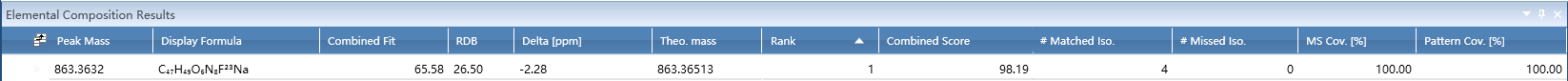


**HPLC Purity Data of 5l**


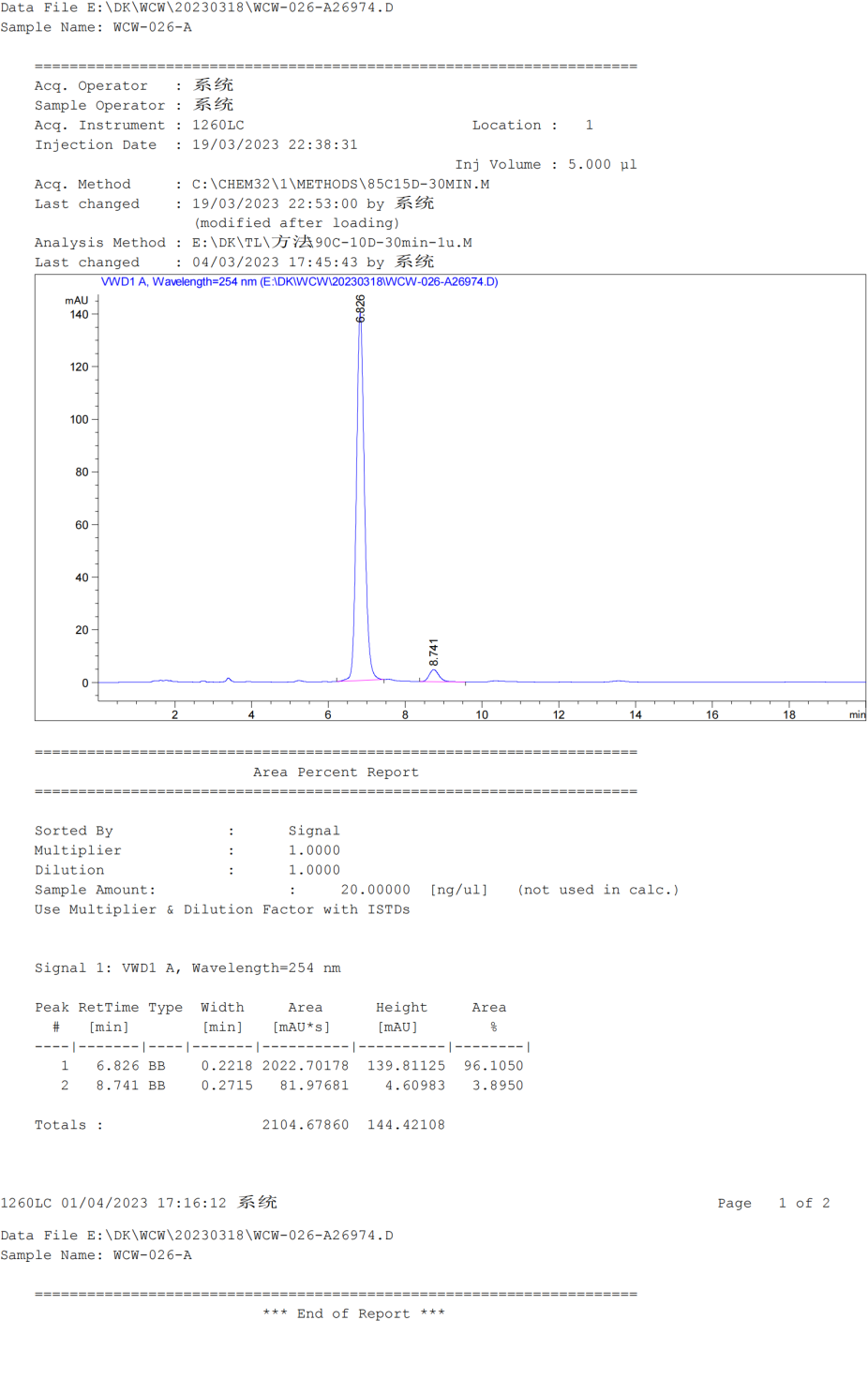


**^1^H and ^13^C NMR Spectra of compound 5m**


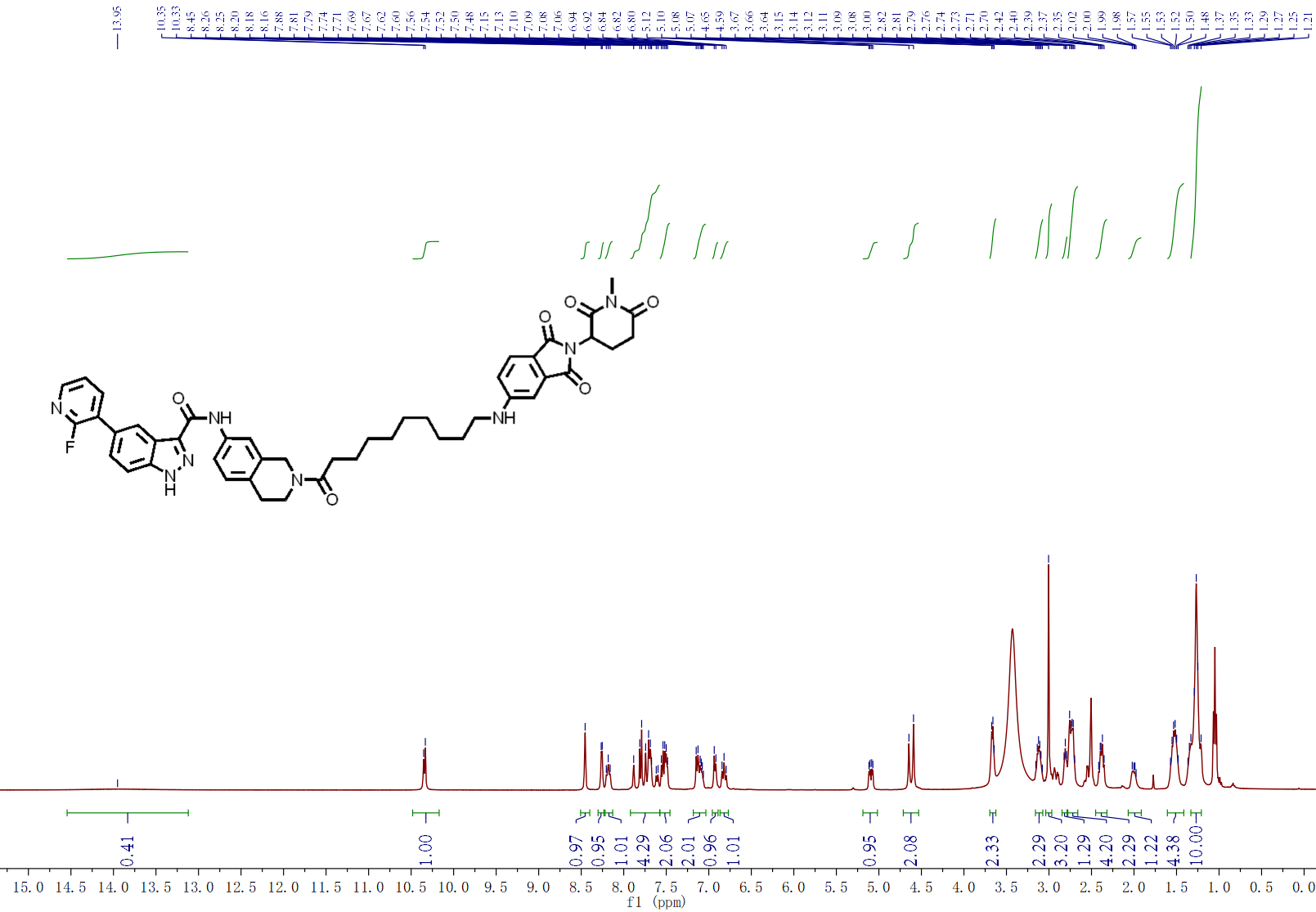

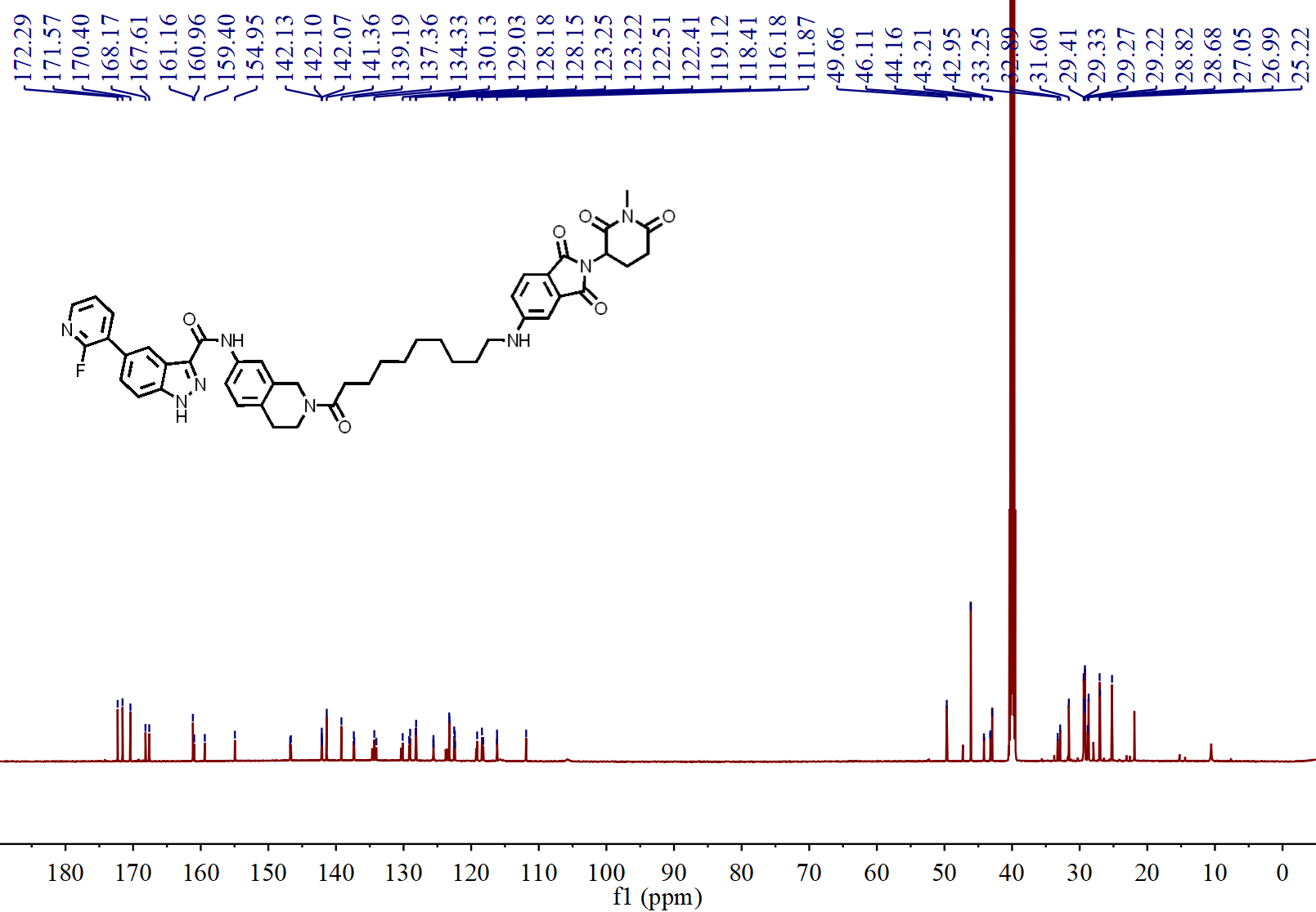


**HRMS Spectra of 5m**


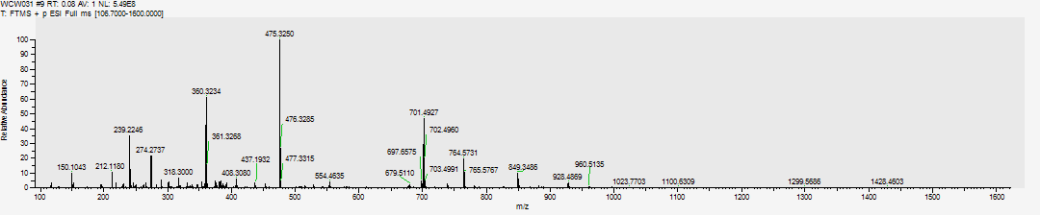


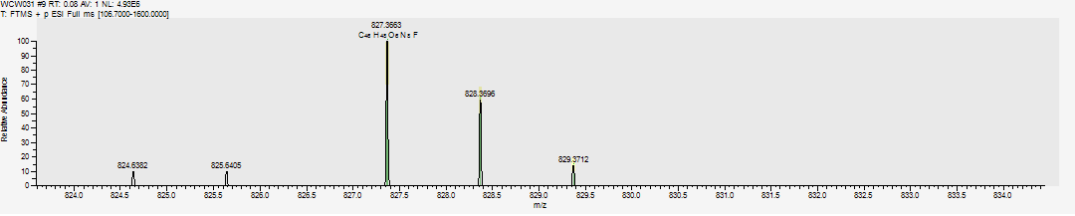

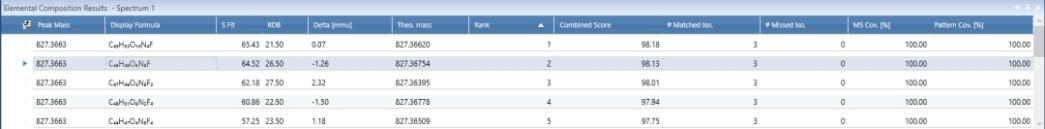


**HPLC Purity Data of 5m**


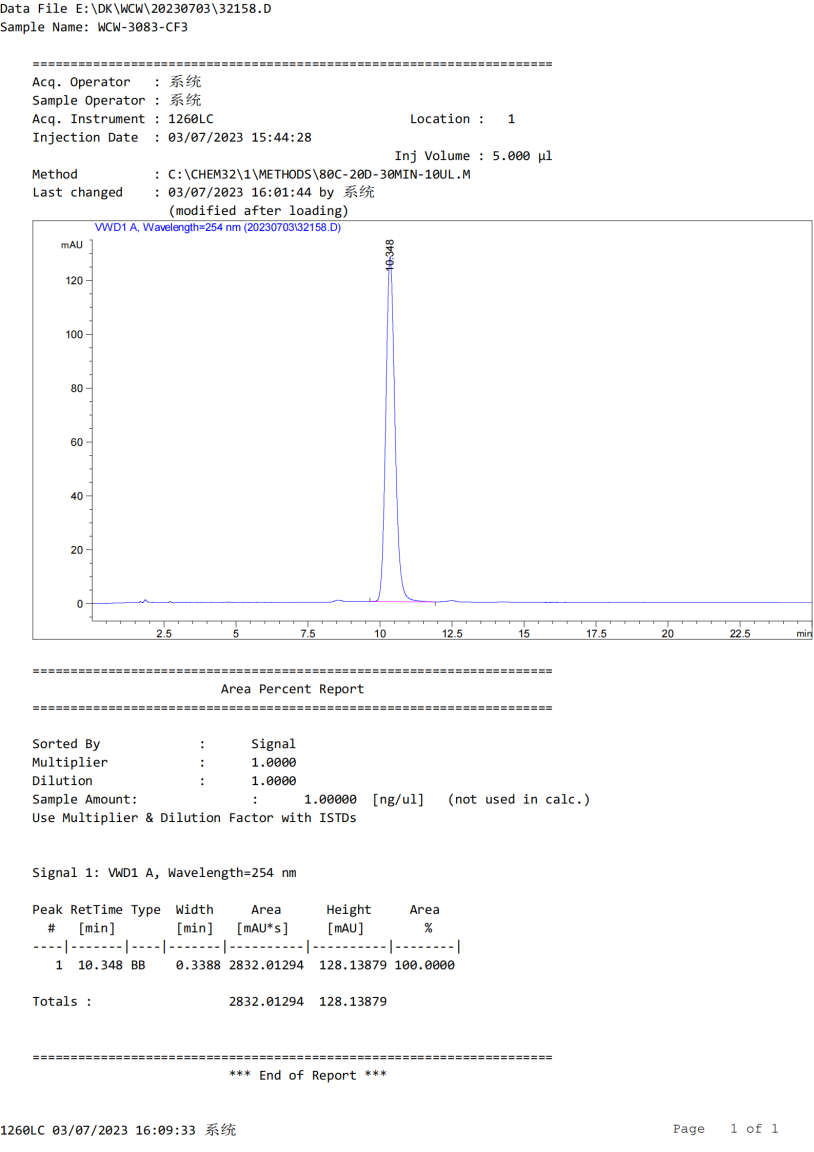


**3. Unprocessed western blots:**

**
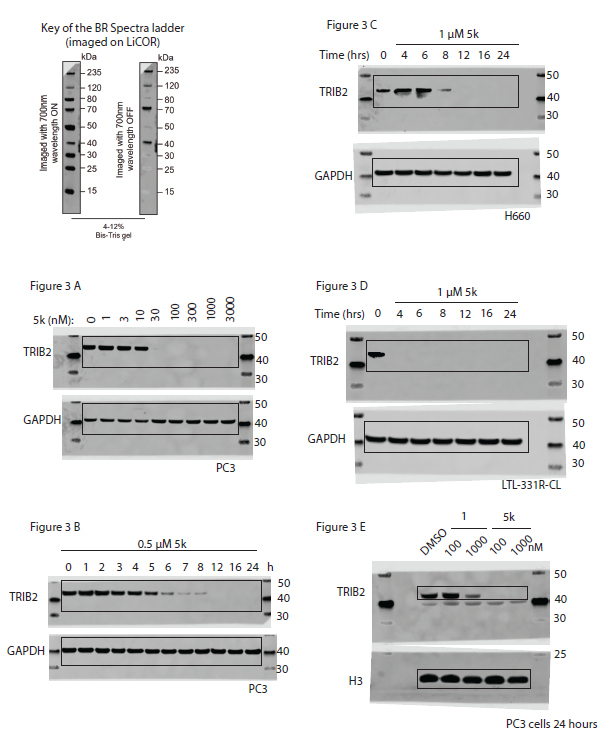
**

**
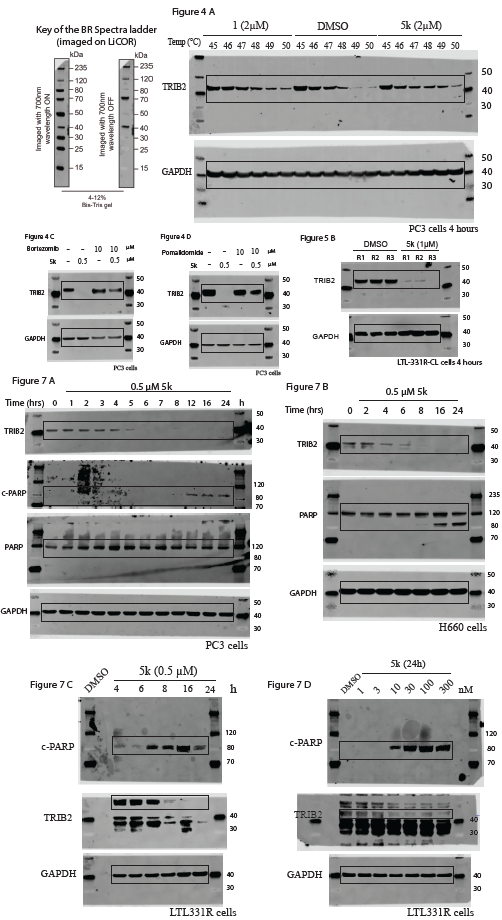
**
